# Providing information to prepare breast cancer patients for their surgical consultation: barriers and recommendations (an analysis from Alliance A231701CD)

**DOI:** 10.1007/s00520-026-10726-7

**Published:** 2026-05-14

**Authors:** Megan C. Saucke, Nora Jacobson, Selina Chow, Grace McKinney, Heather B. Neuman

**Affiliations:** 1 Wisconsin Surgical Outcomes Research Program, University of Wisconsin, Madison, WI USA; 2 University of Wisconsin Institute for Clinical and Translational Research, Madison, WI USA; 3 Alliance Protocol Operations Office, University of Chicago, Chicago, IL USA; 4 University of Wisconsin Carbone Cancer Center, K6/142 CSC, 600 Highland Ave, Madison, WI 53792-1690 USA

**Keywords:** Decision-making, Breast cancer, Surgery, Decision aid, Barriers, Information preferences, Patient engagement

## Abstract

**Purpose:**

We conducted Alliance clinical trial A231701CD that provided breast cancer patients a decision aid (DA) before their surgical consultation with the goal of improving engagement in decision-making. We report a mixed-methods analysis from patients who received the DA to understand patients’ experiences in preparing for their consultation.

**Methods:**

Patients were included in this analysis if they were randomized to the DA arm (*n* = 331) and agreed to receive the DA (*n* = 195). Data collection included surveys before and after the consultation that assessed whether patients reviewed the DA and found it helpful, and reasons for non-review. We interviewed a subset of patients who had low engagement and experienced a preparatory barrier. We used descriptive statistics to summarize survey responses and content analysis to analyze interview data.

**Results:**

Seventy-three percent (*n* = 143) reviewed the DA before the consultation. The majority (93%) said they would recommend receiving information via email. The most common reasons for non-review were logistical. Additional barriers included wanting to hear information directly from the surgeon, feeling they were already informed, wanting to stay in denial about their cancer, and perceiving information would be scary. Interviewees shared suggestions for clinics to optimize patients’ review of information, such as explaining how preparing benefits patients and acknowledging emotion around cancer diagnoses.

**Conclusion:**

Our study highlights that breast cancer patients want information prior to the consultation, but patient-centered challenges can lead to non-review. When sharing pre-consult information, clinics should communicate how reviewing information can help patients feel less overwhelmed and improve their discussion with the surgeon.

**Trial registration:**

ClinicalTrials.gov Identifier: NCT03766009 (12/4/2018).

**Supplementary Information:**

The online version contains supplementary material available at 10.1007/s00520-026-10726-7.

## Introduction

Breast cancer surgery is highly preference sensitive, and patient engagement is critical to reach a high-quality decision aligned with patient values. One of the key challenges to patient engagement in decision-making is the differential knowledge between the patient and clinician [1]. This difference contributes to a power imbalance that is further exacerbated by the emotional and vulnerable nature of the cancer diagnosis [2]. While closing the knowledge gap will not solve this power imbalance, providing information to patients newly diagnosed with cancer can increase awareness of the treatment options, making them better able to understand information shared by the surgeon, ask questions, and contribute to treatment decisions [1, 3–5]. This information may also reduce patient anxiety by giving them insight into anticipated treatment outcomes [6, 7]. Providing patients with high-quality information about their treatment options is an important step in empowering them to actively participate in discussions about their care.

Patients newly diagnosed with breast cancer encounter challenges when attempting to access information about their cancer, such as difficulty finding relevant information as well as information overload [8]. Based on our preliminary work, we anticipated that delivery of information from the clinic to the patient during the “informational gap” between diagnosis and the surgical consultation would facilitate patient preparation for treatment decisions and benefit both patients and providers. Patients have reported improved knowledge, increased feelings of being informed, and reduced distress and decisional conflict when they reviewed clinic-provided information and/or internet information before seeing the surgeon [7, 9–11]. Providers have described facilitators to breast cancer treatment decision-making to be patients being prepared for the consultation and receiving written information; they cite that the information may help with patient misconceptions, anxiety, and not understanding information during the appointment [12].

We conducted the Alliance for Clinical Trials in Oncology trial A231701CD, which provided patients with a web-based DA prior to their surgical consultation with the goal of improving patient engagement in decision-making [13–15]. DAs are tools for “sharing evidence about treatment and screening options and helping patients clarify their values” [16]. The implementation strategies utilized in the trial to deliver the DA were selected to address challenges to the use of a DA in clinical practice reported in the literature [3, 17–23] and experienced in our own pilot work [24, 25]. Central to our implementation approach was to deliver the web-based DA directly to patients soon after diagnosis, thereby addressing challenges patients cite about finding trustworthy, relevant information.

Here, we report a mixed-methods analysis of data collected from patients with breast cancer randomized to the DA arm of Alliance A231701CD to understand patients’ experiences in preparing for their surgical consultation, including their likelihood of reviewing the DA and reasons for forgoing or limiting review of the clinic-provided information. We were specifically interested in the experiences of those patients who had low engagement in the surgical consultation despite having received the DA. A secondary goal was to elicit suggestions from patients regarding strategies that clinics can employ to encourage their patients to review information prior to the surgical consultation.

## Methods

### Study context

Alliance A231701CD was a stepped wedge clinical trial conducted within the NCI Community Oncology Research Program to test a DA’s effectiveness at increasing patient engagement in decision-making (see Appendix for clinical trial protocol) [13–15, 26]. Patient accrual occurred from 7/2019 to 12/2021 in ten clinics across the USA that cared for a high proportion of socioeconomically disadvantaged patients [13, 27]. This study reports the findings of a planned secondary outcome analysis of this clinical trial.

The research team conducted site visits to support clinics’ implementation of the web-based DA into clinical flow [24, 25]. Clinic staff offered the DA to patients prior to the surgical consultation (usually by telephone) and then emailed the DA link. Data collection for the clinical trial included a patient survey at consent (right before the surgical consultation), audio-recording of the consultation, and a patient survey sent the day after the consultation. In this manuscript, the data from the audio-recording and the post-consultation survey were only used to inform sampling for the interview cohort.

### Survey cohort

Patients were eligible for inclusion in this analysis if they were randomized to the DA arm of the intervention (*n* = 331) and agreed to be sent the DA (*n* = 195).

### Survey measures relevant to this analysis

Survey data collection for the clinical trial as a whole is included in the protocol (Appendix). On the survey prior to the surgical consultation, we assessed whether patients reviewed the DA, how long they spent reviewing it, whether they found it to be helpful, and whether they would recommend it to other patients facing a decision for breast cancer surgery. If patients did not review the DA, we examined their free text responses on the survey about why they did not review.

### Interview cohort

We identified patients who experienced low engagement in decision-making despite receiving the DA. Patient engagement was a primary outcome of Alliance A231701CD and was measured in two ways: (1) directly from the surgical consultation through Active Patient Behaviors and (2) through Perceived Efficacy in Patient-Physician Interactions-5 assessed on the post-consultation survey [13]. Patients were eligible for the interviews if they were in the lowest tertile for patient engagement on either measure (*n* = 106) and also indicated barriers to preparing for the consultation (preparatory barrier) [28] and/or barriers to interacting with the surgeon (interactional barrier) [13] (*n* = 88). We used maximum variation sampling, considering patient age, race, area deprivation index [29], clinic, and surgeon, to ensure varied perspectives (*n* = 30). This qualitative analysis focuses on 22 patients who experienced a preparatory barrier, such as indicating on the pre-consultation survey that they felt uninformed about their treatment options.

### 1Interview data collection

Interviews were conducted via telephone or video call after patients completed their surgical and radiation cancer treatment. Interviews began by asking patients to reflect on to their diagnosis and describe their initial reactions (see Interview Guide in Appendix). We also reviewed patients’ pre- and post-consultation survey data in advance of the interview and asked patients to expand on survey responses that indicated barriers to preparing for the consultation. The interviews explored barriers we had defined when the clinical trial was conceptualized and included review of the decision aid or information, patient’s preferred role in the consultation, and how informed they felt going into the consultation (see additional details in the trial protocol in the Appendix). We ended the interviews with an open-ended question of “what else should I know about your experience with making the decision for breast cancer surgery?” All interviews were audio-recorded and transcribed verbatim.

### Analysis

We used descriptive statistics to summarize patients’ characteristics and survey responses. We used inductive qualitative content analysis to analyze the interview data [30, 31]. Early interviews were annotated independently by three investigators (MS, HN, NJ) to generate a preliminary list of codes that were used to reduce the data. We then compared and discussed the identified coding schemes to reach consensus about a coding taxonomy, which guided primary coding of subsequent interviews. As new concepts emerged during subsequent interviews, the coding taxonomy was revised and earlier interviews were recoded. We used visual displays to categorize the data and organize it into broad overarching themes [32]. *NVivo* (QSR International-Melbourne) was used to manage data and facilitate analysis. Patient quotes have been lightly edited for readability (e.g., removed filler words).

## Results

The majority of Alliance A231701CD participants who were sent the DA (*n* = 195) were white (69%) with a college degree (63%) (Table 1). The median age was 61 years and 17% lived in a socioeconomically disadvantaged area. There were no statistically significant differences in the demographics between the 195 patients who agreed to be sent the DA and the 22 interviewed patients.

**Table 1:** Characteristics of Alliance A231701CD patients who were sent the decision aid and who were sent the decision aid and participated in interviews

	Sent the decision aid *n* = 195	Sent the decision aid and participated in interview *N* = 22
Age (median, range)	61 years (28–88)	63 years (38–72)
Race		
White Black Other	123 (69%) 26 (14.5%) 31 (17%)	15 (68%) 7 (32%) 0 (0%)
Socioeconomic disadvantage	33 (17%)	6 (27%)
Education		
High school or less Vocational degree or some college College degree Graduate school degree	33 (18.5%) 33 (18.5%) 68 (38%) 44 (25%)	4 (19%) 6 (29%) 8 (38%) 3 (14%)
Clinical T stage		
T0 T1 T2 T3/T4	37 (19.0%) 93 (47.7%) 46 (23.6%) 19 (9.7%)	2 (9.1%) 9 (40.9%) 7 (31.8%) 4 (18.2%)
Clinical N stage		
N0 N1/N2/N3 NX	160 (82.1%) 29 (14.9%) 6 (3.1%)	15 (68.2%) 6 (27.3%) 1 (4.5%)
Reviewed DA prior to visit	143 (73%)	13 (62%)

### Findings from quantitative analysis of survey data

Of the 195 patients who agreed to be sent the DA, 73% (*n* = 143) reviewed the DA prior to the surgical consultation. Most reviewed the DA for at least 15 min (56%, 15–60 min, and 26%, > 60 min). The majority (93%) said they would recommend receiving breast cancer information via email to other patients facing the decision for breast cancer surgery. In addition, patients largely found the DA to be helpful (median score of 9 on a 10-point scale, inter-quartile range 7–10).

Among the 52 patients who received the DA but did not review it prior to the consultation, the most common reasons for non-review could be considered logistical challenges (38%), such as issues with email delivery of the DA (*n* = 5), issues related to personal computer or internet (*n* = 5), trouble with the DA website itself (*n* = 7), or not receiving the DA far enough in advance of the surgical visit to have time to review (*n* = 3). Two patients cited not being “good with computers.” A sizable proportion (31%) stated they did not have the time, were too busy, or forgot. Several other patients reported feeling “too stressed” or stated they wanted to talk to the doctor first.

### Findings from qualitative analysis of interview data

Our interviewees, selected because of overall low engagement in the consultation, talked in more depth about barriers to preparing for their surgical consultation. Of our 22 interview participants, 8 (36%) did not review the DA prior to the consultation. However, even those patients who reviewed the DA described reasons for limiting the time they spent reviewing information (Table 2).

**Table 2:** Barriers to preparing for surgical consultation

Preferring to hear information and options directly from the surgeon	“Honestly, I really didn’t care to receive the information from the [DA] website…. I want it to almost come just straight from the horse’s mouth, you know?” (Patient 21) “I tried not to Google… I wanted to go in with an open mind… and wanted to visit with my surgeon on what he thought my best options were, because that’s their job… And to be flexible, because you can go in thinking one thing’s going to happen.” (Patient 25) “If I knew more [going in], and the doctors did end up telling me something totally different, I would feel let down if it…was really much worse than what I had anticipated. You know, I would rather just be disappointed once and be upset once.” (Patient 18)
Assuming they are already informed	“I was just going off what I already knew about having a mastectomy [from two acquaintances who had breast cancer]. Actually, I didn’t know or realize that the cancer can be removed without having a mastectomy honestly until [SURGEON] explained to me that option.” (Patient 21) “I just remember my aunt, she had to have both radiation and chemo. And then she had very low platelets… That was like 40 years ago. So in my mind, all those things would end up happening to me.” (Patient 18)
Wanting to stay in denial/perceiving information to be scary or overwhelming	“If I don’t have to go look at this, then maybe I can put off the realness of the whole situation just a little longer. And not have to really comprehend or understand exactly what was coming my way.” (Patient 25) “Not knowing the details was better than looking at it and seeing that it was much worse than I thought. It was sort of was like I lived a fantasy.” (Patient 18) “Maybe I didn’t want to be more informed because maybe it would have scared me…. I mean, sometimes too much knowledge is a bad thing.” (Patient 1)
Finding information too overwhelming to review in depth	“When you get on the internet like that, without really any knowledge of what you have to do, or from your oncologist, it’s just, you can kind of start spiraling and start thinking the worst. Um, so it was a horrible 6 days.” (Patient 4) “It was kind of scary, you know, looking at all the drawings and things like that, it was just not my cup of tea exactly.” (Patient 2)

First, several patients reiterated the desire to hear information about their cancer directly from their surgeon, face-to-face, rather than looking at clinic-provided materials or other information online. They said they trusted the surgeon to share necessary information about the options that were relevant to them, so they did not feel a need to prepare for the consult. Some patients did not want to learn too much beforehand, because they did not want to have preconceived notions about their treatment preferences before hearing from the surgeon. In particular, they were afraid of getting too attached to one treatment option and being disappointed if the surgeon said they were not a candidate for that option.

Next, a number of patients said they did not look at the information because they assumed they were already well informed about treatment options. These patients mentioned that they knew family members or friends who had gone through breast cancer treatment and felt they were already informed based on their experiences. However, we observed that their acquaintances’ experiences were often a decade or more earlier and were often distinct clinical scenarios.

In addition, some patients acknowledged that they did not look at information before their consult in order to avoid or reduce negative emotions about their cancer such as anxiety, fear, and worry. Some specifically said they wanted to delay facing the reality of their cancer diagnosis. One patient acknowledged that she knew the clinic recommended the DA and that it was “not designed to give a worst case scenario.” However, she still avoided reviewing the DA so as to stay in denial about her cancer. Patients also shared concerns that the information would be frightening and avoided information because of “not wanting to scare the crap out of myself” (Patient 25). Some described specifically being fearful that they would see upsetting or “gory” images or learn details that would put them in a negative headspace.

Finally, some interviewed patients attempted to review information to prepare for their appointment but stopped when they found the information to be overwhelming or confusing. One patient described searching online and reading as much as she could, with the information triggering catastrophic thinking. Patients also expressed being unsure what was true online. While these issues were more common for patients who went down internet “rabbit holes,” they also arose for some patients who reviewed clinic-sanctioned resources. One patient who visited the DA website felt overwhelmed by the number of videos available and stopped after watching one or two. Similarly, another patient who received a book provided by the clinic said she felt “overwhelmed” after seeing all of the options and different treatment trajectories.

### Patient expressions of regret regarding limited preparation for consult

Many interviewed patients expressed regret at not preparing for the surgical consultation. One patient said, “in hindsight, I should have reviewed it [DA] so that I knew more about what the doctor wanted to talk to me about” (Patient 25). One patient who had avoided the DA prior to the consultation out of fear said she would tell her past self, “take a look at it, you dummy” and acknowledged that the information was “much less scary or drastic than what I had in my mind” (Patient 18). One patient who went down an internet rabbit hole before her consultation said when she finally looked at the DA: “I remember thinking like, oh, ‘this is a really good description. These are good videos’” (Patient 4). Despite their regret, patients reiterated that their emotional state was a key factor in limiting their preparation for their consultation. “That’s one of the things that I regret, not reading more of the information… but I had so much anxiety during that time that I didn’t know what to do” (Patient 5).

### Suggestions for clinics when encouraging patients to prepare for consultation

Patients shared suggestions for clinics to encourage patients to prepare before their surgical consultations, especially for those patients who may be experiencing strong emotions about their diagnosis. When asked what she would tell someone who is scared to look at the DA, one patient said she would acknowledge the patient’s emotional state and emphasize the importance of reviewing the information so that she could ask better questions during the consult:

I understand your anxiety. I understand that you don’t want to hear more information because you feel that you’re going to be confused, but this comes directly from pretty much the doctor… I believe that this is going to help you if you really look at it. (Patient 4)

Some patients thought the clinic should tell patients that the information is meant to be reassuring, not scary. One patient who had been concerned that the DA would be “gory” said “I didn’t know if they showed actual pictures of surgery before and after, but if it isn’t like that… maybe if you let them know it’s not as graphic or as bad as they may think it is” (Patient 18).

Patients also recommended that the clinic should remind patients to look at the information, because they may be too overwhelmed to remember.

You get so much information at once… and you’re so overwhelmed that at that moment trying to understand what’s going on that you just put it aside. So a reminder that, ‘Hey, have you looked into this?’ (Patient 4)

Patients thought that clinics should encourage and remind them to review the information even after the visit. One patient who got overwhelmed by the DA videos prior to the consultation returned to the DA afterwards and said the information relieved anxiety about what would happen next and “really helped me to like, calm down and really understand what the doctor was telling me” (Patient 5).

Patients also suggested flexibility about the format of information given to patients. They felt that it may be beneficial if the information is available in multiple formats including paper, DVD, and a website. Second, they suggested that the information and the DA video in particular should be presented at multiple times, with a few patients even wishing they had some information before they actually had a cancer diagnosis:

There are so many weeks in between from the ultrasound and biopsy… it’s not cancer for sure, but if they provide you with this information, you already know what you’re going to go through. And so I really well may have appreciated more before the biopsy, after the biopsy, after that first appointment, like reminders that there is information. (Patient 4)

Lastly, patients recommended that the clinic should share a list of other reputable resources to guide patients who want to do additional learning outside of clinic-provided materials. “Maybe a list of websites to look at? From them, they could have just, ‘we’ll send you a link to something,’ instead of me just looking it up online, so that might have been nice” (Patient 6). Clinics directing patients’ search for additional information could help prevent patients from going down internet rabbit holes that may contain confusing, outdated, or inaccurate information.

## Discussion

Our study provides clinics with valuable insights on how they can encourage patients newly diagnosed with breast cancer to prepare for their surgical consultations. We found that most of the participants in our study were interested in receiving information from their care team during the window between diagnosis and when they meet their surgeon. The majority of women who received the DA spent at least some time reviewing the DA before their consultation and found it to be beneficial. Providing high-quality educational materials, through a DA or other means, is a simple step that clinics can take in supporting patients newly diagnosed with cancer.

Our study also highlights patient-centered challenges to delivering information in the window between diagnosis and the consultation. We observed many of these same challenges in our pilot work and in the literature [33–37]. The most commonly cited reasons for patients’ non-review on the survey were related to logistical issues, such as having a consultation scheduled soon after diagnosis or difficulty getting the weblink to work. Our interviews explored a number of additional patient-centered reasons that contributed to patients not reviewing information in advance of the surgical consultation, such as patients preferring to hear information directly from the surgeon [33–36], wanting to stay in denial [33, 35, 36], or feeling overwhelmed [18, 35–37]. Although these reasons for not preparing for the consultation can be more challenging to address, it is important to address these reasons as patients waiting until the consultation to receive information may have negative consequences to patients’ engagement in treatment decision-making. Many patients, especially those hearing information for the first time, may experience information overload in the consultation, leading to emotional overwhelm that reduces their ability to listen, comprehend new information, and participate in decision-making [2, 5, 12, 38]. However, to address these types of patient-centered challenges, it is not enough to provide patients with information. Instead, clinics will need to use more deliberate implementation strategies [17, 19].

When planning Alliance A231701CD, we included strategies to mitigate barriers to patients reviewing the DA, such as delivering the web-based DA directly to patients soon after diagnosis and phone and email scripts for clinic staff to use to distribute the DA (Table 3, column 2). We found that many barriers persisted in our current study, despite these efforts. It is possible that the strategies we identified and implemented were inadequate to overcome patient barriers to reviewing the DA. However, it is also possible that the persistence of these barriers reflects the COVID-19 pandemic. Alliance A231701CD was conducted from 7/2019 to 12/2021, when clinics experienced significant challenges related to competing priorities and shifting workforce. It is difficult to determine definitively whether our strategies were inadequate, or whether the persistence of barriers is a result of the complexities of delivering cancer care—and being a cancer patient—during the pandemic.

**Table 3:** Original and revised suggested scripts to encourage patients to review information

Patient barrier to reviewing information to prepare for the consultation	Original scripts*	Revised scripts**
Preferring to hear information and options directly from the surgeon	“Dr. X in particular thinks that this information is really good. They think that it is really helpful when patients review the information before seeing them. It won’t change the amount of time your surgeon will spend with you, but reviewing the information does seem to make it easier for patients to talk with your surgeon about what kind of breast surgery might be right for you.” *The website includes text as well as video clips from patients and physicians. There is a lot of important information in the video clips, and we encourage you to take the time to watch the videos. ****Your surgeon really thinks this information is helpful and encourages you to review it before you meet***	Use original text plus this additional language: Reviewing this information before the visit will allow your surgeon to spend more time discussing your specific situation, rather than spending time discussing the basics of breast cancer A lot of patients can feel overwhelmed in their appointment if it’s the first time they’re hearing the information. Reviewing this resource beforehand will help you better understand what your surgeon tells you during your consultation. It may also help you think of the questions you want to ask
Assuming they are already informed	*Although there is a lot of information on the website, “you can spend as much as or as little time as you want reviewing it.” The web-link listed below includes information on a number of different decisions patients face as they plan treatment…. Each information topic gives a brief outline of treatment, as a sort of ‘treatment 101*.’	Lots of patients know someone who had breast cancer and feel like they know what to expect because of that. However, each person is unique and the options for treating breast cancer change rapidly. Your surgeon encourages you to review the information even if you already feel you know what to expect. This can help make sure you have a strong base built on accurate information to start the conversation with your surgeon
Wanting to stay in denial/perceiving information to be scary or overwhelming	“I know that hearing about your new breast cancer diagnosis is overwhelming and that it can be really hard to face a decision for breast cancer surgery. We would like to send you some information about breast cancer surgery that patients have used and really liked in the past. The information is on a website.” If patient declines DA due to being “too overwhelmed”: “I completely understand. I will say that many of our patients have found that this particular website actually made them feel less stressed about the diagnosis. It provided information in a way that made the diagnosis seem less overwhelming. This is definitely something that Dr. X believes.”	We know that you may feel nervous to look at this information. We have heard that from other patients. However, most patients say that it actually was not as scary as they expected and most patients feel better and less worried after looking at this information If you find the information overwhelming, focus first on the basic description of breast cancer so that the words you will hear are familiar. This will help you in the surgeon visit Additional text to consider for patients who decline information due to “being overwhelmed”: I know this is a lot. However, patients who avoided looking at information later said that they regretted not preparing for their appointment and wished they had looked ahead of time
Too busy or forgot	N/A	Resend email delivering the information 48 h after initial email and 24 h before the appointment

*Script implemented through clinic phone call to patients (represented in “quotes”) or email content (represented in *italics*)

**This sample language should be used in both written communication and person-to-person discussions to reinforce the importance of reviewing information before the visit

Our study provides additional insight into opportunities to refine how clinics can deliver educational materials, such as a DA, to patients in order to optimize uptake. In Table 3, column 3, we provide language clinic staff can use when disseminating clinic-approved information to pre-emptively address identified barriers. The table includes revisions to strategies we used in this trial and new strategies to address barriers identified through this work. Given patients’ concerns that reviewing information will exacerbate negative feelings [33, 35, 36], acknowledging the patient’s emotional state and communicating the potential emotional benefits of reviewing the information are especially important. Communications should not only emphasize that the information is endorsed by the care team, but also state *why* it is important for the patient to review, explaining how the consultation will go better if the patient has a basic, up-to-date understanding of the cancer and the treatment options. Since patients want to hear information directly from their surgeon [33–36], patients should be told that their surgeon endorses the information and reassured that preparation does not take away from the time spent with their surgeon but rather makes that time more effective. Clinics should also remember that patients newly diagnosed with cancer are overwhelmed and struggling to integrate the diagnosis into the other competing demands of their daily lives. Recognizing this, clinics should provide patients with multiple prompts to review the information so that patients know it is a priority and do not forget.

We believe some interviewee suggestions cannot be accommodated. First, patients suggested distributing information in multiple formats, such as mailing a DVD and/or printed information. Due to the tight timeline between breast cancer diagnosis and clinic visits, sending information through the mail would not give patients enough time to receive the information before their visit. Second, patients suggested distributing information before the patient even receives a cancer diagnosis. However, this may have an unintended negative impact on patients, given that the majority of breast biopsies are benign.

A strength of our study is that it reflects the perspectives of a diverse cohort of women newly diagnosed with breast cancer. Our findings reflect the perspectives of patients who responded to the survey or participated in the interview, creating the potential for participation bias. However, the barriers described in the survey and interviews impact a sizable proportion of patients, and implementing strategies to address them is valuable. The cohort of patients presented in this study is somewhat more educated and affluent compared with the national average, and this should be considered when considering our findings. However, our cohort is more diverse than is typical for clinical trial participants. We conducted the interviews after treatment was complete, which introduces the possibility of recall bias when describing reasons for not preparing for the appointment. However, the survey responses were much more proximal to the treatment and were used to prompt the interview questions, which minimizes this risk. This study focuses only on the cohort of patients who agreed to be sent the decision aid. Perspectives of those who may not have been offered the decision aid by the clinic or who were offered the decision aid but declined are not reflected in this study. Finally, the conduct of the study during the COVID-19 pandemic limits our ability to assess the impact of our pre-planned implementation strategies. However, 73% of patients did review the DA prior to the visit which suggests that our approach was effective overall.

## Conclusions

Patient engagement is important for making decisions that align with patients’ values. Patient preparation for the consultation benefits both the patient and the surgeon, as the focus of the consultation can shift from information provision to deliberation with the patient about the treatment options that are right for them. In our study, patients endorsed that receiving information between diagnosis and the surgical consultation was beneficial and would recommend this approach to other patients. Further, we demonstrated that provision of information in the time between diagnosis and the consultation is feasible, although challenging. Our findings in this study identified key challenges to patient preparation that are important to consider to ensure maximum reach of pre-visit information.

## Supplementary Information

Below is the link to the electronic supplementary material.

ESM1 (PDF 2.11 MB)

## Data Availability

Data will be available by request per the policy of the Alliance for Clinical Trials in Oncology (https://www.allianceforclinicaltrialsinoncology.org).
