# Supplementary material for: Providing information to prepare breast cancer patients for their surgical consultation: barriers and recommendations (an analysis from Alliance A231701CD)
Source: Support Care Cancer. 2026 May 14;34(6):537. doi: 10.1007/s00520-026-10726-7 (PMC13171935; doi:10.1007/s00520-026-10726-7)
Supplement: Supplementary file 1 — (PDF 2.11 MB) [file 520_2026_10726_MOESM1_ESM.pdf]

ALLIANCE FOR CLINICAL TRIALS IN ONCOLOGY
ALLIANCE A231701CD

INCREASING SOCIOECONOMICALLY DISADVANTAGED PATIENTS' ENGAGEMENT IN BREAST CANCER SURGERY DECISION MAKING THROUGH A SHARED DECISION MAKING INTERVENTION

A limited access study

ClinicalTrials.gov Identifier: NCT03766009

Study Chair
Heather B. Neuman, MD, MS
Department of Surgery
University of Wisconsin School of Medicine and Public Health
600 Highland Ave
Madison, WI
608-265-5852
neuman@surgery.wisc.edu

Community Oncology Co-chair
Lisa Bailey, MD
Bay Area Tumor Institute NCORP
BaileyLisa@msn.com

Health Disparities Co-Chair
Marilyn Leitch
Univ. of Texas Southwestern Med. Center
Marilyn.Leitch@UTSouthwestern.edu

CCDR Committee Co-Chairs
George J. Chang, MD, MS
MD Anderson Cancer Center
gchang@mdanderson.org

Heather B. Neuman, MD, MS
University of Wisconsin
neuman@surgery.wisc.edu

Health Disparities Committee Chair
Lucile Adams-Campbell, PhD
Georgetown University
lla9@georgetown.edu

Statistician
Paul Rathouz PhD
University of Texas
paul.rathouz@austin.utexas.edu

Statistician
David Zahrieh, PhD
Mayo Clinic
Zahrieh.David@mayo.edu

Data Manager
Patricia McNamara
Mayo Clinic
Tel: 507-266-3028
mcnamara.patricia@mayo.edu

Protocol Coordinator
Rachel Wills
Tel: 773-702-9814
rwills@bsd.uchicago.edu

Limited Access Institutions

University of New Mexico M/U NCORP NM004
VCU Massey Cancer Center M/U NCORP VA010
Medical University of South Carolina M/U NCORP SC008
Georgia Cares M/U NCORP GA020
Hawaii M/U NCORP HI012
Stroger Hospital of Cook County M/U NCORP IL042
Wisconsin NCORP WI031
Montana Cancer Consortium NCORP MT002
Carle Cancer Center NCORP IL168
Bay Area Tumor Institute NCORP CA671

Participants: NCORP components of the Alliance (lead), ECOG-ACRIN, NRG, and SWOG NCORP Research Bases

Study Resources:

**Medidata Rave® iMedidata portal**
https://login.imedidata.com
**OPEN (Oncology Patient Enrollment Network)**
https://open.ctsu.org

Protocol Contacts:

A231701CD Nursing Contact
Mary Beth Wilwerding, RN
Nurse Navigator, Oncology Research
Methodist Estabrook Cancer Center
Tel: 402-354-5831
Email: marybeth.wilwerding@nmhs.org

Protocol-related questions may be directed as follows:

**Questions**	**Contact (via email)**
Questions regarding patient eligibility, treatment, and dose modification:	Study Chair, Nursing Contact, Protocol Coordinator, and (where applicable) Data Manager
Questions related to data submission, RAVE or patient follow-up:	Data Manager
Questions regarding the protocol document and model informed consent:	Protocol Coordinator
Questions related to IRB review	Alliance Regulatory Inbox<br>regulatory@allianceNCTN.org
Questions regarding CTEP-AERS reporting:	Alliance Pharmacovigilance Inbox<br><br>pharmacovigilance@alliancenctn.org

CANCER TRIALS SUPPORT UNIT (CTSU) ADDRESS AND CONTACT INFORMATION

**For regulatory requirements:**	**For patient enrollments:**	**For data submission:**
Regulatory documentation must be submitted to the CTSU via the Regulatory Submission Portal.<br>(Sign in at www.ctsu.org, and select the Regulatory > Regulatory Submission.)<br><br>Institutions with patients waiting that are unable to use the Portal should alert the CTSU Regulatory Office immediately at 1-866-651-2878 to receive further instruction and support.<br><br>Contact the CTSU Regulatory Help Desk at 1-866-651-2878 for regulatory assistance.	Refer to the patient enrollment section of the protocol for instructions on using the Oncology Patient Enrollment Network (OPEN). OPEN is accessed at https://www.ctsu.org/OPEN_SYSTEM/ or https://OPEN.ctsu.org.<br><br>Contact the CTSU Help Desk with any OPEN related questions by phone or email : 1-888-823-5923, or ctsucontact@westat.com.	Some data collection for this study will be done through Medidata Rave and other data submission will be done directly to the University of Wisconsin. Refer to the data submission section of the protocol for further instructions.

The most current version of the **study protocol and all supporting documents** must be downloaded from the protocol-specific page located on the CTSU members' website (https://www.ctsu.org). Access to the CTSU members' website is managed through the Cancer Therapy and Evaluation Program - Identity and Access Management (CTEP-IAM) registration system and requires log in with a CTEP-IAM username and password.

Permission to view and download this protocol and its supporting documents is restricted and is based on person and site roster assignment housed in the CTSU Regulatory Support System (RSS).

For clinical questions (i.e., patient eligibility or treatment-related) see the Protocol Contacts, Page 2.

For non-clinical questions (i.e., unrelated to patient eligibility, treatment, or clinical data submission) Contact the CTSU Help Desk by phone or email:

CTSU General Information Line – 1-888-823-5923, or ctsucontact@westat.com. All calls and correspondence will be triaged to the appropriate CTSU representative.

INCREASING SOCIOECONOMICALLY DISADVANTAGED PATIENTS' ENGAGEMENT IN BREAST CANCER SURGERY DECISION MAKING THROUGH A SHARED DECISION MAKING INTERVENTION

Required Initial Lab Values
None

Patient Eligibility Criteria: (See Section 3.2)

- Women newly diagnosed with clinical stage 0-III breast cancer.
- Eligible patients must be planning breast surgery as a component of their definitive treatment.
- Patients with impaired decision-making capacity are not eligible for this study.
- Patients with hearing impairment requiring the use of an interpreter are not eligible for this study.
- Patients must be able to speak English (see Section 3.2.5)
- Age ≥ 18 years

PATIENT SCHEMA

For patients enrolled prior to Institutional Crossover:

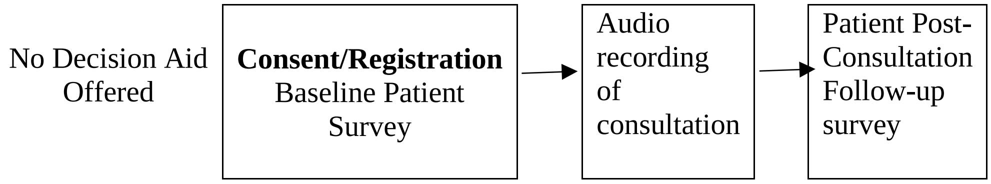

For patients enrolled after Institutional Crossover:

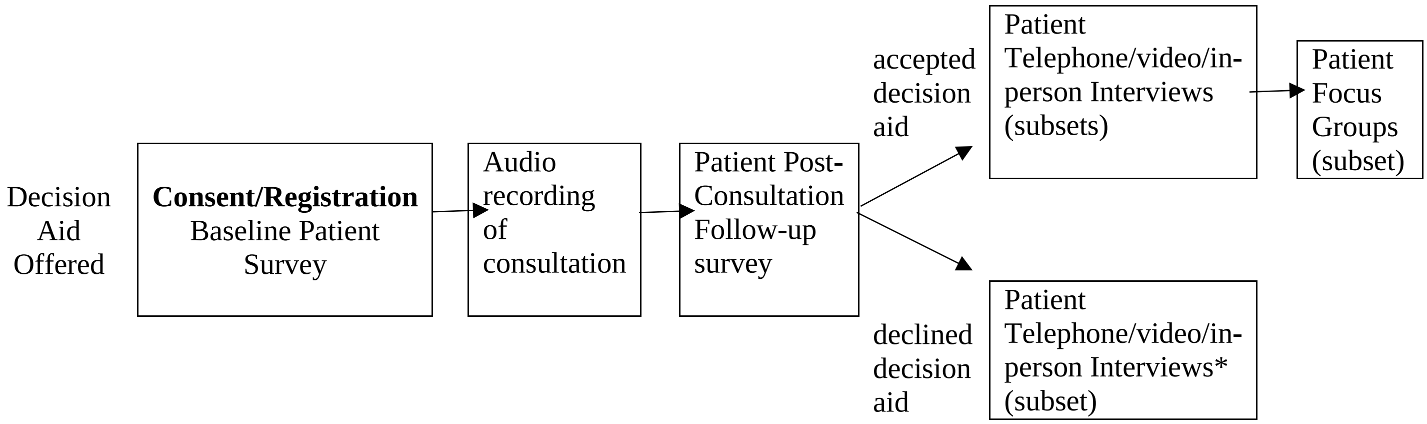

* A subset of patients who decline the decision aid and did not participate in the surveys and audio recording of the consultation may also be approached for a telephone/video/in-person interview alone.

Clinic Stakeholder (Surgeons and Clinic Staff) Eligibility Criteria: (See Section 3.3)
Breast surgeon(s) and nursing staff, medical assistant, or mid-level provider at each participating clinic who participate in the care of patients newly diagnosed with breast cancer.

Surgeon Schema†

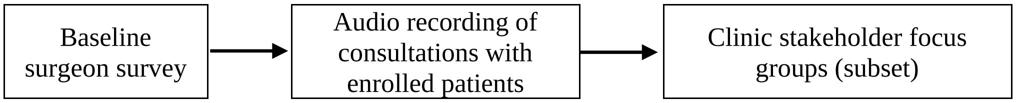

Institutional Eligibility Criteria: (See Section 3.4)

Eligible clinics will serve a high proportion of socioeconomically disadvantaged patients, defined using two data sources. Using these data, 10 clinics that annually provide surgical care for 120-300 patients newly diagnosed with breast cancer will be selected to participate in this study. Surgeons at eligible clinics must consent to the study as a requirement for site participation.

All participating institutions will be selected by the study team per Section 3.4 prior to study activation.

Clinic Level Schema

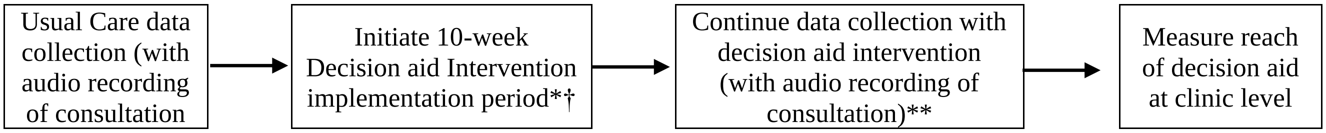

* Sites are randomized to timing of cross-over from usual care to decision aid intervention.
** Same data are collected before and after cross-over to decision aid

† Surgeons will be asked to engage in the decision aid implementation process during the cross-over implementation period.

Study Timeline

	**Year 1**				**Year 2**				**Year 3**				**Year 4**			
Determine and randomize clinic participants (Activation)	X															
Baseline survey to surgeons	X															
Begin quantitative data collection in usual care clinics		X														
Clinic visits to initiate implementation processes		X	X	X	X	X										
Ongoing data collection and clinics switching from usual care to implementation			X	X	X	X	X									
Complete data collection with all clinics							X									
Patient interviews: Accessibility barriers							X	X	X							
Patient interviews: preparatory or interactional barriers											X	X	X	X		
Stakeholder focus groups															X	X

TABLE OF CONTENTS

1.0 BACKGROUND 8

1.1 Introduction 8
1.2 Conceptual Model 9
1.3 Gap Addressed in this Study 9
1.4 Study Overview 9
1.5 Design Considerations and Supporting Preliminary Data 10
1.6 Study Design 12

2.0 OBJECTIVES 13

2.1 Co-primary objectives 13
2.2 Secondary objective(s) 13

3.0 PARTICIPANT SELECTION 13

3.1 Patient Selection 13
3.2 Patient Eligibility Criteria 13
3.3 Clinic Stakeholder (Surgeons and Clinic Staff) Eligibility Criteria 14
3.4 Institutional Eligibility 14

4.0 PATIENT REGISTRATION 15

4.1 CTEP/DCP Registration Procedures 15
4.2 CTSU Registration Procedures 16
4.3 Patient and Clinic Stakeholder Registration Requirements 17
4.4 Institutional Randomization 19
4.5 Surgeon Registration 19
4.6 Patient Registration Procedures 19
4.7 Stratification Factors and Treatment Assignments 20

5.0 STUDY CALENDARS 21

5.1 Patient-level Study Calendar 21
5.2 Site Level Study Calendar 22

6.0 DATA SUBMISSION 23

6.1 Overview of Data Collection and Submission 23
6.2 Data Submission to the Alliance 25
6.3 Data Submission to the University of Wisconsin 25

7.0 STUDY INTERVENTION—INSTITUTIONS, SURGEONS, AND CLINIC STAFF 27

7.1 Clinic-Level Intervention 27
7.2 Individual-level Intervention—Surgeons and Clinic Staff 29
7.3 Incentives 30

8.0 STUDY INTERVENTION—PATIENT LEVEL 30

8.1 Screening 30
8.2 Patients enrolled before institutional crossover 31
8.3 Patients Enrolled following Institutional Crossover 32
8.4 Incentives 33

9.0 ADVERSE EVENTS 34

10.0 MEASURES 34

10.1 Definitions of Data for Primary Objective 34
10.2 Definitions of Data for Secondary Outcomes 39

11.0 END OF INTERVENTION/STUDY 42

11.1 Duration of Study 42
11.2 Criteria for Discontinuation of Study 42
11.3 Follow-up 42
11.4 Managing Ineligible Patients 42

12.0 STATISTICAL CONSIDERATIONS 43

12.1 Analysis Plan for Co-Primary Objectives 43
12.2 Analysis Plan for Secondary Objectives 50
12.3 Study Reporting 51
12.4 Inclusion of Women and Minorities 51

13.0 GENERAL REGULATORY CONSIDERATIONS: WAIVERS OF CONSENT 53

13.1 Waivers of documentation of written patient consent 53
13.2 Waiver of elements of informed consent—patients 55
13.3 Waivers of consent—surgeons and clinic staff 55
13.4 Waiver of elements of informed consent—surgeons 55
13.5 Other people present during audiotaped visits 55

14.0 REFERENCES 57

15.0 MODEL INFORMED CONSENT FOR PATIENTS 63

16.0 MODEL INFORMED CONSENT FOR SURGEONS 72

APPENDIX I: USUAL CARE – PATIENT BASELINE SURVEY 77

APPENDIX II: INTERVENTION – PATIENT BASELINE SURVEY 84

APPENDIX III: USUAL CARE – PATIENT FOLLOW-UP SURVEY 91

APPENDIX IV: INTERVENTION – ACCEPTED DA – PATIENT FOLLOW-UP SURVEY 102

APPENDIX V: INTERVENTION – DECLINED DA – PATIENT FOLLOW-UP SURVEY 114

APPENDIX VI: SURGEON BASELINE SURVEY 125

APPENDIX VII: PATIENT ACCESSIBILITY TELEPHONE INTERVIEW GUIDE 129

APPENDIX VIII: PATIENT PREPARATORY/INTERACTIONAL IN-PERSON INTERVIEW GUIDE 140

APPENDIX IX: PATIENT FOCUS GROUP GUIDE 146

APPENDIX X: CLINIC STAKEHOLDER FOCUS GROUP GUIDE 147

APPENDIX XI: SURVEY & INTERVIEW THANK YOU LETTER 148

APPENDIX XII: EMAIL BODY FOR PATIENT QUALTRICS SURVEY 149

1.0 BACKGROUND

1.1 Introduction

Shared decision making (SDM) may reduce health disparities by addressing barriers to patient engagement in decision making that disproportionately impact socioeconomically disadvantaged patients.[1,2] SDM interventions, e.g. decision aids, have the potential to increase patient involvement in decision making, improve health outcomes, and lower costs.[3] Decision aids may have the added benefit of reducing health disparities, since disadvantaged patients may benefit most from their use.[2] The goal of SDM is to impart knowledge while also empowering patients to engage in decision making. Decision aids increase knowledge.[2,4-6] However, barriers to patient empowerment hinder engagement, including lack of awareness about treatment choices, patients' perceptions that their personal input is not valued, and doctor-patient power imbalances.[1] There is limited understanding about how decision aids can overcome these barriers. This gap is significant as these barriers disproportionately affect disadvantaged patients.[1]

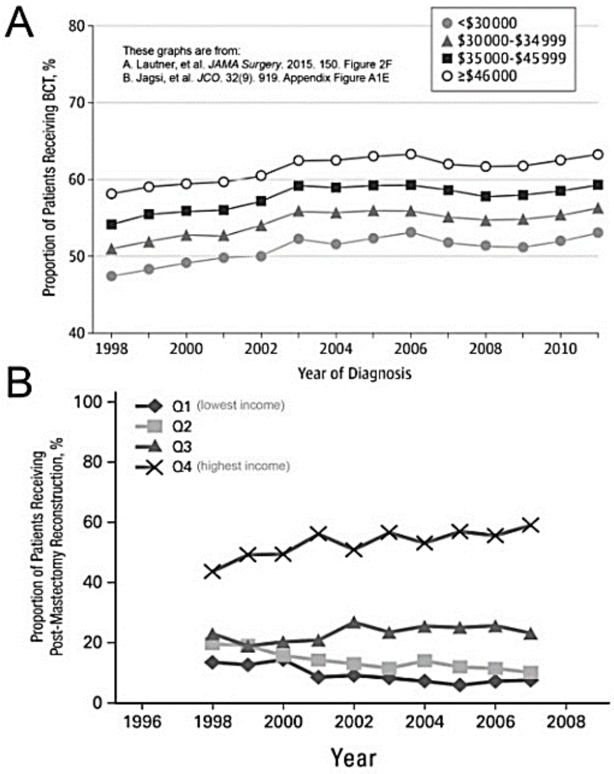

Figure 1. Proportion of socioeconomically disadvantaged patients receiving A, BCT and B, reconstruction.

In regards to breast cancer surgery specifically, multiple clinical trials demonstrate equivalent survival between breast conservation (BCT) and mastectomy (with or without reconstruction) for women diagnosed with early stage breast cancer.[7,8] However, patient-centered outcomes differ greatly, with a higher risk of local recurrence after BCT and a greater impact on body image with mastectomy. As most women are good candidates for both procedures, patients' values and preferences should drive decision making. Increased patient engagement in decision making may mitigate existing disparities in breast cancer surgical care.[9-15] Most population-based studies demonstrate that disadvantaged women are less likely to undergo BCT or receive post-mastectomy reconstruction (Figure 1).[16-20] This disparity is significant, as it can negatively impact long-term outcomes such as treatment regret, body image, and quality of life.[21-26] The etiology of these disparities is likely multifactorial.[17,18,27,28] However, patient engagement during the surgical consult is critical for this preference-sensitive choice.[9-15] Prior studies demonstrate that socioeconomically disadvantaged women have less understanding of treatment options,[29-31] are less likely to recall discussing a choice with their surgeon,[32,33] and participate less actively in decision making.[34] Breast cancer surgery decision aids have already been developed that could address these barriers, making breast cancer surgery an especially appropriate model for our work.

1.2 Conceptual Model

Our study is based on the theory described by Elwyn and colleagues that in order to engage in SDM, patients must have both (1) knowledge of the treatment options, and (2) power —the self-perceived need and capacity— to influence decision making (Figure 2).[1] Decision aids improve patients' knowledge about treatment options.[2,4-6] Decision aids may also prepare patients for the surgical consult by establishing expectations of their role during the consult, specifically that their input (i.e. their values and preferences) is welcome and essential. By preparing patients for surgical consultation, decision aids can increase patients' confidence in their ability to interact with their surgeon, empowering them to engage in SDM during the surgical consult. However, barriers to patient engagement may persist despite receipt of a decision aid and may decrease the effectiveness of a decision aid. These include accessibility barriers, preparatory barriers (e.g. patients feeling informed about treatment options, and being aware their input is necessary), and interactional barriers (e.g. perceiving a surgeon-patient power imbalance). Importantly, these persistent barriers may disproportionately impact socioeconomically disadvantaged patients.[1]

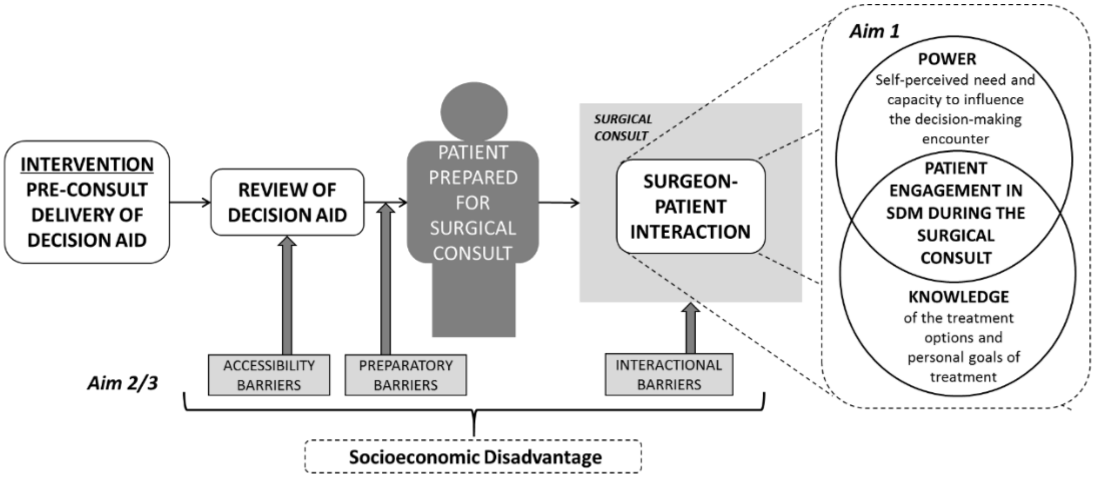

Figure 2. Our conceptual model is based on the theory that in order to engage in SDM, patients must have both the **power** to influence decision-making and **knowledge** about the options. Pre-consultation review of a decision aid can address these key conditions by preparing patients for the surgical consultation and increasing their confidence in interacting with the surgeon. However, barriers to engagement may limit the effect of the decision aid. These barriers disproportionately impact socioeconomically disadvantaged patients (adapted from Joseph-Williams, et al.[1]).

1.3 Gap Addressed in this Study

Our study is founded on the scientific premise that breast cancer surgery decision aids improve knowledge for most patients (necessary but insufficient alone to ensure engagement in SDM).[2,4-6,35,36] Based on our conceptual model, we posit that receipt of a decision aid prior to the surgical consult will also empower some patients, resulting in increased engagement in SDM. However, other patients will experience persistent preparatory or interactional barriers that cannot be addressed by the decision aid alone.

1.4 Study Overview

We propose a multi-site cluster randomized trial using a stepped wedge design to examine the impact of a decision aid on patient engagement in decision making, focusing on clinics that serve a high proportion of socioeconomically disadvantaged patients. We will assess patient engagement— measured by knowledge and power— after receipt of the decision aid through audio-recordings of the surgeon consultation and patient surveys. We will test the extent to which the effect of a decision aid on engagement is mediated through the mitigation of barriers, and determine if disadvantaged patients disproportionately experience persistent barriers. Finally, we will conduct interviews and focus groups to understand how persistent barriers

influence patient engagement, in order to identify targets for adjunct interventions. The objective of this study is to test the effectiveness of a decision aid in increasing patient engagement in SDM, and identify barriers to engagement not mitigated by the decision aid that could be targets for adjunct SDM interventions.

1.5 Design Considerations and Supporting Preliminary Data

1.5.1 Selection of a web-based platform for implementation

The potential benefits associated with decision aid use can only be realized if decision aids reach the "right patient at the right time".[37] Because of existing barriers to broad implementation and sustained use of decision aids, few patients ever receive one.[38,39] Online delivery, supported by theories in implementation science, communication, education, and psychology, effectively addresses structural barriers to decision aid use by enabling real-time identification of patients and delivery of the decision aid prior to the initial consult.[37,40-49] However, prior work demonstrates that it is insufficient to simply make a decision aid available online.[50] Instead, it is necessary to actively consider and address challenges to incorporating web-based decision aid delivery into routine clinic flow.[51] Although some concern exists that a web-based approach may selectively exclude at-risk patient populations, internet use has increased steadily over the past decade; those with less education and lower income have the fastest increases in adoption (Figure 3).[52,53] A critical component of this study is to evaluate accessibility barriers associated with a web-based decision aid.

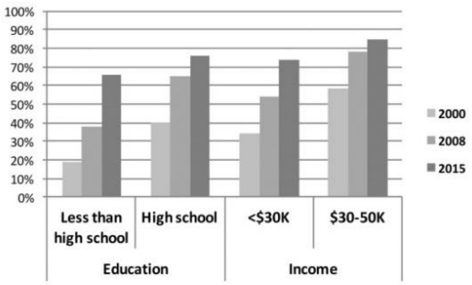

Figure 3. Use of Internet for American Adults. Adapted from Pew Research Center surveys, 2000-2015.

Preliminary data: Guided by the Replicating Effective Programs framework, we have developed a package of active implementation strategies that facilitates the incorporation of a web-based decision aid into routine care.[54,55] This strategy will guide implementation in this study. We elicited feedback from patients, nurses, and surgeons. These stakeholders endorsed the notion that delivery of a decision aid prior to the surgeon consult would best match patients' informational needs, and prepare them to participate actively in the consult; this is consistent with the literature.[56,57] Because our breast center (similar to other centers) strives for a narrow window between diagnosis and initial surgeon consult to minimize anxiety and delay, a web-based decision aid was the only feasible option. We also determined that email delivery was preferred, as many of our patients have not had the opportunity to establish a patient portal. Additional implementation strategies included identification of a surgeon-champion to elicit "buy-in" and performance of early audit-feedback.[54,58]

Using this strategy, we then delivered web-based information to 309 women in an academic and community clinic.[54] In this pilot, patients were randomized to receive either a web-based decision aid or a link to a standard website. Pilot participants were a median age of 59 years and 34% had less than a college degree. Most patients reviewed the websites (83%) and found this pre-consultation information highly beneficial (median score 8/10); results were similar for minority and socioeconomically disadvantaged patient subgroups.

We revised our implementation strategy in response to concerns from patients who declined pilot participation (n=68, 18% of patients approached). The most common reason was limited internet comfort or access (n=36). In response, we revised our implementation

strategy to include alternative ways patients could review web-based material, including emailing it to family members to review with patients and iPad access in the clinic. Several patients declined due to emotional distress (n=7) or preference to receive information directly from the surgeon (n=14). In response, we added scripts for use by clinic staff outlining the benefits of the decision aid in helping patients cope with their diagnosis, and including strong surgeon endorsement of the decision aid.

1.5.2 Use of Area Deprivation Index (ADI) to define socioeconomic disadvantage

Socioeconomic disadvantage describes the "state of being challenged by low income, limited education, and substandard living conditions for both the person and his or her neighborhood or social network".[59] A goal of this research is to reduce socioeconomic disparities in healthcare through the tailored delivery of targeted SDM interventions to those patients with the greatest decisional support needs. Operationalizing this approach requires early identification of these patients by clinic staff– without requiring a detailed patient assessment —to ensure ready integration into clinical workflow. In this study, we use the ADI. The ADI is an existing composite measure of neighborhood socioeconomic disadvantage, calculated based on an individual zip code+4.[59,60] It has been associated with cancer outcomes.[61-63] Zip+4-level ADI for the United States is publicly available from the PI institution.[64]

Preliminary data: We have successfully linked 99% of pilot study patients to the ADI, demonstrating feasibility in identifying vulnerable patients at the point of entry into the clinic. The five pilot patients who met criteria for socioeconomic disadvantage reviewed the decision aid and found it helpful (median score 9/10).

1.5.3 Use of a general-use decision aid

Because we are conducting this research in clinics that serve a high proportion of socioeconomically disadvantaged patients but that also care for patients in other socioeconomic strata, we are using a general-use decision aid. Using the same decision aid for the entire clinic population increases the feasibility and sustainability of the intervention. However, a critical part of the study is to evaluate the effectiveness of this decision aid for patients from different backgrounds.

The tool we selected was developed collaboratively by the Informed Medical Decisions Foundation and Health Dialog.[35, 55] Originally created as a DVD, it was converted to a web-based platform based on user feedback requesting flexibility in mode of delivery. The web-based decision aid utilizes static, didactic information written for an 8th grade reading level, and video clinical vignettes to promote consideration of personal values and preferences. It includes modules for invasive cancer, non-invasive cancer, and reconstruction. It has been used successfully in academic and community settings to increase knowledge and decrease decisional conflict.[35,43,44,48] The greatest gains in knowledge were seen in patients with lower educational backgrounds.[35]

Preliminary data: In our pilot study, we randomized patients to receive either the Health Dialog web-based decision aid, or links to standard websites, prior to the surgical consult; pre-consult knowledge was assessed. Knowledge was higher in patients who received the decision aid versus the standard websites (70% vs. 60%, p=0.004). These findings were unchanged after adjusting for sociodemographic characteristics.

1.5.4 Use of two measures for patient empowerment

Perceptions of a patient's role during a decision-making encounter depend on the method of measurement.[65] Because of this, we will assess our primary outcome of power by both (1) audio-recordings of the surgical consult, and (2) patient surveys.

Preliminary data: Our research team uses a dual approach of audio recordings and patient surveys to study decision making. We have experienced few barriers to patient recruitment for audio-recordings. Co-I Dr. Schwarze has successfully enrolled over 200 patients and audio-recorded pre-operative communications.[66-68] We have also conducted a pilot study within our breast center of audio-recording surgical consultations.

1.5.5 Focus on clinics representative of where most socioeconomically disadvantaged patients receive care.

We have chosen to enroll clinics that serve a high proportion of socioeconomically disadvantaged patients but also care for patients from other socioeconomic strata. Our overall goal is to reduce socioeconomic disparities in health care through shared decision making. Our preliminary data suggests that, nationwide, most socioeconomically disadvantaged patients receive their care in clinics that serve socioeconomically diverse patient populations. An approach to shared decision making that will work in these socioeconomically diverse clinics will be most generalizable, and will have the greatest chance to reduce disparities in cancer care.

Preliminary data: Using the National Cancer Database (2004-2013), we identified patients diagnosed with stage 0-3 breast cancer who were socioeconomically disadvantaged (median household income <$30,000, n =258,007). Patients received care at 1,282 facilities. We identified 61 facilities that cared for the greatest absolute number of disadvantaged patients (~25% of the cohort). At these facilities, disadvantaged patients comprised a median 30% of the total population. These data support our planned recruitment strategy.

1.6 Study Design

We plan a multi-site cluster randomized trial using a stepped wedge design with 7 waves. We will recruit 10 clinics for participation. All clinics will begin in the usual care arm. Clinics will be randomized to the timing with which they cross-over to the decision aid intervention, with new clinics crossing over at each wave starting from wave two. Maximizing reach of the decision aid within each clinic is critical for this intervention. We will use the strategy developed in our pilot work to guide implementation;[54] the process of implementation of the decision aid will occur over a 10-week period during which the clinic does not collect patient-level data. The research team will collect the clinic-level documents that support clinic's implementation efforts for research and take field notes during the implementation process. Data collection within each clinic before and after implementation of the decision aid intervention will be the same; this includes patient surveys and audio recording of the consult. The primary analysis will compare patients in usual care versus the decision aid intervention arms. We will follow the intervention component with patient interviews and focus groups as well as clinic stakeholder focus groups. In this component of the study, we will identify persistent barriers to patient engagement in decision making that could be targeted in future interventions.

We have experienced under-recruitment, in part due to the COVID-19 pandemic. To compensate for the slower accrual, we will extend the period of enrollment for sites that are still recruiting within the stepped wedge design as of 8/30/2021. The extended recruitment beyond Wave 7 would continue for six sites until 12/31/2021. Please see Section 12.1.3 for details.

2.0 OBJECTIVES

2.1 Co-primary objectives

Test the effectiveness of a breast cancer surgery decision aid in increasing patient engagement in decision making (measured by knowledge and power) in clinics serving a high proportion of socioeconomically disadvantaged patients

Test the extent to which the effect of a decision aid on patient engagement is mediated through the mitigation of barriers, and determine if persistent barriers are disproportionately experienced by socioeconomically disadvantaged patients

2.2 Secondary objective(s)

Characterize how persistent barriers influence patient engagement in decision making in order to identify targets for adjunct interventions that could be implemented in clinics serving a high proportion of socioeconomically disadvantaged patients

3.0 PARTICIPANT SELECTION

3.1 Patient Selection

For questions regarding eligibility criteria, see the Study Resources page. Please note that the Study Chair cannot grant waivers to eligibility requirements.

On-Study Guidelines

This clinical trial can fulfill its objectives only if patients appropriate for this trial are enrolled. All relevant medical and other considerations should be taken into account when deciding whether this protocol is appropriate for a particular patient.

Study staff should consider whether the following may render the patient inappropriate for this protocol:

- Psychiatric illness which would prevent the patient from giving informed consent.

3.2 Patient Eligibility Criteria

___ **3.2.1 Women newly diagnosed with clinical stage 0-III breast cancer.** Although men are recommended surgery to treat breast cancer, male breast cancer is relatively rare and decision making for breast cancer surgery is quite different between men and women.

In our pilot work, we have routinely included patients with clinical stage 0 cancer in the intervention. Although the prognosis differs between DCIS and invasive cancer, the core surgical decisions regarding mastectomy versus breast conservation remains the same.

___ **3.2.2 Eligible patients must be planning breast surgery as a component of their definitive treatment.**

___ **3.2.3 Patients with impaired decision-making capacity (such as with a diagnosis of dementia or memory loss) are not eligible for this study.**

___ **3.2.4 Patients with hearing impairment requiring the use of an interpreter are not eligible for this study.**

___ **3.2.5 Patients must be able to speak English** with the fluency required to have a direct discussion around treatment decision-making (i.e. without interpreter).

___ **3.2.6 Age ≥ 18 years**

3.3 Clinic Stakeholder (Surgeons and Clinic Staff) Eligibility Criteria

3.3.1 Breast surgeon(s) and nursing staff, medical assistant, or mid-level provider at each participating clinic who participates in the care of patients newly diagnosed with breast cancer.

3.4 Institutional Eligibility

The study will utilize the National Cancer Institute Oncology Research Program (NCORP).[19] Eligible clinics will serve a high proportion of socioeconomically disadvantaged patients, defined using two data sources. Figure 5 demonstrates the overlap between NCORP sites (represented as circles on the map) and U.S. neighborhoods designated as the most disadvantaged according to the ADI.[59] Using the 2015 NCORP Landscape Assessment, we have also identified 25 sites that report a high proportion of patients with Medicaid insurance (25-50%).

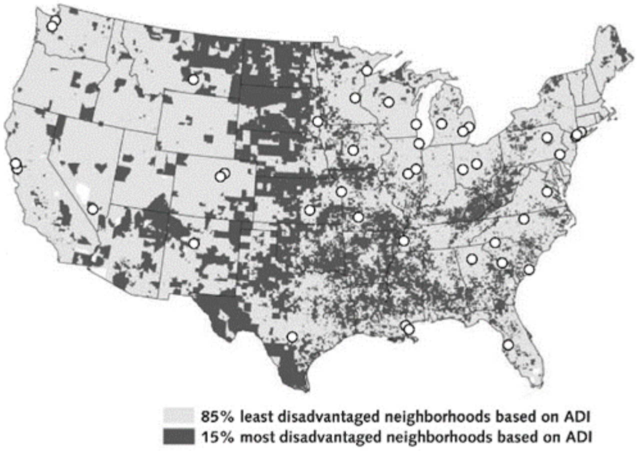

3.4.1 Using these data, 10 clinics that annually provide surgical care for 120-300 patients newly diagnosed with breast cancer will be selected to participate in this study.

3.4.2 Surgeons at eligible clinics must consent to the study as a requirement for site participation.

4.0 Patient Registration

4.1 CTEP/DCP Registration Procedures

Food and Drug Administration (FDA) regulations and National Cancer Institute (NCI) policy require all individuals contributing to NCI-sponsored trials to register and renew their registration annually. To register, all individuals must obtain a Cancer Therapy Evaluation Program (CTEP) Identity and Access Management (IAM) account at https://ctepcore.nci.nih.gov/iam. In addition, persons with a registration type of Investigator (IVR), Non-Physician Investigator (NPIVR), or Associate Plus (AP) must complete their annual registration using CTEP's web-based Registration and Credential Repository (RCR) at https://ctepcore.nci.nih.gov/rcr.

RCR utilizes five person registration types.

- IVR — MD, DO, or international equivalent;
- NPIVR — advanced practice providers (e.g., NP or PA) or graduate level researchers (e.g., PhD);
- AP — clinical site staff (e.g., RN or CRA) with data entry access to CTSU applications such as the Roster Update Management System [RUMS], OPEN, Rave, acting as a primary site contact, or with consenting privileges;
- Associate (A) — other clinical site staff involved in the conduct of NCI-sponsored trials; and
- Associate Basic (AB) — individuals (e.g., pharmaceutical company employees) with limited access to NCI-supported systems.

RCR requires the following registration documents:

Documentation Required	IVR	NPIVR	AP	A	AB
FDA Form 1572	✓	✓			
Financial Disclosure Form	✓	✓	✓		
NCI Biosketch (education, training, employment, license, and certification)	✓	✓	✓		
GCP training	✓	✓	✓		
Agent Shipment Form (if applicable)	✓				
CV (optional)	✓	✓	✓		

An active CTEP-IAM user account and appropriate RCR registration is required to access all CTEP and Cancer Trials Support Unit (CTSU) websites and applications. In addition, IVRs and NPIVRs must list all clinical practice sites and Institutional Review Boards (IRBs) covering their practice sites on the FDA Form 1572 in RCR to allow the following:

- Addition to a site roster;
- Assign the treating, credit, consenting, or drug shipment (IVR only) tasks in OPEN;
- Act as the site-protocol Principal Investigator (PI) on the IRB approval

Additional information is located on the CTEP website at https://ctep.cancer.gov/investigatorResources/default.htm. For questions, please contact the **RCR Help Desk** by email at RCRHelpDesk@nih.gov.

4.2 CTSU Registration Procedures

This study is supported by the NCI Cancer Trials Support Unit (CTSU).

IRB Approval

For CTEP and Division of Cancer Prevention (DCP) studies open to the National Clinical Trials Network (NCTN) and NCI Community Oncology Research Program (NCORP) Research Bases after March 1, 2019, all U.S.-based sites must be members of the NCI Central Institutional Review Board (NCI CIRB). In addition, U.S.-based sites must accept the NCI CIRB review to activate new studies at the site after March 1, 2019. Local IRB review will continue to be accepted for studies that are not reviewed by the CIRB, or if the study was previously open at the site under the local IRB. International sites should continue to submit Research Ethics Board (REB) approval to the CTSU Regulatory Office following country-specific regulations.

Sites participating with the NCI CIRB must submit the Study Specific Worksheet for Local Context (SSW) to the CIRB using IRBManager to indicate their intent to open the study locally. The NCI CIRB's approval of the SSW is automatically communicated to the CTSU Regulatory Office, but sites are required to contact the CTSU Regulatory Office at CTSURegPref@ctsu.coccg.org to establish site preferences for applying NCI CIRB approvals across their Signatory Network. Site preferences can be set at the network or protocol level. Questions about establishing site preferences can be addressed to the CTSU Regulatory Office by email or calling 1-888-651-CTSU (2878).

In addition, the Site-Protocol Principal Investigator (PI) (i.e. the investigator on the IRB/REB approval) must meet the following criteria in order for the processing of the IRB/REB approval record to be completed:

- Holds an active CTEP status;
- Rostered at the site on the IRB/REB approval (applies to US and Canadian sites only) and on at least one participating roster;
- If using NCI CIRB, rostered on the NCI CIRB Signatory record;
- Includes the IRB number of the IRB providing approval in the Form FDA 1572 in the RCR profile; and
- Holds the appropriate CTEP registration type for the protocol.

Additional Requirements

Additional requirements to obtain an approved site registration status include:

- An active Federal Wide Assurance (FWA) number;
- An active roster affiliation with the Lead Protocol Organization (LPO) or a Participating Organization (PO); and
- Compliance with all protocol-specific requirements (PSRs).

4.2.1 Downloading Site Registration Documents

Download the site registration forms from the protocol-specific page located on the CTSU members' website. Permission to view and download this protocol and its supporting documents is restricted based on person and site roster assignment. To participate, the institution and its associated investigators and staff must be associated with the LPO or a

Protocol Organization (PO) on the protocol. One way to search for a protocol is listed below.

- Log in to the CTSU members' website (https://www.ctsu.org) using your CTEP-IAM username and password;
- Click on *Protocols* in the upper left of the screen
    - Enter the protocol number in the search field at the top of the protocol tree; or
    - Click on the By Lead Organization folder to expand, then select *Alliance*, and protocol number *A231701CD*.
- Click on *Documents*, select *Site Registration*, and download and complete the forms provided. (Note: For sites under the CIRB, IRB data will load automatically to the CTSU.)

4.2.2 Submitting Regulatory Requirements

Submit required forms and documents to the CTSU Regulatory Office using the Regulatory Submission Portal on the CTSU website.

To access the Regulatory Submission Portal log in to the CTSU members' website, go to the Regulatory section and select Regulatory Submission.

Institutions with patients waiting that are unable to use the Regulatory Submission Portal should alert the CTSU Regulatory Office immediately at 1-866-651-2878 in order to receive further instruction and support.

4.2.3 Checking Site's Registration Status

Site registration status may be verified on the CTSU members' website.

- Click on Regulatory at the top of the screen;
- Click on Site Registration; and
- Enter the sites 5-character CTEP Institution Code and click on Go.
    - Additional filters are available to sort by Protocol, Registration Status, Protocol Status, and/or IRB Type.

Note: The status shown only reflects institutional compliance with site registration requirements as outlined within the protocol. It does not reflect compliance with protocol requirements for individuals participating on the protocol or the enrolling investigator's status with NCI or their affiliated networks.

4.2.4 Limited access information

This is a limited access study. All participating institutions will be selected by the study team per Section 3.4 prior to study activation.

4.3 Patient and Clinic Stakeholder Registration Requirements

- **Patient informed consent:** Patients must willingly consent after being informed of the study activities: alternatives, potential benefits, potential risks, and discomforts. Current human protection committee approval of this protocol and a consent form is required prior to patient consent and registration. Written consent is for two surveys and the audio recording of the surgical consult.

- **Surgeon informed consent:** Participating surgeons must willingly consent after being informed of the study activities: alternatives, potential benefits, potential risks, and discomforts. Current human protection committee approval of this protocol and a consent form is required prior to surgeon consent and registration. Written consent is for participation in site implementation activities, allowing their patients to receive the decision aid, baseline survey, and audio-recording of the surgical consult.
- **Waivers of consent:** For all patient interviews and patient and clinic stakeholder focus groups, we request a waiver of documentation of informed consent for participation given the low risk nature of the study activity. Participants in each of these activities will be provided with written information describing the research, their participation, the risks, possible benefits, and all other required elements of informed consent. See Section 13.0.
- **Patient- and surgeon-completed booklets:** Patient and surgeon questionnaire booklets are to be ordered prior to the registration of any patients or surgeons. Booklets can be ordered by downloading and completing the CTSU Supply Request Form, located on the CTSU Forms webpage under the Resources tab. The form must be submitted via the Regulatory Submission Portal on the CTSU website, located under the Regulatory tab. Samples of the booklets are found in Appendices I-VI, which are to be used for reference and IRB submission only. They are not to be used for patient completion.

Protected Health Information:

- Audio-recording of the consultation, interviews, and focus groups will be shared with the University of Wisconsin (UW). Although PHI will not be intentionally sent to UW, it is possible that PHI will be shared through the audio-recordings. All audio-recordings will be transcribed in full. Study staff members or outside contractors with a business associate agreement who have been approved by UW Legal Services/Privacy Officer for HIPAA compliance will be used for transcription. Although identifying information may exist on the audio files, transcriptionists will strip all identifying information once transcribed and will only have access to the subject ID that is assigned by study staff (no access to the patient-subject log or other identifying information). The de-identified transcripts will be used in data analysis by the research team. After data analysis is complete and manuscripts have been accepted for publication, the audio recordings will be destroyed and the de-identified transcripts will be stored indefinitely on a secure server.
- In order to facilitate completion of the Follow-up Survey, patients' email addresses will be entered into the Alliance database. This information will be used only by a web service to send the link for the follow-up survey. In addition, patients' zip +4 will be entered into the Alliance database (see Section 10.1.4.4).

4.4 Institutional Randomization

Prior to study activation, each of the 10 participating surgery clinics will be randomly assigned to a time when the clinic crosses over from usual care to intervention. To avoid anticipatory effects, clinics will be blinded to crossover time until the point of implementation. See Section 4.7 for a description of the stratification factors for this study.

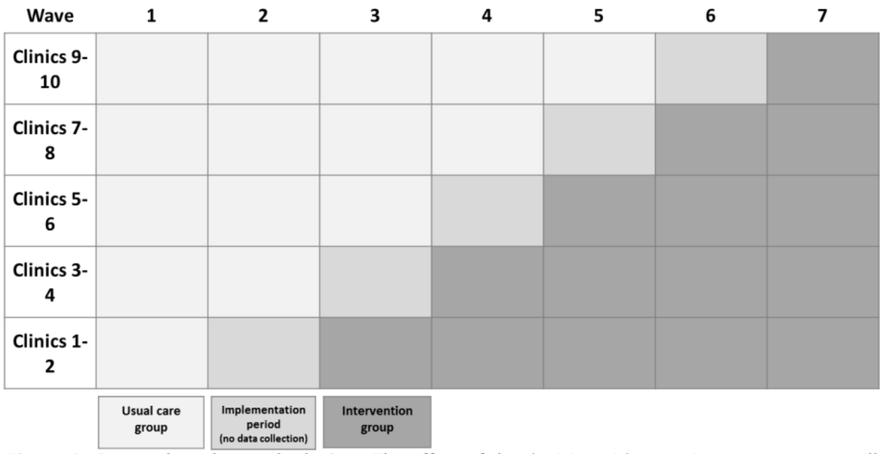

Figure 4. Stepped-wedge study design. The effect of the decision aid on patient engagement will be evaluated as a pre/post comparison through the stepped wedge design (years 1-2). Persistent barriers to engagement will be characterized through one-on-one patient interviews (years 2-4). Stakeholder focus groups will inform the identification of targets for adjunct interventions (years 4-5).

4.5 Surgeon Registration

Surgeons will be identified during the site selection process. Following local IRB approval of this study, surgeons will be consented to participate in site implementation activities, baseline survey, and audio recording of the surgical consult.

The Surgeon Enrollment Form should then be completed and submitted to Alliance Registration Office via fax (507-284-0885) or email (random01@mayo.edu). The registration office will register the surgeon(s) to the study and send confirmation to the site via email within 1 business day. (Note: Surgeon(s) will **not** be registered to this study using OPEN.)

4.6 Patient Registration Procedures

The Oncology Patient Enrollment Network (OPEN) is a web-based registration system available on a 24/7 basis. OPEN is integrated with CTSU regulatory and roster data and with the LPOs registration/randomization systems or the Theradex Interactive Web Response System (IWRS) for retrieval of patient registration/randomization assignment. OPEN will populate the patient enrollment data in NCI’s clinical data management system, Medidata Rave.

Requirements for OPEN access:

- A valid CTEP-IAM account;
- To perform enrollments or request slot reservations: Must be on an LPO roster, ETCTN corresponding roster, or participating organization roster with the role of Registrar. Registrars must hold a minimum of an Associate Plus (AP) registration type; and
- Have an approved site registration for the protocol prior to patient enrollment.

To assign an Investigator (IVR) or Non-Physician Investigator (NPIVR) as the treating, crediting, consenting, drug shipment (IVR only), or receiving investigator for a patient transfer in OPEN, the IVR or NPIVR must list the IRB number used on the site’s IRB approval on their

Form FDA 1572 in RCR. If a DTL is required for the study, the IVR or NPIVR must be assigned the appropriate OPEN-related tasks on the DTL.

Prior to accessing OPEN, site staff should verify the following:

- Patient has met all eligibility criteria within the protocol stated timeframes; and
- All patients have signed an appropriate consent form and HIPAA authorization form (if applicable).

Note: The OPEN system will provide the site with a printable confirmation of registration and treatment information. You may print this confirmation for your records.

Access OPEN at https://open.ctsu.org or from the OPEN link on the CTSU members' website. Further instructional information is in the OPEN section of the CTSU website at https://www.ctsu.org or https://open.ctsu.org. For any additional questions, contact the CTSU Help Desk at 1-888-823-5923 or ctsucontact@westat.com.

4.7 Stratification Factors and Treatment Assignments

4.7.1 Stratification

The randomization routine is found in Section 12.0 (Statistical Considerations). Randomization occurs at the clinic practice site level only. Patient level stratification is not considered in this study.

There is one planned composite stratification factor with two levels (group 1; group 2). When assigning sites to a group, the primary factor considered was whether the site was a minority/underserved NCORP site. Sites will be stratified as follows:

Group 1: Wisconsin NCORP, Montana Cancer Consortium NCORP, Bay Area Tumor Institute NCORP, Carle Cancer Center NCORP, Hawaii M/U NCORP

Group 2: University of New Mexico M/U NCORP, Medical University of South Carolina M/U NCORP, Virginia Commonwealth University Massey Cancer Center M/U NCORP, Stroger Hospital of Cook County M/U NCORP, Georgia Cares M/U NCORP

4.7.2 Grouping Factors

The grouping factors are as follows: a.) Standard of care, No decision aid, and b.) Standard of care, With decision aid

5.0 STUDY CALENDARS

5.1 Patient-level Study Calendar

	Prior to Registration	Surgical Consultation†	Post-Consultation	Post-Surgery
	Prior to Institutional Crossover			
Baseline Survey*		X		
Audio Recording		X		
Follow up Survey**			X	
Chart Review				X
	Beginning 10 Weeks after Institutional Crossover*			
Decision Aid email	X			
Demographics and 'Reason(s) Why'	X (3)			
Baseline Survey*		X		
Audio Recording		X		
Follow up Survey**			X	
Chart Review				X
In-Person, Telephone, or Video Interviews (Accessibility Barriers)				X (1)
In-Person, Telephone or Video Interviews (Preparatory/Interactional Barriers)				X (2)
Focus Group				X (2)

† Registration can occur before or after surgical consultation, as long as patient is consented prior to any surveys and/or audio-recordings take place.

* Booklet; see Appendix I or II.

** Web-Based or Booklet: see Appendix III, IV, or V. The follow-up survey must be completed after the patient leaves the surgeon's office.

*** After institutional crossover, patients may participate in the surveys and audio-recordings regardless of whether they were offered the decision aid, or whether they did or did not accept it. Accepting or not accepting the decision aid only influences opportunity for participation in patient interviews and focus groups.

1) Subset of patients who either declined decision aid when offered (from clinic log), accepted it but had difficulty accessing it (baseline survey), or accepted it but did not use it after receiving it (baseline survey).
2) Subset of patients who used the decision aid.
3) Patients who decline the decision aid

5.2 Site Level Study Calendar

	Prior to 1st Patient	Usual Care	Institutional Crossover Implementation (10 Weeks)	Crossover Complete
Surgeon and Clinic Staff recruitment	S, CS (2)			
Baseline surgeon survey *	S (2)			
Site collects patient-level data		(2)		(2)
Audio recordings during consultations		S (2)		S (2)
Implementation package:				
Clinic Self-Assessment Worksheet			S, CS (1)	
Field notes			S, CS (1)	
Audit-Feedback			S, CS (1, 3)	
Measure reach of Decision Aid				CS (2)
Clinic characteristics				CS (2)
Clinic focus groups				S, CS (1)

S-Surgeons CS-Clinic Staff

* Booklet; see Appendix VI.

1) Administered/collected by University of Wisconsin research team.

2) Administered/collected by site research team.

3) Audit-Feedback will occur at about 3 and 6 weeks following the crossover date

6.0 DATA SUBMISSION

6.1 Overview of Data Collection and Submission

All data are to be submitted within one week of collection.

The following data will be collected by each site's research coordinator:

- Surgeon baseline survey
- Patient pre-consultation baseline survey
- Audio file of surgical consult
- Patient follow-up survey
- Patient chart review – abstracting clinical data points
- Patient telephone, video, or in-person interviews for accessibility barriers (subset of patients who either declined decision aid when offered, accepted it but had difficulty accessing it, or accepted it but did not use it after receiving it, audio-files)
- Reach after change to intervention arm
- Clinic working documents associated with implementation activities (clinic implementation worksheet, audit-feedback documentation)

The following data will be collected by the UW research team:

- Field notes from observations of clinic implementation activities
- Patient telephone, video or in-person interviews for preparatory/interactional barriers (subset of patients who used the decision aid and participated in the surveys/audio recordings, audio-files)
- Patient focus groups (subset of patients who used the decision aid and participated in the surveys/audio recording, audio-files)
- Clinic stakeholder focus groups (subset of stakeholders, audio-files)

Data collection type	Who Collecting	Where to submit
Surgeon baseline survey responses	Site coordinator	Enter into iMedidata/Rave
Patient baseline survey responses	Site coordinator	Enter into iMedidata/Rave
Audio files of surgical consult	Site coordinator	Upload and send through UW SecureBox site or secure file transfer service
Follow-up post-consultation survey responses	Site coordinator	Enter into iMedidata/Rave
Patient chart review – abstracting clinical data points	Site coordinator	Enter into iMedidata/Rave
De-identified copies of clinic implementation worksheet	Site coordinator	Upload and send through UW SecureBox site or secure file transfer service
Field notes of implementation efforts	UW research team	Upload by research team to UW SecureBox site or secure file transfer service
Audio files of qualitative telephone, video, or in-person interviews (subset of patients)	Site coordinator	Upload and send through UW SecureBox site or secure file transfer service
Reach	Site coordinator	Upload and send through UW SecureBox site or secure file transfer service
Audio files of one-on-one telephone, video or in person interviews (subset of patients)	UW research team	Upload by research team to UW SecureBox site or secure file transfer service
Patient and Stakeholder focus groups	UW research team	Upload by research team to UW SecureBox site or secure file transfer service

Patient Screening Log (de-identified)	Site coordinator	Upload and send through UW SecureBox site or secure file transfer service
List of patients who decline decision aid with limited demographics (de-identified)	Site clinic staff	Upload and send through UW SecureBox site or secure file transfer service

6.1.1 Data Quality Portal

The Data Quality Portal (DQP) provides a central location for site staff to manage unanswered queries and form delinquencies, monitor data quality and timeliness, generate reports, and review metrics.

The DQP is located on the CTSU members' website under Data Management. The Rave Home section displays a table providing summary counts of Total Delinquencies and Total Queries. DQP Queries, DQP Delinquent Forms and the DQP Reports modules are available to access details and reports of unanswered queries, delinquent forms, and timeliness reports. Review the DQP modules on a regular basis to manage specified queries and delinquent forms.

The DQP is accessible by site staff that are rostered to a site and have access to the CTSU website. Staff that have Rave study access can access the Rave study data using a direct link on the DQP.

To learn more about DQP use and access, click on the Help icon displayed on the Rave Home, DQP Queries, and DQP Delinquent Forms modules.

Note: Some Rave protocols may not have delinquent form details or reports specified on the DQP. A protocol must have the Calendar functionality implemented in Rave by the Lead Protocol Organization for delinquent form details and reports to be available on the DQP. Site staff should contact the LPO Data Manager for their protocol regarding questions about Rave Calendaring functionality.

6.2 Data Submission to the Alliance

Medidata Rave is a clinical data management system being used for data collection for this trial/study. Access to the trial in Rave is controlled through the CTEP-IAM system and role assignments.

Requirements to access Rave via iMedidata:

- A valid CTEP-IAM account; and
- Assigned a Rave role on the LPO or PO roster at the enrolling site of: Rave CRA, Rave Read Only, Rave CRA (LabAdmin), Rave SLA, or Rave Investigator.

Rave role requirements:

  - Rave CRA or Rave CRA (Lab Admin) role must have a minimum of an Associate Plus (AP) registration type;
  - Rave Investigator role must be registered as an Non-Physician Investigator (NPIVR) or Investigator (IVR); and
  - Rave Read Only role must have at a minimum an Associates (A) registration type.

Refer to https://ctep.cancer.gov/investigatorResources/default.htm for registration types and documentation required.

Upon initial site registration approval for the study in Regulatory Support System (RSS), all persons with Rave roles assigned on the appropriate roster will be sent a study invitation e-mail from iMedidata. To accept the invitation, site staff must log in to the Select Login (https://login.imedidata.com/selectlogin) using their CTEP-IAM username and password and click on the *accept* link in the upper right-corner of the iMedidata page. Site staff will not be able to access the study in Rave until all required Medidata and study specific trainings are completed. Trainings will be in the form of electronic learnings (eLearnings) and can be accessed by clicking on the link in the upper right pane of the iMedidata screen. If an eLearning is required and has not yet been taken, the link to the eLearning will appear under the study name in iMedidata instead of the *Rave EDC* link; once the successful completion of the eLearning has been recorded, access to the study in Rave will be granted, and a *Rave EDC* link will display under the study name.

Site staff that have not previously activated their iMedidata/Rave account at the time of initial site registration approval for the study in RSS will receive a separate invitation from iMedidata to activate their account. Account activation instructions are located on the CTSU website in the Data Management section under the Rave resource materials (Medidata Account Activation and Study Invitation Acceptance). Additional information on iMedidata/Rave is available on the CTSU members' website in the Data Management > Rave section at www.ctsu.org/RAVE/ or by contacting the CTSU Help Desk at 1-888-823-5923 or by e-mail at ctsucontact@westat.com.

6.3 Data Submission to the University of Wisconsin

All data transferred to UW will be sent using the UW SecureBox site or Surgery and Urology File Transfer system.

Following audio-file data collection from either a T1 surgical consult or patient interview, site research coordinators will send the audio-file of the conversation to UW for transcription. Site coordinators will ensure that the audio-file's title includes the patient's study ID number (no identifiers). Due to data security concerns, audio-files will only be shared using either the UW SecureBox site or Surgery and Urology File Transfer system. They are both a secure, password-

protected system. These audio-files will be downloaded to the secure UW Department of Surgery server. Once UW receives the file and reviews it, they will give instructions to the site research coordinator to delete their copy of the audio file.

The audio-files will then be transcribed in their entirety. Although identifying information may exist on the audio files, transcriptionists will strip all identifying information once transcribed and will only have access to the subject ID that is assigned by study staff (no access to the patient-subject log or other identifying information). Study staff members or outside contractors with a business associate agreement who have been approved by UW Legal Services/Privacy Officer for HIPAA compliance will be used for transcription. After data analysis is complete and manuscripts have been accepted for publication, the audio recordings will be destroyed and the de-identified transcripts will be stored indefinitely on a secure server.

7.0 Study Intervention—Institutions, Surgeons, and Clinic Staff

7.1 Clinic-Level Intervention

7.1.1 Prior to Institutional Crossover—Usual Care

After study activation, sites will continue to provide breast cancer surgical care per their usual care. Site research coordinators will audio-record surgeon's visit conversation with enrolled patients.

7.1.2 Institutional Crossover to Intervention (Decision Aid)

Each site will start in the usual care arm. At the start of each wave, starting from wave two, two sites will crossover into the intervention arm (see Section 4.4 for additional details). Each site will be notified four weeks prior to their crossover date.

After the date of crossover to the intervention, all patients within the clinic will be asked whether they would like to receive the decision aid by email as accepted practice in the clinic. This design, where receipt of the decision aid is not linked to study consent, maximizes reach of the decision aid intervention itself, especially important when working with disadvantaged patient populations that are less likely to participate in research.69

Staff will offer the decision aid when a surgery consult is scheduled and maintain a log of all patients who are offered the decision aid. If a patient expresses interest in receiving the decision aid, clinic staff will obtain and record their preferred email address, and they will send the patient the decision aid. Because of privacy concerns, the email body does not include the word "cancer". The email will be sent by a staff member to an email address supplied by the patient themselves.

Patients who report difficulty with internet access will be offered two options: (1) have the decision aid sent to a family member, and (2) view the decision aid in clinic on an iPad. Although the decision aid is currently only available in English, we will offer it to all patients regardless of primary language, assuming patients may identify ways it could be beneficial (e.g. review with English speaking family member). For patients with visual impairment, the decision aid will be offered using an alternative strategy, such as (but not limited to) emailing it to family members to review.

7.1.3 Ten-week Implementation of Decision Aid

Starting on the date of crossover, there will be a 10-week implementation period when patient-level data are not collected in order to allow the decision aid to be fully implemented at each clinic. The UW research team will provide clinic management with the implementation package. Each site will have a designated surgeon champion who will help facilitate implementation; this individual will have been identified at the time of site selection for the study and is not necessarily the site study PI. The primary steps in implementation include creating an implementation change team at each clinic comprised of key clinic stakeholders, completion of a clinic self-assessment worksheet that includes process mapping of patients' flow through clinic, and brief audit-feedback loops. These data from the clinic self-assessment worksheet and the audit-feedback, along with field notes from any interactions between the UW research team and the clinic, will be kept as data regarding the implementation process.

- **Clinic self-assessment worksheet** is a self-assessment worksheet that guides the team through consideration of perceived barriers and facilitators to implementing the decision aid, and through process mapping of patients' flow through clinic to ensure all eligible patients will be offered the decision aid.[70,71]

- **Field notes** of all interactions between the UW research team and the clinic will be kept. Trained members of the UW research team will directly observe the meeting where the clinic self-assessment worksheet is completed; the UW research team will take structured field notes. We will explicitly record observations about the clinic informational technology infrastructure which may facilitate or hinder implementation. In addition to observing the processes by which solutions to barriers are identified, other dimensions relevant to implementation such as team leadership by the surgeon champion and conflict amongst the team will be recorded in the field notes. Following the assessment, each site research coordinator will code the completed clinic self-assessment worksheets to remove all patient and team member names. The coded dataset (without identifiers) will be shared with the UW research team via the secure UW SecureBox or Surgery & Urology File Transfer service.
- **Audit-Feedback** will occur at about 3 and 6 weeks following the crossover date as a tool to improve the implementation of the decision aid. During the 10-week implementation phase, no patient-level data are collected. The UW and site research team will work together to calculate the proportion of eligible patients who are sent the decision aid out of the total eligible during the 3-week period and who access the decision aid. The UW research team will support the clinic team in identifying the best method for collecting data on reach (e.g. using billing data or direct review of clinic schedules).[58,72,73] The data in the audit feedback tool will be coded by the site research coordinator to remove all patient and team member names. The coded (without identifiers) dataset will be provided to the UW research team. The UW research team and the site will work together to identify opportunities to increase the reach of the decision aid in their clinic.

Although the materials from implementation are part of the implementation process, they are also considered research materials, as they will be used to enhance the overall intervention. Participants will not be intentionally asked any questions that pertain to private health information about specific clinic staff or patients during these sessions. However, it is a possibility that a participant could choose to answer an open-ended question with identifiable private information. For this reason, all items obtained during the sessions will be coded.

7.1.4 Following the 10-week implementation period

Clinic staff will continue to offer the decision aid when a surgery consult is scheduled (as described in the preceding section) and maintain a log of all patients who are offered the decision aid, including who accepts, who declines and reason(s) why. This data is collected as part of standard of care to guide ongoing implementation activities in order to maximize clinic-wide use of the decision aid.

Similar to the usual care period, during the intervention period, site research coordinators will audio-record surgeon's conversation with enrolled patients.

For all patients who decline the decision aid, site coordinators will collect limited additional information about them (medical record number, age, race, ethnicity, insurance status, ZIP code + 4, and reason(s) why declined) to ensure we are not systematically excluding certain populations from our study.

At the end of the study period, the site coordinators will collect the total number of patients who were offered the decision aid, the total number of patients who were sent the decision aid, and the total number of patients seen in the clinic during the

intervention arm window. These numbers will be uploaded to UW to calculate reach of the intervention.

7.2 Individual-level Intervention—Surgeons and Clinic Staff

7.2.1 Surgeons

Recruitment: Lists of surgical personnel will be used to invite surgeons to participate in the study. Prior to patient enrollment, all surgeons will be contacted by the site Principal Investigator or designee regarding participation. Surgeons will be provided information about the study and asked if s/he would be interested in meeting in-person with the site's research coordinator to discuss the study.

Consent: Surgeon consent is for participation in site implementation activities, allowing their patients to receive the decision aid, a baseline survey, and audio-recording of their patient consultations. Surgeons will have an in-person meeting with study staff to explain the components of the study (including risks, etc.), inquire of whether the surgeon would like to participate, and study staff will obtain written informed consent in a private and quiet room.

Surgeon Baseline Survey: Prior to beginning patient enrollment, the site research coordinator will administer baseline survey to all consenting surgeons at the site. The survey booklets should take about 10 minutes to complete. If the survey is not completed, the site research coordinator will send reminders, either by email or by telephone calls. Surgeons will be contacted up to 6 times to complete the survey.

Focus Groups: Clinic stakeholder focus group will take place at a national meeting during years 4/5. We will poll possible focus group participants to identify the national meeting with the greatest planned attendance; examples could include the Alliance for Clinical Trials in Oncology biannual meeting, the American College of Surgeons annual meeting, or American Society of Breast Surgeons annual meeting. We will plan to hold our focus group meetings at this meeting. We will additionally have an on-line option for surgeon stakeholders not attend the meeting as well as other clinic stakeholders less likely to attend these national meetings. We will conduct one focus group at the beginning and one near the end of the meeting; this will allow time to review the focus group and revise the focus group script in order to explore critical themes. We will invite surgeons from each clinic to participate in the focus groups.

7.2.2 Clinic Staff

Recruitment: The site Principal Investigator and site research coordinator will use staff lists to identify clinic staff. Prior to implementation and patient enrollment, all clinic staff will be contacted about the study and their anticipated role. The recruitment materials will include information about the implementation activities and focus groups. In addition, the email will ask if they would be interested in meeting in-person with study staff to discuss the study. Clinic staff members have the possibility to opt out of meeting in-person to learn more about the study by responding to the email stating they are not interested. Clinic stakeholders will be contacted a maximum of three times to gauge interest in the study.

All clinic staff will be eligible to participate in the implementation activates (clinic self-assessment working session) at their site.

Focus Groups: Clinic stakeholder focus group will take place at the Alliance for Clinical Trials in Oncology biannual meeting during years 4/5. We will conduct one focus group at the beginning and one near the end of the meeting; this will allow time to review the focus

group and revise the focus group script in order to explore critical themes. Alternatively, due to the impact of the novel coronavirus (COVID-19) surgeon and clinic staff focus groups may occur virtually via telephone or video. Select staff will be invited to participate in the final focus group after discussions with the surgeon champion at each clinic and the clinic manager based on their level of involvement in the decision aid implementation and their contact with patients.

7.3 Incentives

7.3.1 Surgeons

Surgeons will not receive compensation for their participation in the implementation activities, baseline survey and audio recordings of their surgical consultations.

Surgeons will be provided an incentive of $100 for clinic stakeholder focus group participation. The UW research team would provide the incentive directly to these participants at completion of the focus group or mail the incentive, depending on whether the participant attends the focus group in-person or not.

7.3.2 Clinic Staff

Staff will not receive compensation for their participation in the implementation activities.

Staff will be provided an incentive of $100 for clinic stakeholder focus group participation. The UW research team would provide the incentive directly to participants at completion of the focus group or mail the incentive, depending on whether the participant attends the focus group in-person or not.

8.0 STUDY INTERVENTION—PATIENT LEVEL

8.1 Screening

Patient eligibility in research begins at the time of their first surgical consultation, regardless of whether they are in the usual care or decision aid intervention arm and whether they did or did not accept the decision aid. Each week, surgeons, clinical personnel, and the site's research coordinator, will pre-screen patients coming to see clinic surgeons who may meet eligibility criteria. Screening will occur through contact with service teams as well as through medical chart review. In order to conduct these pre-screenings, the research team will obtain a waiver of consent as it will be impractical to obtain consent to screen for all eligible patients.

Site coordinators will be asked to distribute their recruitment to all of the participating surgeons practicing at that clinic. In general, enrollment should be proportional to the volume of breast cancer patients each surgeon sees. Site coordinators will be provided with target enrollment goals for each participating surgeon in the clinic prior to the start of the study.

Our goal for this study is to enroll ~30% socioeconomic disadvantaged patients; to accomplish this, we have selected clinics enriched for this population. We will also provide site coordinators with explicit training regarding enrollment of patients from different socioeconomic strata. In this study, we are defining socioeconomic disadvantage based on the Area Deprivation Index, which is determined by the zip code+4. To help site coordinators enroll patients from each socioeconomic strata, we will provide each site coordinator with a list of zip code+4 that represent socioeconomic disadvantage in their clinic catchment area for reference. We will provide monthly reports to clinics so that they can track their progress. We also plan a Lead-in Period prior to wave 1, to ensure clinics can successfully enroll patients and make any necessary upfront changes to site coordinators recruitment strategies.

8.2 Patients enrolled before institutional crossover

8.2.1 Consent process: Patients who meet eligibility criteria and are identified as potential participants by the site research coordinator for the primary objective will be queried by clinic staff during the rooming process regarding study participation. If the patient expresses interest in participation, appropriate site personnel will meet with the patient prior to her visit with the surgeon to explain the study; receive informed consent**,** (for the audio recording of the consultation, two surveys, and contacting for future participation in either an interview or focus group); and register the patient.

If patients decline to participate in a research activity, the research coordinator will record age, race, ethnicity, insurance, and reason for declining. These data points will be collected so that we may assess whether participants (both in the usual care and intervention group) are categorically different than non-participants. This data is completely de-identified. As these patients have declined research, they will not be approached for future research activities. This decline applies only to that specific research activity and any subsequent research activities. The decline does not apply to any prior data collection unless specified by the patient.

Some sites may mail or email a letter introducing the study to patients in advance of their arrival to clinic. Site personnel may download the Patient Introduction Email or Letter from the Supplemental Materials tab on the Alliance and CTSU websites. This would likely be for logistical reasons and planning of clinic flow. This letter would invite patients to contact the site research coordinator in advance of the clinic appointment to learn more about the study. This letter would allow contact with the patient prior to their presentation in clinic. In addition, the letter also provides an opportunity for patients to opt out of the study altogether.

8.2.2 Surgical consultation: After patient consent, the site research coordinator will administer the baseline survey to the patient. The preference is for the survey to be administered orally, although it may be completed on a paper booklet as well. The surgeon consultation will be audio-recorded. The site research coordinator will turn on the audio-recorder prior to leaving the room and the audio-recorder will be collected following the consultation.

Individuals other than the patient and attending surgeon may be present during the audiotaped clinic visit. This includes family members, residents, nurses and medical students. Although they are not study participants, they will receive written notification that the visit will be or is currently being recorded. We will allow individuals who do not verbally agree to be audiotaped to step out of the room during the time the audio recorder is on. This approach has been previously used with patients considering high risk operations and it has been tolerated well by their companions and other clinic staff. However, if individuals who do not verbally agree to be audiotaped do not wish to leave the room, we will not audio record the visit, out of sensitivity to the patient's needs.

8.2.3 Patient Surveys: Immediately after the surgical consult, the site research coordinator will send the patient an email inviting them to participate in the post-consultation follow-up survey. Note: the follow-up survey must be completed after the patient leaves the surgeon’s office.

Patients will be sent a link for a survey using a secure Qualtrics survey site and they will be reminded not to include any identifying information in their survey responses. The survey should take about 10 minutes to complete. If no response is received, the site research coordinator will send reminder emails, followed by telephone calls. An additional strategy will be for the site research coordinator to approach patients at a follow-up visit to

complete the survey either orally or on paper. If the patient does not have an email address or if she prefers she may complete the survey orally or on a paper booklet, they will be asked whether they would like to complete the survey with a paper booklet or by telephone. Patients will be contacted up to 6 times to complete the survey. The goal is to complete the survey within 3 weeks of the surgeon consult.

8.3 Patients Enrolled following Institutional Crossover

The only difference between patients enrolled before institutional crossover and patients enrolled after institutional crossover is those who are enrolled after institutional crossover will have been offered the decision aid as accepted practice in the clinic. Patients will be approached for participation in the surveys and audio-recording regardless of whether or not they accepted the decision aid.

The consent process, surgical consultation, and patient surveys for these patients will be identical to those described above.

8.3.1 Decision aid: The decision aid used in this study was developed collaboratively by the Informed Medical Decisions Foundation and Health Dialog.[35,55] Originally created as a DVD, it was converted to a web-based platform based on user feedback requesting flexibility in mode of delivery. The web-based decision aid utilizes static, didactic information written for an 8th grade reading level, and video clinical vignettes to promote consideration of personal values and preferences. It includes modules for invasive cancer, non-invasive cancer, and reconstruction. It has been used successfully in academic and community settings to increase knowledge and decrease decisional conflict.[35,43,44,48] The greatest gains in knowledge were seen in patients with lower educational backgrounds.[35]

8.3.2 Interviews and Focus Groups: After the clinic crosses over to the decision aid arm, clinic staff will track patients who decline the decision aid.

Patients seen in clinics that have crossed over to use of the decision aid may be eligible for participation in interviews and focus groups addressing the secondary objectives.

Patients who did not accept the decision aid:

Patients will be eligible to participate in **telephone, video, or in-person interviews** addressing Accessibility Barriers if they declined the decision aid (from clinic log). We will purposefully sample patients who experienced access barriers (identified through survey responses). If we find there is a strong association between accessibility barriers and socioeconomic disadvantage, or that accessibility barriers cluster within certain clinics, we will oversample based on those factors.

If patients decline to participate the interview, the research coordinator will record age, race, ethnicity, insurance, and reason for declining. These data points will be collected so that we may assess whether participants are categorically different than non-participants. This data is completely de-identified. As these patients have declined research, they will not be approached for future research activities. This decline applies only to that specific research activity and any subsequent research activities. The decline does not apply to any prior data collection unless specified by the patient.

Patients who did accept the decision aid:

Patients will be eligible to participate in **telephone, video, or in-person interviews** addressing Accessibility Barriers if they reported difficulty accessing the decision aid (from baseline survey) or did not use the decision aid (from baseline survey). We will purposefully sample patients who experienced access barriers (identified through survey responses). If we find there is a strong association between accessibility barriers and

socioeconomic disadvantage, or that accessibility barriers cluster within certain clinics, we will oversample based on those factors.

Patients will be eligible to participate in a **telephone, video or in-person interview** addressing Preparatory/Interactional Barriers if they accepted the decision aid. We will focus our sampling on patients with low engagement in decision-making, defined as the lowest tertile for either the PEPPI score or the Street Activation measure. We will purposefully sample patients with low engagement who experienced one or more of the preparatory or interactional barriers outlined in the primary objective (quantitative data collection). We will use other relevant data such as patient age, race, and ADI to ensure a range of participants. We will consider surgeon and clinical site to minimize clustering of interview participants at the provider or site level.

Patients will also be eligible to participate in a **focus group** if they accepted the decision aid. We plan to conduct three patient focus groups at three separate clinics. Again, **focus groups may occur virtually via telephone or video**, if in-person interactions are not possible due to COVID-19 or at the preference of the site. We will select clinics with a relatively high prevalence of patients experiencing the barriers on our list of potential targets (from quantitative data collection). The specific characteristics of patient participants within each clinic will be determined from the quantitative analyses that comprise the primary objective.

If patients decline to participate the interviews or focus groups, the research coordinator will record age, race, ethnicity, insurance, and reason for declining. These data points will be collected so that we may assess whether participants are categorically different than non-participants. This data is completely de-identified. As these patients have declined research, they will not be approached for future research activities. This decline applies only to that specific research activity and any subsequent research activities. The decline does not apply to any prior data collection unless specified by the patient.

8.4 Incentives

We will provide patients $10 for the audio recording and baseline survey. We will provide an additional $10 to patients who complete the post-consultation follow-up survey. We will provide patients an additional incentive of $20 for interview participation. Lastly, we will provide patients an additional incentive of $20 for focus group participation.

Incentives will be delivered to patients through a variety of means, based on the specific research activity. The UW research team will provide site coordinators with gift cards on a quarterly basis. Site coordinators and the UW research team will log the patient study ID and gift card number for tracking purposes.

Patient Research Activity	Amount	Incentive delivery
Baseline survey and audio-record consult	$10	Provided by site coordinator directly to patient after completion of consult
Follow-up survey (email)	$10	Mailed to patient by site coordinator
One-on-one interviews (telephone, video, or in-person by site coordinator)	$20	Mailed to patient by site coordinator
One-on-one interviews (telephone, video, or in- person by UW research team)	$20	Provided by UW research team directly to patient
Focus groups	$20	Provided by UW research team directly to participants

9.0 ADVERSE EVENTS

We do not anticipate any additional adverse events related to participation in this study beyond usual care.

If a participant reports feeling distressed as a result of study participation, either as a result of being audio-recorded or completing a study survey, they may choose not to continue or to complete them and/or speak with the site staff. Participants experiencing any physical or psychological complications related to their treatment should discuss this with their treating physician.

10.0 MEASURES

All data is being collected for purposes of research. Data will not be available to clinic staff and will not be used to guide clinical decision making.

10.1 Definitions of Data for Primary Objective

10.1.1 Primary Outcome

Our primary outcome is patient power.

Measures of Power: Power is defined as "patients' self-perceived capacity to influence the decision-making encounter"[1]. We operationalize this construct in two ways:

- **Patient's Self-Efficacy in Patient-Physician Interactions (PEPPI):** This is a validated patient survey initially developed to measure the ability of patients to gather medical information and talk to doctors about their medical concerns.[74, 78] For this study, we are using the validated PEPPI-5 point scale.
- **Active Patient Participation Behaviors:** This is a validated system to assess active patient participation during the surgeon consultation developed by Street, et al. and is measured off the audio-recording of the consultation.[65,75,76]

10.1.2. Secondary Outcome

Our secondary outcome is patient knowledge.

Measures of Knowledge: We measure patient knowledge and personal values relevant for breast cancer surgery using the **Decision Quality Instrument-Breast Surgery**.[78] This is a validated scale developed with the intent of assessing the extent to which patients were informed about breast cancer surgery and received treatment in-line with their personal values. As we measure knowledge after the surgical consult, our comparison is between the decision aid and surgeon consult versus the consult alone. It is important to include this secondary outcome since prior studies which evaluated them included few socioeconomically disadvantaged patients.

10.1.3 Predictor Variables

The primary explanatory variable is intervention group assignment (decision aid yes/no). We define socioeconomic disadvantage to be those patients living in the 20% most disadvantaged neighborhoods according to the Area Deprivation Index, consistent with prior research in cancer.[60-63]

10.1.4 Additional Measures

We collect additional measures which we define as Preparatory/Interactional barriers and Accessibility barriers (see conceptual model, figure 2) for our analysis.

10.1.4.1 Preparatory Barriers

- Patients Expectations for Their Role in a SDM Encounter

  Control Preferences Scale (baseline patient survey): The Control Preferences Scale (CPS) is validated survey instrument that was developed to measure patient's preferred and/or perceived roles in their health-care decision-making; it consists of a single item. The roles range from the individual making the treatment decision, through the individual making the decision jointly with the physician, to the physician making the decision.[77]

  Preparation for Decision Making Scale (follow-up patient survey): The ten-item Preparation for Decision Making (PrepDM) scale was developed to evaluate decision processes relating to the preparation of patients for decision making and dialoguing with their practitioners.[78] It has been demonstrated to be reliable and valid, and discriminates based on perceived usefulness of decision support tools.

- Patients' Knowledge about the Decision Faced:

  "How informed do you feel?" (baseline patient survey): We will ask how informed patients feel about the surgical options for breast cancer before their consultation with the surgeon.

  SURE Decisional Conflict Scale (follow-up patient survey): The 4-item SURE (Sure of myself; Understand information; Risk-benefit ratio; Encouragement) screening test was developed to help health professionals identify patients with clinically significant decisional conflict as quickly as possible.[81]

10.1.4.2 Interactional Barriers

- Power Imbalance

  Surgeon Facilitative Behaviors (audio-recording of consult): In addition to assessing active patient participation, the validated system developed by Street, et al., also measures the degree to which physicians used partnership-building and supportive talk.[65,75,76]

  Health Care Climate Questionnaire (follow-up patient survey): The Health Care Climate Questionnaire (HCCQ) is a validated patient survey that was developed to assess patient's perceptions of the degree to which their specific doctor is autonomy supportive. It consists of 15 items. We will use this instrument to assess Patient Self Autonomy and Trust in Provider.

- Clinicians' Interpersonal Characteristics

  Surgeon recommendation (audio-recording of consult): We will collect whether the surgeon provided a recommendation, and when in the consultation this occurred. We will classify this as early recommendation versus late/no recommendation based on whether it occurs in the first quarter of the consultation.

  Family member presence (audio-recording of consult): We will observe if a family member is present during the visit with the surgeon.

  Practitioner Orientation Scale (surgeon baseline survey): The Practitioner Orientation Scale measures the roles that doctors believe they should play in the course of their interaction with patients. The total score for this 18-item scale, ranging from patient-centered to doctor-centered, can be calculated in addition to two sub-scores. The first nine-item sub-scale, Sharing, reflects the extent to which

the respondent believes that patients desire information and should be part of the decision making process. The second nine-item sub-scale, Caring, reflects the extent to which the respondent sees the patient's expectations, feelings, and life circumstances as critical elements in the treatment process.[80]

10.1.4.3 Accessibility Barriers

- Reach

  Proportion of patients sent the decision/total number of patients seen in clinic after cross-over to intervention arm. This will be calculated using the numbers of patients who were offered the decision aid and who were sent the decision aid, and either billing records or clinic schedules (site preference as best way to determine number of patients seen during time period that would have be eligible to receive the decision aid).

- Reason for decline of decision aid

- Accessibility

  We use adaptations of the four principles of accessibility outlined by in the Web Content Accessibility Guidelines (perceivable, operable, understandable, and robust), combined with other key concepts relevant to our study.[81] We will collect data on acceptability of email delivery, how patients who accepted the decision aid used it, and the acceptability of the decision aid itself via survey. We will record the number of times the site was accessed by each patient via the Health Dialog company.

- Health Literacy

  We use the Brief Health Literacy Screen (BHLS) to evaluate health literacy in clinical practice.[82] The BHLS is a validated measure that has been evaluated in both the research and clinical setting. It consists of three items and takes approximately 1 minute to complete.

10.1.4.4 Patient, Surgeon and Clinic Characteristics

- Patient characteristics

  We will abstract patient demographic (age, race, zipcode+4 to calculate ADI, payer), clinical (AJCC stage, estrogen/progesterone receptor and HER2neu) and treatment factors (surgery, radiation, systemic therapy, hormonal therapy) from medical records.

  The zipcode+4 will be used to calculate the Area Deprivation Index (ADI) score. The ADI represents an area-based measure of socioeconomic deprivation based on 17 Census-based markers of socioeconomic status. A publically available dataset provides an ADI score for each zipcode+4 in the United States. The raw ADI score will be transformed into a 20 category quantile-based measure (every 5th percentile) using national ADI thresholds. After the transformation into the categorical variables, the raw zipcode+4 and the raw ADI score will be destroyed.

- Surgeon characteristics

  We will obtain gender, race, ethnicity, fellowship training, percent of practice that is breast, and years in practice. This will be included in the baseline survey.

- Clinic characteristics

  We will obtain number of staff, presence of a navigator, annual breast cancer volume, and payer mix. This will be collected by each site's coordinator.

10.1.5 Overview of Measures Collected from Patients

Measure	Survey prior to consult	Audio-recording of consult	Survey following consult	After completion of treatment*
Patient's Self-Efficacy in the Patient-Physician (PEPPI)			X	
Active Patient Participation Behaviors		X		
Decision Quality Instrument-Breast Surgery	X		X	
Control Preferences Scale	X			
Preparation for Decision Making Scale			X	
How Informed Do You Feel?			X	
SURE Decisional Conflict Scale			X	
Health Care Climate Questionnaire			X	
Family Member Presence		X		
Accessibility	X		X	
Brief Health Literacy Screen	X			
Patient Characteristics				X

* Defined as after completion of first course treatment (surgery, chemotherapy, radiation). Endocrine therapy and her2neu targeted therapy may be ongoing.

10.1.6 Overview of Measures Collected from Surgeon

Measure	Survey Prior to Start of Data collection	Audio-recording
Practitioner Orientation Scale	X	
Surgeon Facilitative Behaviors		X
Surgeon Recommendation (yes/no and timing within consultation)		X
Surgeon Characteristics	X	

10.1.7 Overview of Measures Collected from Clinic

Measure	Baseline	End of Decision Aid Intervention
Clinic characteristics	X	
Reach of Intervention		X

10.2 Definitions of Data for Secondary Outcomes

10.2.1 Patient Interviews

We use findings from Aim 2 to inform stratified purposeful sampling and interview content.[83] Two sets of interviews will be conducted: One addressing Accessibility Barriers and one addressing Preparatory/Interactional Barriers.

10.2.1.1 Accessibility Barriers

Telephone, video, or in-person interviews will be conducted by the site research coordinator. We think any modality is acceptable given the non-sensitive nature of these interviews.[84,85] A domained interview script is included in the Appendix VII. Sample interview questions include:

- Tell me about the type of communication you had with the surgeon's office prior to the first consult
- Please describe how the decision aid was offered to you, and share your initial impressions.

We will use the collected quantitative data to guide the interviews. For patients who declined the decision aid, we will use the documented reason for declining as a probe and will ask about alternative forms of information they may have used. For patients who accepted but reported difficulty accessing or using the decision aid, we will ask them to walk us through their experience receiving the decision aid, probing on reported challenges. A final question will ask patients about perceived opportunities to improve experiences in accessing the decision aid.

10.2.1.2 Preparatory/Interactional Barriers

Telephone, video or in-person interviews will be conducted at a private location convenient to the patient. In-person interviews are optimal for this more sensitive topic area; however, telephone or video may be necessary in some circumstances (i.e COVID-19 pandemic). We estimate interviews will occur an average 18 months after the surgeon consult. Although this creates the potential for recall bias, this sequential approach is necessary as the preliminary findings from the quantitative data (primary objectives) are critical for both our sampling strategy and interview content. We will use techniques effective in other studies to minimize recall bias, including prompts from patients own survey responses,[86] and will preferentially sample patients enrolled later in the study period to minimize time between consult and interview. Prior research demonstrates that breast cancer patients' perceived role in decision making has a lasting impact on quality of life, even >5 years from diagnosis.[87,88] This data suggests that the decision making process is significant enough that patients – especially those experiencing barriers to engagement— will be able to describe their experience even after delay. We anticipate that 40-50 patients will be needed to achieve informational redundancy,[89-91] with half the interviews focused on preparatory and half on interactional barriers.

Each interview will begin by asking patients: "Walk me through how you and your surgeon made the decision for type of surgery". We will specifically probe whether patients perceived that they were able to participate in decision making and express their treatment goals, and whether they felt that the surgeon understood what was important to them. We will then ask open-ended questions that focus specifically on barriers that patients experienced. Using our example of "power imbalance" as a prioritized interactional barrier, a sample question would be, "Tell me how you felt sharing what was important to you with your surgeon." We will also ask patients, "Based on your experience, what do you think could be done to help patients like yourself?"

10.2.2 Patient and Clinic Stakeholder Focus Groups

We will begin each focus group with a review of the prioritized barriers to engagement (primary objective) and targets for potential adjunct interventions (interviews from secondary objective). To ensure that this discussion is as salient as possible to stakeholders, we will present each barrier using quotes from patients who experienced the barrier. We will briefly elicit participants' perceptions of how these barriers impede engagement as we anticipate that the group discussion will provide additional insight. We will then present participants with potential adjunct interventions to target these barriers (Table 7), identified through the existing literature. We will elicit feedback from stakeholders regarding the anticipated effectiveness and feasibility of these interventions in their clinical settings, and needed adaptations. We anticipate that adaptation will be necessary, as identified interventions are not likely to have been developed within clinics caring for a high proportion of disadvantaged patients. We will then prompt participants to brainstorm other solutions to address prioritized barriers. At the completion of the focus group, we will use nominal group technique to identify three interventions perceived to have the greatest potential.[92,93] We will ask stakeholders to evaluate each

Table 7. Examples of possible interventions

Intervention	Goal for Patient	Target Barrier
Delivery using electronic health record	Decrease barriers to access	Accessibility
Pre-consult pairing with decisional coach	Improve preparation	Preparatory
Intervention to increase family member participation	Increase confidence in interacting	Interactional
Intervention to increase surgeon facilitative behaviors	Increase perception that input is valued	Interactional

intervention using a modified Ottawa Acceptability tool.[94]

11.0 END OF INTERVENTION/STUDY

11.1 Duration of Study

Site level participation will end when the last patient enrolled completes their breast cancer treatment. A subset of sites will have a focus group conducted using their patients. For those sites, participation will end when that focus group is completed.

Clinic stakeholder participation ends when the last patient enrolled completes her surgical consultation. For the subset of clinic stakeholders that will participate in the focus groups, study participation concludes at the end of that focus group.

Patient-level participation surveys and audio-recording will end after they complete the follow-up survey. Chart reviews for them will be completed after their breast cancer treatment is complete to accurately reflect care received. For the subset of patients who participate in the interview and/or focus group, study participation concludes at the end of that interview/focus group.

Please see the study calendar (Section 5.0) for follow up time periods.

11.2 Criteria for Discontinuation of Study

In the absence of study procedures due to adverse event(s), patient participation may continue until one of the following criteria applies:

- Patient explicitly decides to withdraw from the study
- Termination of the study by sponsor

If a patient has a subsequent decline (such as opt-out from an interview), the decline applies only to that specific research activity and does not apply to any prior data collection unless specified by the patient.

11.3 Follow-up

11.3.1 Duration of Follow-up

Following the surgical consult where informed consent was obtained, the baseline survey (T0) completed, and consult audio-recorded (T1), a follow-up survey (T2) will be emailed to patients within 24 hours of the consultation. If no response is received, study staff will send reminders to the patient via email and telephone calls (maximum of six attempts), with the goal of completing the survey within 3 weeks. To avoid missing data, study staff will also approach patients for survey completion at a follow-up visit if this is scheduled within the 3-week period. Patients will have chart review performed after completion of their breast cancer is treatment.

11.3.2 Lost to follow up

Patients will be considered lost to follow up if attempts to reach them for the follow-up survey (T2) is unsuccessful. However, patients will still have their chart review performed, unless the patient explicitly decides to withdraw from the study and withdraws consent.

11.4 Managing Ineligible Patients

Baseline data and all post-baseline data up until the point the patient was found to be ineligible must be submitted; however, no data is required following the point that the patient was found to be ineligible. The patient should complete the consent withdrawal form and indicate “consent withdrawal for all follow up.”

12.0 STATISTICAL CONSIDERATIONS

As part of Update #06, the statistical considerations and analysis plan for the co-primary objectives have been amended. The rationale for these revisions was twofold. First, we needed to revise the primary analysis plan to address the current challenges, which were related to the COVID pandemic. Second, we needed to ensure that the revised primary analysis plan was compatible with the power calculations and that the power calculations reflected what realistic effect sizes the study would now be able to detect given these challenges. The amended statistical considerations and analysis plan for the co-primary objectives are detailed below.

12.1 Analysis Plan for Co-Primary Objectives

12.1.1 Primary Analysis

Co-Primary Objective #1: The goal is to evaluate the effectiveness of a decision aid in increasing patient engagement in decision making in clinics serving a high proportion of socioeconomically disadvantaged patients. We will follow an intention to treat approach, using all available data, regardless of group assignment or whether patients viewed the decision aid. The data as collected will have a nested structure: patients nested within surgeons within surgical clinics, this last level being the formal level of intervention. Descriptive analyses will summarize patient, surgeon, and clinic characteristics. When reporting these summary data, we will report as "NR-Not reported" any cell where the number of patients is less than five to avoid inadvertent patient identification. We will test intervention effects in the framework of generalized linear mixed effects models with an "intervention versus usual care" dummy variable (which varies over patients within each surgeon and clinic), a surgeon random effect, and a clinic random effect. Analysis models will include two additional parameters, for wave and wave-squared, respectively to flexibly control for potential time effects and to allow for a non-linear relationship with outcome measures. We will include a post-COVID restart dichotomous variable that reflects whether a patient is enrolled during the post-COVID restart time period for a given site (yes/no). We also will include time (in units of wave) post-COVID in all models. These indicator and time parameters allow for the control of sites restarting the trial post-COVID shut down at different times. This leads to the following planned linear mixed effects model for the two primary outcomes (y) in Aim 1, namely, PEPPI-5 self-efficacy and Active Patient Participation Behaviors:

$$y_{ijk} = \beta_0 + \theta x_{ijk} + \beta_1 t_{1ijk} + \beta_2 t_{1ijk}^2 + \beta_3 z_{ijk} + \beta_4 t_{2ijk} + u_k + u_{j(k)} + e_{i(j(k))}$$

Here, y_{ijk} denotes the response of the ith level-1 unit (patient) of the jth level-2 unit (surgeon) of the kth level-3 unit (clinic). The variable x_{ijk} (=0, 1) denotes the intervention status of patient i from surgeon j within clinic k. The variables t_1 (=1, 2, 3, 4, 5, 6, 7) and t_1^2 (=1, 4, 9, 16, 25, 36, 49) are the linear and quadratic terms for wave with the corresponding coefficients β_1 and β_2 representing the fixed linear and quadratic effects to model the underlying secular trend. The variable z (=0,1) denotes if patient i was enrolled during the post-COVID restart time period for a given clinic with the corresponding fixed effect coefficient β_3. The variable t_2 (=0, 1, 2, 3, 4, 5, 6) denotes if patient i was enrolled within the first wave post-COVID restart time period ($t_2 = 1$); within the second wave post-COVID restart time period ($t_2 = 2$); and so on through the sixth wave post-COVID restart time period ($t_2 = 6$). The maximum value is $t_2 = 6$ because when the stepped-wedge design was suspended all clinics were at least enrolling part way in wave 2 of the possible 7 waves. For patients enrolled prior to the post-COVID restart time period the value of $t_2 = 0$. The random effect u_k is the level-3 (clinic) random intercept effect (random clinic variance); the random effect $u_{j(k)}$ is the level-2 random intercept effect for

the $j(k)^{th}$ level-2 unit (random surgeon variance nested in clinics); the random effect $e_{i(j(k))}$ is the random individual variance within surgeons nested in clinics. The term β_0 is the intercept. The term θ represents the intervention effect of the decision aid and is the primary effect of interest.

Models will adjust for the a priori specified patient characteristics described above that are likely to be strongly predictive of the endpoints. In the event that the approximately continuous outcomes PEPPI-5 self-efficacy and Active Patient Participation Behaviors are not approximately symmetrically distributed, variable transformation will be considered (e.g., square-root transformation). Further, as part of the planned efficacy analysis we will fully test the variance-covariance structure of the model, while blinded to any treatment effects, which may lead to a more parsimonious model.

We will use linear mixed effects models for the approximately continuous and symmetrically distributed outcomes (Active Patient Participation Behaviors, PEPPI-5, breast cancer knowledge, perceived knowledge) and logistic random effects models for binary outcomes (Decision Quality Instrument- Breast Surgery). Should count variable endpoints (e.g., Active Patient Participation Behaviors) exhibit floor effects, which we do not anticipate,[95] log-linear random effect models will be used to estimate the relationship of these endpoints to intervention group. In addition to accounting for association in the data at various levels of clustering, the mixed models will allow for estimation of the percentage of variance in outcomes accounted for by differences at the surgeon, and clinic level. This is critical as it will suggest at what level adjunct interventions may be most effective. Models will be estimated and tested using SAS v9.3 (PROC MIXED and PROC NLMIX).[96]

Co-Primary Objective #2: The goal is to test the extent to which the effect of a decision aid on patient engagement is mediated through the mitigation of preparatory and interactional barriers, and determine if persistent barriers are disproportionately experienced by socioeconomically disadvantaged patients. As a decision aid can only be effective if patients are able to access/review it, we first assess for accessibility barriers to decision aid use. We will then examine the impact of preparatory and interactional barriers on engagement.

Accessibility Barriers: We will examine barriers to accessing the web-based decision aid separately, as only patients who receive the intervention can experience these barriers. Understanding accessibility barriers is critical to ensure the intervention will not worsen disparities. This is a largely descriptive analysis that will guide sampling and interview content for the patient interviews in Aim 3. We will calculate reach of the decision aid intervention for the study cohort, and for socioeconomically disadvantaged patients. We will summarize reasons why patients declined the decision aid. We will summarize other survey responses regarding decision aid accessibility (Table 5) and perform exploratory analyses evaluating the association with socioeconomic disadvantage, using chi-square tests and odds ratios for categorical variables and ANOVA mean differences for continuous variables, respectively. Due to the exploratory nature of the analysis, sample size and power are not calculated.

Preparatory and Interactional Barriers to Patient Engagement:

Decision Aid Impact on Engagement through Mitigation of Barriers

We will first perform analyses to test and quantify the extent to which the effect of the decision aid intervention on patient engagement endpoints is mediated through the mitigation of candidate preparatory and interactional barriers. We will quantify the indirect effect relative to the total effect of the decision aid in joint linear structural equation models (SEM) for the endpoint and the candidate mediators, including random surgeon effects and random region effects for each of the mediator and endpoint parts of the models. Each of the preparatory and interactional Barriers will be represented by a continuous latent construct in the SEM, with 3 and 5 observed items for Preparatory and Interactional Barriers, respectively. Binary or count variables will be modeled via latent continuous construct in the SEM. Structural equation models will be specified, estimated and tested using Mplus v7.3.[97] The use of unidimensional latent constructs increases power and reduces the number of tests, relative to testing each observed item separately. Standardized coefficient estimates from the SEM, representing total, direct, and indirect effects, will allow for the determination of whether the mechanism by which the decision aid leads to increased patient engagement is through the mitigation of preparatory and/or interactional barriers (see Figure 7 for simplified example). For example, if coefficient X is important but Y is not, we can conclude that although the decision aid leads to statistically significant changes in interactional barriers, this is not the mechanism through which the decision aid increases patient engagement. Alternatively, if only coefficient Y is important, we can conclude that although the interactional factors represent a significant barrier to patient engagement, the decision aid does not significantly impact the barrier. Finally, if we find significant indirect effects (represented in the figure by the dashed line leading from the decision aid to engagement that runs through interactional barriers), we can conclude that receipt of the decision aid leads to a change in the interactional barrier, which leads to improved patient engagement. Statistically, coefficients X and Y will be tested together, with the null hypothesis being that the product XY=0. If either latent construct for preparatory or interactional Barriers is significant, then each individual observed item will be tested, post hoc, as candidate barriers. Candidate barriers for the next models will be barriers for which the indirect effect is significant and/or barriers associated with lower levels of engagement (i.e. coefficient Y). Those barriers associated with lower level of engagement will be considered persistent and prioritized for inclusion in the qualitative interviews (Aim 3).

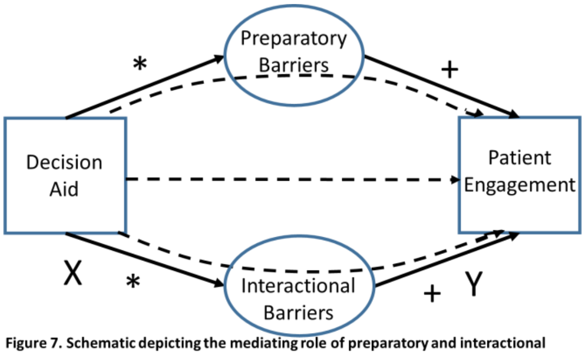

Figure 7. Schematic depicting the mediating role of preparatory and interactional barriers on engagement. The dashed lines represent the test for the overall effect of whether the decision aid improves engagement through that pathway. **Effect of decision aid on barriers; +Effect of barriers on patient engagement.*

Persistent Barriers Experienced by Socioeconomically Disadvantaged Patients

We will estimate logistic and Poisson regression models with socioeconomic deprivation as the explanatory variable, and each candidate persistent barrier as described above as the outcomes, in order to assess the extent to which socioeconomic disadvantage is associated with persistent barriers to engagement. As with Aim 1, models will include surgeon and clinic random effects to account for patient clustering. Findings will guide the sampling strategy for patient interviews (Aim 3) and will be incorporated into the interview content.

12.1.2 Sample Size and Power Calculations

Co-Primary Objective #1: Based on current patient enrollment, we estimate a sample size of 563 patients. This has been reduced from our original estimate of 1,050 patients. We assume 23 surgeons will participate. Aim 1 has two primary endpoints: PEPPI-5 self-efficacy and Active Patient Participation Behaviors (count). We desire a family-wise two-sided Type I error rate of α=0.05; under a Bonferroni correction, tests will be conducted with nominal of α=0.05/2 = 0.025.

Using the revised analysis model and updated accrual projections, we estimated Fisher's information for the regression parameters when the study will have complete data; inverting that information we obtained an estimate of the standard error for the intervention effect parameter, thereby computing minimally detectable Cohen's-*D* effect size for each primary endpoint at 80% and at 90% power. As a sensitivity analysis, we considered varying estimates of ICCs to ensure robust findings.

Self-Efficacy: Assuming PEPPI-5 has a standard deviation (SD) of 4.3 and both within-surgeon and within-site-between surgeon ICC of 0.07, we will have 80% power to detect effects as small as 0.55*4.3=2.4 and 90% power to detect effects as small as 0.63*4.3=2.7 on a scale of 5-25. We consider a difference of 2 points to be clinically meaningful.

Active Patient Participation Behaviors: Assuming the active patient participation behavior count has a standard deviation of 23.1 and a within-surgeon ICC of 0.01 and within-site-between surgeon ICC of 0.0001, we have 80% power to detect a difference of 0.50*23.1=11.6 and 90% power to detect a difference as small as 0.57*23.1=13.2 on a 0-150 scale. We consider a difference of 10 to be clinically meaningful.

As part of the process to ascertain whether we would achieve our study goals despite the lower than planned accrual and the required revisions to the analytic plan in response to the COVID-19 pandemic, we also performed a power calculation using the estimated standard deviation for each of the two co-primary endpoints obtained from data collected during Wave 1 data (all patients are in usual care in Wave 1 and all sites had completed Wave 1). To maintain the integrity of the trial, we leveraged the ongoing collaboration with non-Alliance study statistician, Dr. Paul Rathouz, who is not intimately involved in the daily operations, data monitoring, and study conduct. The Alliance study statistician, Dr. David Zahrieh, provided Dr. Rathouz, with the estimated standard deviations, but no granular data. The estimated standard deviations were then used by Dr. Rathouz to calculate the revised power to detect our a priori protocol-defined study hypotheses.

With the new analytic plan, the new projected sample size, and the revised standard deviations, we have the following updated statistical power for each of the two co-primary endpoints associated with Co-Primary Objective #1:

Self-Efficacy: Assuming PEPPI-5 has a standard deviation (SD) of 3.4 and both within-surgeon and within-site-between surgeon ICC of 0.07, we will have 80% power to detect effects as small as 0.55*3.4=1.87 and 90% power to detect effects as small as 0.63*3.4=2.14 on a scale of 5-25. We consider a difference of 2 points to be clinically meaningful.

Active Patient Participation Behaviors: Assuming the active patient participation behavior count has a standard deviation of 19.1 and a within-surgeon ICC of 0.01 and within-site-between surgeon ICC of 0.0001, we have 80% power to detect a difference of 0.50*19.1=9.6 and 90% power to detect a difference as small as 0.57*19.1=10.9 on a 0-150 scale. We consider a difference of 10 to be clinically meaningful.

Co-Primary Objective #2: Mackinnon *et al.* have shown that one of the most accurate tests for mediation or indirect effect is the "asymmetric distribution of products" test[100] and have provided extensive simulation studies for estimating power for a variety of mediation tests.[101,102] PowMedR software[103] was developed based on that work, which we exploit here. We assume a sample size of 563 and then deduct 23 df for surgeon effects and 7 df for number of waves; we estimate an effective sample size of 533. In addition, because we are testing two mediators, we set α=0.05/2 = 0.025 for these two tests. Suppose we have small mediation effects with R^2=2% for the effect of the intervention on the *latent* barrier mediator (Figure 7) and for the effect of the latent mediator on PEPPI-5 score or on active patient participation behavior count. Suppose additionally that the three observed items have correlation of at least r=0.60. Then, the average of those three items will have reliability of $\lambda = \{r + (1/3)(1 - r)\}$,, yielding net R^2 values between the intervention and the *observed* mediator and between the observed mediator and PEPPI-5 score or active patient participation behavior count of λR^2=0.82*.02=1.6%. Using the PowMedR software, we will have 60% power to detect mediation with small effect size and >99% power to detect mediation with medium effect size λR^2=0.82*.15=12.3%. Power will be higher with five items.

We will extend the period of enrollment for sites that are still recruiting within the stepped wedge design as of 8/30/2021. The extended recruitment beyond Wave 7 will continue for six sites until 12/31/2021 (see Section 12.1.3). While these additional patients will not contribute to the first co-primary objective, they will be included in the analysis for this 2nd co-primary objective; all of these additional patients will be exposed to the study intervention. We anticipate, at a minimum, that there will be 50 such patients exposed to the intervention across these six sites. We re-estimated power assuming such an increase, which leads to an increase in the power for detecting small effects from 60% to 65%.

Once mediating barriers are detected, we use a model similar to that in Aim 1 to test for an association of each barrier to a baseline indicator of socioeconomic disadvantage. We assume 30% of the sample will be disadvantaged, evenly distributed among clinics and surgeons. Assume we will be testing up to four barriers; as we desire a family-wise two-sided Type I error rate of α=0.05, under a Bonferroni correction, tests will be conducted with nominal of α=0.05/4 = 0.0125. Extending the same method of computing power as in Aim 1, we will have power of 80% for an effect size of 0.31 and 90% power to detect an effect size of 0.35.

We then re-estimated power assuming an additional 50 intervention patients enrolled beyond Wave 7 across six sites. As a result, the study will have 80% power to detect an effect size of 0.30 and 90% for an effect size of 0.34.

12.1.3 Accrual Time and Study Duration

The sample size to achieve our co-primary objectives is 563 patients. Our planned stepped wedge design includes 7 waves of approximately 10 weeks (total 70 weeks, ~18 months). We also include a Lead-in Period prior to starting to optimize recruitment strategies. For each clinic, one wave is used for implementation of the decision aid at the time of cross over, yielding 6 waves for data collection. To achieve our study goal, we anticipate sites will need to approach an average of 9 patients per month and enroll an average of 7 patients per month. However, as variation in patient volume between clinics is expected, we will ask clinics to over-enroll each month to ensure successful sample size accrual.

Step wedge accrual rules: Each wave within our stepped wedge design is set to last 10 weeks. However, under-recruitment may occur during any given wave at any given site. Under-recruitment could adversely impact sample size and study conclusions. We have

updated our accrual rules to acknowledge under-recruitment. This decision was made at a relatively early stage in the study (less than half of the sites had started accrual and some for only a few weeks). Further, decision-makers were blinded to the intervention status or time of cross-over in making the decision. The updated accrual rules state that:

In a wave, if the current accrual for a given site is <2/3 of the stated accrual goal for that site at the end of the wave, then the wave will be extended by three weeks for that site. This rule is to be applied at the wave-by-site level, independent of the previous history at that (or any other) site and transition time to the intervention. Only one extension is possible at the wave-by-site level, so that the maximum wave length is 13 weeks. A single site could be extended in multiple different waves, as the rule is applied independent of history.

A critical component of this rule is that the accrual process will be supported by ongoing, uniformly applied recruitment monitoring and support from the Alliance and the UW team. This will serve to encourage a steady rate of accrual across time.

This rule was selected as it best balanced statistical validity with need to meet accrual goals and complete study in a timely fashion. It also was able to address both situational and chronic low accrual at times.

Extended recruitment beyond Wave 7: We have experienced under-recruitment throughout the study as a result of a number of factors, including and especially the COVID-19 pandemic. To compensate for the slower than anticipated accrual rate, we will extend the period of enrollment for the sites that are still recruiting within the stepped wedge design as of 8/30/2021. Patients enrolled after Wave 7 is completed at each of the sites will not contribute to the first co-primary objective. The additional patients recruited beyond Wave 7 will be included in the analysis for the 2nd co-primary objective and the sampling pool for the secondary objective. As of 8/30/2021, six of the original ten sites were actively recruiting within the stepped wedge design. The extended recruitment beyond Wave 7 would continue for the six sites until 12/31/2021. We anticipate enrolling 50 additional patients, at a minimum; however, based on the most recent accrual rates it is likely that we may enroll up to 62 patients for a total targeted sample size 563 + 62 = 625 patients.

12.1.4 Missing Data

We measure our primary outcome by two methods: analysis of the audio-recording and the analysis of the follow-up survey. As patients consent immediately prior to the consultation, we anticipate that we will have minimal missing-ness of that component of the primary outcome. To decrease missing data from our follow-up survey, assessment burden and drop-out, we have limited the number of survey questions and provided incentives. We have included flexible means of contacting patients for the post-consultation survey, including email (primary modality), telephone, or in-person at a clinic visit. If data are missing on predictor variables of interest, data will be imputed using multiple imputation (MI) techniques (MI using chained equations).[104,105] MI produces valid statistical estimates under missingness at random (MAR), while also accounting for additional uncertainty arising from missing data. We will perform sensitivity analyses to determine the degree to which results are sensitive to the MAR assumption. We will also account for survey non-response using an inverse probability weighting approach if we should find that there is differential survey non-response between the intervention and usual care groups according to patient characteristics that also influence the outcomes of interest.

We will consider patients as participants if they complete the audio-recording and only consider them to have dropped out if they withdraw consent. If a participant withdraws

consent, we will document: 1) the reason for dropout; 2) who decided the participant would drop out; and 3) whether the dropout involves some or all types of participation.

12.2 Analysis Plan for Secondary Objectives

12.2.1 Interviews

All interviews will be audio recorded and transcribed verbatim. Early interviews will be reviewed independently by at least three investigators to generate a preliminary list of conceptual codes that will be used to reduce the data. We will include team members with unique clinical backgrounds to enrich the coding process. The identified coding schemes will be compared and discussed to reach consensus for a preliminary coding taxonomy, which will guide primary coding of subsequent interviews. If new concepts emerge during subsequent interviews, this coding taxonomy will undergo iterative revisions and earlier interviews will be recoded to include the emerging constructs. We will use visual displays to broadly categorize the data and organize it into overarching themes, facilitating comparisons between our patient cohorts.[106] We will use *NVivo* basic program (QSR International-Melbourne) to manage data and facilitate analysis.

We will take two approaches to data analysis. First, we will perform directed content analysis to identify and categorize barriers deductively.[107,108] Although we measured discrete concepts related to accessibility, preparatory, and interactional barriers in our quantitative data collection, it is likely that not all relevant concepts will have been captured. Therefore, we will also perform inductive coding to identify previously unrecognized barriers. The goal of this first analytic step is to confirm and enhance our conceptual model. As a second step, we will perform inductive content analysis to characterize the mechanisms by which the identified barriers limited patient engagement despite a decision aid. We will also explore how patients were able to overcome, or not overcome, identified barriers. This information will provide further insight of the mechanisms supporting and impeding engagement. We will compare and contrast the experiences reported by patients based on their characteristics to elucidate the interaction between socioeconomic disadvantage and patient engagement.

We use an explanatory mixed methods approach to integrate the quantitative (primary objectives) and qualitative (secondary objective) findings in order to identify potential targets for adjunct interventions.[91] The quantitative analysis will identify and prioritize persistent barriers to engagement that exist with and without a decision aid. By understanding patients' experiences with these barriers, we can obtain insight into potential ways to ameliorate them. From this integration, we will identify a list of potential targets for adjunct interventions, prioritized by the proportion of patients who experience the barrier and perceived impact of the barrier on engagement.

12.2.3 Interview Sample Size

We will conduct telephone, video, or in-person interviews with 30 patients who experienced accessibility barriers; we anticipate that this will be adequate to obtain informational redundancy.[89-91]

In addition, we will conduct telephone, video or in-person interviews with 50 patients who experienced preparatory or interactional barriers to patient engagement.

12.2.4 Focus Groups

We will fully audio-record and transcribe focus groups, and take field notes to augment these observations. We will perform directed content analysis to identify those components of the discussion directly relevant to our goal of identifying adjunct interventions to target identified barriers. The deliverable from the focus groups will be a list of potential adjunct interventions to target prioritized barriers.

12.2.5 Focus Group Sample Size

We plan to conduct three patient focus groups at three separate clinics. We anticipate inviting 10-12 women to each focus group, with an anticipated focus group size of 8 (total 24 patients).

We will also plan to conduct two focus groups with clinic stakeholders from our participating clinics, including both surgeons and nurses. We anticipate inviting 15 stakeholders to each focus group, to obtain a final size of 8-10 participants; we will aim for an even distribution of surgeon and nurses.

12.3 Study Reporting

12.3.1 This study will be monitored by the Alliance Data Safety Monitoring Board (DSMB), an NCI-approved functioning body. Reports containing efficacy, adverse event, and administrative information will be provided to the DSMB every 6 months as per NCI guidelines.

12.3.2 Results Reporting on ClinicalTrials.gov: At study activation, this study will have been registered within the “ClincialTrials.gov” web site. The Primary and Secondary Endpoints (i.e., “Outcome Measures”) along with other required information for this study will be reported on ClinicalTrials.gov.

12.4 Inclusion of Women and Minorities

For clinic stakeholders, there are no specific exclusion criteria based on sex/gender, race or ethnicity as our sampling will be any current clinic staff member that is involved in the care of newly diagnosed breast cancer patients and the implementation of the decision aid.

For patients, all newly diagnosed female breast cancer patients, regardless of age, race, or ethnicity, will be offered the decision aid and eligible to participate in the research components of the study. We will include minority patients in numbers proportionate to the demographics of the ten surgical practices that we recruit for this study.

We will exclude men diagnosed with breast cancer from our study. Although men are recommended surgery to treat breast cancer, male breast cancer is relatively rare. Additionally, the decision for type of breast cancer surgery is simpler for men compared with women. Practically speaking, breast conservation is often not an option given the lesser amount of breast tissue. Additionally, mastectomy does not have the same implications with regards to body image, sexuality, etc. as it does in women. Because of this, the decision aid used in our study is applicable only for a female population.

Estimated enrollment for both patients (625) and clinic stakeholders (10 clinic staff, and 30 surgeons). Because we have not selected participating clinics, our estimates are based on a large national cancer registry database, the National Cancer Data Base (NCDB). The NCDB is a program of the American College of Surgeons Comission on Cancer and the American Cancer Society and captures approximately 70% of all newly diagnosed cancers in the U.S., with >60% of these clinics being community cancer programs. This gives us the best estimate of our enrollment.

DOMESTIC PLANNED ENROLLMENT REPORT FOR PATIENTS					
Racial Categories	Ethnic Categories				Total
	Not Hispanic or Latino		Hispanic or Latino		
	Female	Male	Female	Male	
American Indian/ Alaska Native	1	0	0	0	1
Asian	50	0	0	0	50
Native Hawaiian or Other Pacific Islander	8	0	0	0	8
Black or African American	136	0	2	0	138
White	393	0	25	0	418
More Than One Race	10	0	0	0	10
**Total**	598	0	27	0	625

DOMESTIC PLANNED ENROLLMENT REPORT FOR NON-PATIENTS (CLINIC STAKEHOLDERS)					
Racial Categories	Ethnic Categories				Total
	Not Hispanic or Latino		Hispanic or Latino		
	Female	Male	Female	Male	
American Indian/Alaska Native	0	0	0	0	0
Asian	2	0	0	0	2
Native Hawaiian or Other Pacific Islander	1	0	0	0	1
Black or African American	7	4	1	0	12
White	9	12	4	0	25
More Than One Race	0	0	0	0	0
Total	19	16	5	0	40

Ethnic Categories:

Hispanic or Latino – a person of Cuban, Mexican, Puerto Rican, South or Central American, or other Spanish culture or origin, regardless of race. The term “Spanish origin” can also be used in addition to “Hispanic or Latino.”

Not Hispanic or Latino

Racial Categories

American Indian or Alaskan Native – a person having origins in any of the original peoples of North, Central, or South America, and who maintains tribal affiliations or community attachment.

Asian – a person having origins in any of the original peoples of the Far East, Southeast Asia, or the Indian subcontinent including, for example, Cambodia, China, India, Japan, Korea, Malaysia, Pakistan, the Philippine Islands, Thailand, and Vietnam. (Note: Individuals from the Philippine Islands have been recorded as Pacific Islanders in previous data collection strategies.)

Black or African American – a person having origins in any of the black racial groups of Africa.

Native Hawaiian or other Pacific Islander – a person having origins in any of the original peoples of Hawaii, Guam, Samoa, or other Pacific Islands.

White – a person having origins in any of the original peoples of Europe, the Middle East, or North Africa.

13.0 GENERAL REGULATORY CONSIDERATIONS: WAIVERS OF CONSENT

13.1 Waivers of documentation of written patient consent

13.1.1 Pre-screening

Per 45 CFR 46.117c, a waiver of informed consent for the prescreening of study eligibility described in Section 8.1 is justified for the following reasons:

- Pre-screening eligibility review of medical records by clinical research staff does not adversely affect the rights or welfare of subjects because medical record information is screened to establish preliminary eligibility prior to approaching a potential subject about a study,
- Pre-screening eligibility review of medical records cannot practicably be done without the waiver due to the number of services participating in which patients may be eligible. Pre-screening eligibility decreases the burden on patients of introducing a research study to subjects who can easily be identified through pre-screening activities as not eligible for a research study,
- Pre-screening eligibility review of medical records involves no more than minimal risk because the person accessing the information has undergone training in confidentiality of medical records, and the records are viewed solely to pre-screen for eligibility criteria and only minimal information found in the pre-screening process will be recorded for research purposes. Names of patients (and other unique identifiers) who are deemed ineligible will not be recorded.

13.1.2 Decision aid

Decision aids have proven efficacy at supporting breast cancer surgery decisions and can be considered an acceptable practice. Given this, we will not obtain documentation of informed consent from patients to send them the web-based decision aid after clinics cross over to the decision aid intervention. Being offered the decision aid does not constitute research in this study.

13.1.3 Patients who decline the decision aid

Collection of limited demographics: For all patients who decline the decision aid, site coordinators will collect limited additional information about them (medical record number, age, race, ethnicity, insurance status, ZIP code + 4, and reason(s) why declined) to ensure we are not systematically excluding certain populations from our study. As the decision aid is being implemented as an acceptable practice in the clinic, these patients have not declined research. However, understanding demographics of patients that decline the decision aid along with reasons why will greatly increase our understanding of how to disseminate this type of intervention in the future and ensure it is not worsening disparities. In addition, this information will be used to purposefully sample for the telephone, video, or in-person interviews addressing Accessibility Barriers. The ZIP code + 4 will be used to generate the ADI. After the transformation into the ADI variable, the raw zipcode+4 will be destroyed to ensure no PHI is saved. Given the limited amount of information that is collected, a *waiver of informed consent* will be requested for these subjects. A waiver of informed consent is requested because:

- Collecting medical record number, age, race, ethnicity, ZIP code + 4, and reason(s) for declining the decision aid involves no more than minimal risk because the person accessing the information has undergone human subjects training, and information that will be recorded for research purposes is minimal. This data will be tracked to inform future implementation and dissemination studies using this tool.

- Collecting medical record number, age, race, ethnicity, insurance status, ZIP code + 4, and reason(s) for declining the decision aid will not adversely affect the rights or welfare of subjects because no names or other unique identifiers will be collected.
- Spending the time to obtain informed consent for such questions would be an unnecessary burden for the subjects at an already stressful time. Collecting this data on all patients who declined the decision aid is important in order for the information to be meaningful.

Patient Telephone, Video, or In-person Interviews: A subset of patients will be eligible to participate in telephone interviews addressing Accessibility Barriers if they either 1) declined the decision aid or 2) experienced access barriers identified through survey responses.

For the patient interviews assessing accessibility barriers, we will request a *waiver of documentation of informed consent* given the low risk nature of these activities. Participants in each session will be provided with an information sheet describing the research, their participation, the risks, possible benefits, and all other required elements of informed consent. This will be sent either by mail or email prior to the interview. Because a signed consent form would be the only documentation of the participants' identities, we feel a waiver of documentation of informed consent is appropriate. At time of the interview, we will ask verbal permission to record prior to proceeding. If an individual declines the audio recording, the interview will proceed with expanded field notation. Participants may drop out at any time before, during, or after the interview/focus group.

13.1.4 Patients who accept the decision aid

Patient Telephone, Video, or In-person Interviews: Patients who accept the decision aid but report difficulty accessing it or that they did not use it will be eligible for the telephone interviews assessing accessibility barriers. For the patient interviews assessing accessibility barriers, we will request a waiver of documentation of informed consent given the low risk nature of these activities. Participants in each session will be provided with an information sheet describing the research, their participation, the risks, possible benefits, and all other required elements of informed consent; this will be sent either by mail or email prior to the interview. Because a signed consent form would be the only documentation of the participants' identities, we feel a waiver of documentation of informed consent is appropriate. At time of the interview, we will ask verbal permission to record prior to proceeding. If an individual declines the audio recording, the interview/focus group will proceed with expanded field notation. Participants may drop out at any time before, during, or after the interview/focus group.

Patient telephone, video or in-person interviews and focus groups: Patients who participate in the audio-recording (Section 8.3) may be eligible for patient interviews and/or focus groups. For the patient interviews and focus groups, we will request a waiver of documentation of informed consent given the low risk nature of these activities. Participants in each of these sessions will be provided with an information sheet describing the research, their participation, the risks, possible benefits, and all other required elements of informed consent. Because a signed consent form would be the only documentation of the participants' identities, we feel a waiver of documentation of informed consent is appropriate. At time of the interview, we will ask verbal permission to record prior to proceeding. If an individual declines the audio recording, the interview will proceed with expanded field notation. If an individual declines the audio recording, they will not be eligible for participation in the focus group. Participants may drop out at any time before, during, or after the interview/focus group.

13.2 Waiver of elements of informed consent—patients

We request a waiver of the specific element of consent regarding medical injury to patient. This study does not involve medical treatment, and it is not expected that patients will suffer medical injury due to participation in this trial. We have therefore removed the "What happens if I am injured because I took part in this study" section of the NCI Model Consent Template. Omission of this information does not constitute greater than minimal risk and would not adversely affect the patient.

In addition, patients will not be informed that the decision aid is part of the research objectives of this study at the time of informed consent. Omission of this information does not constitute greater than minimal risk and would not adversely affect the patient. Because the study measures the effect of the decision aid on patient engagement, it is necessary to omit the purpose of the study in the informed consent document. Following their participation in the study, patients will be notified that the effect of decision aid use was an objective of the study. Participants will be sent a debriefing information sheet by mail at completion of the study.

Finally, we have provided only the "Study Title for Participants" in the Model Consent form for this study. The "Official Study Title for Internet Search on CT.gov" will be provided upon patient completion of the study. This study will be listed on clinicaltials.gov.

13.3 Waivers of consent—surgeons and clinic staff

Surgeons: We will request a waiver of documentation of informed consent for participation in the stakeholder focus groups given the low risk nature of the focus group. Participants in each of these sessions will be provided with written information describing the research, their participation, the risks, possible benefits, and all other required elements of informed consent. Because a signed consent form would be the only documentation of the participants' identities, we feel a waiver of documentation of informed consent is appropriate. At time of the focus group, we will ask verbal permission to record prior to proceeding. If an individual declines the audio recording, they will not be eligible for participation in the focus group. Participants may drop out at any time before, during, or after the focus group.

Clinic Staff: We will request a waiver of documentation of informed consent for participation in the implementation activities (clinic self-assessment working session) and the stakeholder focus groups, given the low risk nature of these activities. Participants in each of these sessions will be provided with written information describing the research, their participation, the risks, possible benefits, and all other required elements of informed consent. Because a signed consent form would be the only documentation of the participants' identities, we feel a waiver of documentation of informed consent is appropriate. At time of the working session and/or focus group, we will ask verbal permission to record prior to proceeding. If an individual declines the audio recording, they will not be eligible for participation in the focus group. Participants may drop out at any time before, during, or after the session.

13.4 Waiver of elements of informed consent—surgeons

We request a waiver of specific elements of consent regarding medical treatment, medical risks, costs, and injury to surgeon participants. This study does not involve medical treatment of surgeons, and these elements of informed consent would not apply to surgeons participating in this study. We have therefore removed these sections from the model consent form. Omission of this information does not constitute greater than minimal risk and would not adversely affect the surgeon participant.

13.5 Other people present during audiotaped visits

Individuals other than the patient and attending surgeon may be present during the audiotaped clinic visit. This includes family members, residents, nurses and medical students. Although

they are not study participants, they will receive written notification that the visit will be or is currently being recorded. We will allow individuals who do not verbally agree to be audiotaped to step out of the room during the time the audio recorder is on. This approach has been previously used with patients considering high risk operations and it has been tolerated well by their companions and other clinic staff. However, if individuals who do not verbally agree to be audiotaped do not wish to leave the room, we will not audio record the visit, out of sensitivity to the patient's needs.

14.0 REFERENCES

1. Joseph-Williams N, Elwyn G, Edwards A. Knowledge is not power for patients: a systematic review and thematic synthesis of patient-reported barriers and facilitators to shared decision making. *Patient Educ Couns.* 2014;94(3):291-309.
2. Durand MA, Carpenter L, Dolan H, et al. Do interventions designed to support shared decision-making reduce health inequalities? A systematic review and meta-analysis. *PLoS One.* 2014;9(4):e94670.
3. Oshima Lee E, Emanuel EJ. Shared decision making to improve care and reduce costs. *N Engl J Med.* 2013;368(1):6-8.
4. Stacey D, Legare F, Col NF, et al. Decision aids for people facing health treatment or screening decisions. *Cochrane Database Syst Rev.* 2014;1:CD001431.
5. Trikalinos TA, Wieland LS, Adam GP, et al. *Decision Aids for Cancer Screening and Treatment*. Rockville (MD)2014.
6. Waljee JF, Rogers MA, Alderman AK. Decision aids and breast cancer: do they influence choice for surgery and knowledge of treatment options? *J Clin Oncol.* 2007;25(9):1067-1073.
7. Fisher B, Anderson S, Bryant J, et al. Twenty-year follow-up of a randomized trial comparing total mastectomy, lumpectomy, and lumpectomy plus irradiation for the treatment of invasive breast cancer. *N Engl J Med.* 2002;347(16):1233-1241.
8. Veronesi U, Cascinelli N, Mariani L, et al. Twenty-year follow-up of a randomized study comparing breast-conserving surgery with radical mastectomy for early breast cancer. *N Engl J Med.* 2002;347(16):1227-1232.
9. Frisell A, Lagergren J, de Boniface J. National study of the impact of patient information and involvement in decision-making on immediate breast reconstruction rates. *Br J Surg.* 2016;103(12):1640-1648.
10. Martinez KA, Resnicow K, Williams GC, et al. Does physician communication style impact patient report of decision quality for breast cancer treatment? *Patient Educ Couns.* 2016;99(12):1947-1954.
11. Mandelblatt J, Kreling B, Figeuriedo M, et al. What is the impact of shared decision making on treatment and outcomes for older women with breast cancer? *J Clin Oncol.* 2006;24(30):4908-4913.
12. Frongillo M, Feibelmann S, Belkora J, et al. Is there shared decision making when the provider makes a recommendation? *Patient Educ Couns.* 2013;90(1):69-73.
13. Mandelblatt JS, Berg CD, Meropol NJ, et al. Measuring and predicting surgeons' practice styles for breast cancer treatment in older women. *Med Care.* 2001;39(3):228-242.
14. Greenberg CC, Schneider EC, Lipsitz SR, et al. Do variations in provider discussions explain socioeconomic disparities in postmastectomy breast reconstruction? *J Am Coll Surg.* 2008;206(4):605-615.
15. Morrow M, Jagsi R, Alderman AK, et al. Surgeon recommendations and receipt of mastectomy for treatment of breast cancer. *JAMA.* 2009;302(14):1551-1556.
16. Christian CK, Niland J, Edge SB, et al. A multi-institutional analysis of the socioeconomic determinants of breast reconstruction: a study of the National Comprehensive Cancer Network. *Ann Surg.* 2006;243(2):241-249.
17. Jacobs LK, Kelley KA, Rosson GD, et al. Disparities in urban and rural mastectomy populations : the effects of patient- and county-level factors on likelihood of receipt of mastectomy. *Ann Surg Oncol.* 2008;15(10):2644-2652.
18. Lautner M, Lin H, Shen Y, et al. Disparities in the Use of Breast-Conserving Therapy Among Patients With Early-Stage Breast Cancer. *JAMA Surg.* 2015;150(8):778-786.

19. Jagsi R, Jiang J, Momoh AO, et al. Trends and variation in use of breast reconstruction in patients with breast cancer undergoing mastectomy in the United States. *J Clin Oncol.* 2014;32(9):919-926.
20. Sisco M, Du H, Warner JP, et al. Have we expanded the equitable delivery of postmastectomy breast reconstruction in the new millennium? Evidence from the national cancer data base. *J Am Coll Surg.* 2012;215(5):658-666; discussion 666.
21. Moyer A. Psychosocial outcomes of breast-conserving surgery versus mastectomy: a meta-analytic review. *Health Psychol.* 1997;16(3):284-298.
22. Stevens LA, McGrath MH, Druss RG, et al. The psychological impact of immediate breast reconstruction for women with early breast cancer. *Plastic and Reconstructive Surgery.* 1984;73:619 - 628.
23. Schain WS. Breast reconstruction. Update of psychosocial and pragmatic concerns. . *Cancer.* 1991;68:1170 - 1175.
24. Elder EE, Brandberg Y, Bjorkund T, et al. Quality of life and patient satisfaction in breast cancer patients after immediate brast reconstruction: a prospective study. *Breast.* 2005;14:201 - 208.
25. Jagsi R, Li Y, Morrow M, et al. Patient-reported Quality of Life and Satisfaction With Cosmetic Outcomes After Breast Conservation and Mastectomy With and Without Reconstruction: Results of a Survey of Breast Cancer Survivors. *Ann Surg.* 2015;261(6):1198-1206.
26. Janz NK, Mujahid M, Lantz PM, et al. Population-based study of the relationship of treatment and sociodemographics on quality of life for early stage breast cancer. *Qual Life Res.* 2005;14(6):1467-1479.
27. Greenberg CC, Lipsitz SR, Hughes ME, et al. Institutional variation in the surgical treatment of breast cancer: a study of the NCCN. *Ann Surg.* 2011;254(2):339-345.
28. Mujahid MS, Janz NK, Hawley ST, et al. The impact of sociodemographic, treatment, and work support on missed work after breast cancer diagnosis. *Breast Cancer Res Treat.* 2010;119(1):213-220.
29. Hawley ST, Fagerlin A, Janz NK, et al. Racial/ethnic disparities in knowledge about risks and benefits of breast cancer treatment: does it matter where you go? *Health Serv Res.* 2008;43(4):1366-1387.
30. Freedman RA, Kouri EM, West DW, et al. Racial/Ethnic Disparities in Knowledge About One's Breast Cancer Characteristics. *Cancer.* 2015;121(5):724-732.
31. Janz NK, Mujahid MS, Hawley ST, et al. Racial/ethnic differences in adequacy of information and support for women with breast cancer. *Cancer.* 2008;113(5):1058-1067.
32. Katz SJ, Lantz PM, Janz NK, et al. Patient involvement in surgery treatment decisions for breast cancer. *J Clin Oncol.* 2005;23(24):5526-5533.
33. Polacek GN, Ramos MC, Ferrer RL. Breast cancer disparities and decision-making among U.S. women. *Patient Educ Couns.* 2007;65(2):158-165.
34. Maly RC, Stein JA, Umezawa Y, et al. Racial/ethnic differences in breast cancer outcomes among older patients: effects of physician communication and patient empowerment. *Health Psychol.* 2008;27(6):728-736.
35. Belkora JK, Volz S, Teng AE, et al. Impact of decision aids in a sustained implementation at a breast care center. *Patient Educ Couns.* 2012;86(2):195-204.
36. Gentles SJ, Stacey D, Bennett C, et al. Factors explaining the heterogeneity of effects of patient decision aids on knowledge of outcome probabilities: a systematic review sub-analysis. *Syst Rev.* 2013;2:95.
37. Hoffman AS, Volk RJ, Saarimaki A, et al. Delivering patient decision aids on the Internet: definitions, theories, current evidence, and emerging research areas. *BMC Med Inform Decis Mak.* 2013;13 Suppl 2:S13.

38. Sinha G. Decision aids help patients but still are not widely used. *J Natl Cancer Inst.* 2014;106(7).
39. Elwyn G, Scholl I, Tietbohl C, et al. "Many miles to go ...": a systematic review of the implementation of patient decision support interventions into routine clinical practice. *BMC Med Inform Decis Mak.* 2013;13 Suppl 2:S14.
40. Brace C, Schmocker S, Huang H, et al. Physicians' Awareness and Attitudes Toward Decision Aids for Patients With Cancer. *Journal of Clinical Oncology.* 2010;28(13):2286-2292.
41. Graham ID, Logan J, O'Connor A, et al. A qualitative study of physicians' perceptions of three decision aids. *Patient Educ Couns.* 2003;50(3):279-283.
42. O'Donnell S, Cranney A, Jacobsen MJ, et al. Understanding and overcoming the barriers of implementing patient decision aids in clinical practice. *J Eval Clin Pract.* 2006;12(2):174-181.
43. Silvia KA, Ozanne EM, Sepucha KR. Implementing breast cancer decision aids in community sites: barriers and resources. *Health Expect.* 2008;11(1):46-53.
44. Silvia KA, Sepucha KR. Decision aids in routine practice: lessons from the breast cancer initiative. *Health Expect.* 2006;9(3):255-264.
45. Holmes-Rovner M, Valade D, Orlowski C, et al. Implementing shared decision-making in routine practice: barriers and opportunities. *Health Expect.* 2000;3(3):182-191.
46. Charles C, Gafni A, Whelan T. Self-reported use of shared decision-making among breast cancer specialists and perceived barriers and facilitators to implementing this approach. *Health Expect.* 2004;7(4):338-348.
47. Legare F, Ratte S, Gravel K, et al. Barriers and facilitators to implementing shared decision-making in clinical practice: update of a systematic review of health professionals' perceptions. *Patient Educ Couns.* 2008;73(3):526-535.
48. Feibelmann S, Yang TS, Uzogara EE, et al. What does it take to have sustained use of decision aids? A programme evaluation for the Breast Cancer Initiative. *Health Expect.* 2011;14 Suppl 1:85-95.
49. O'Connor AM, Llewellyn-Thomas HA, Flood AB. Modifying unwarranted variations in health care: shared decision making using patient decision aids. *Health Aff (Millwood).* 2004;Suppl Variation:VAR63-72.
50. Elwyn G, Rix A, Holt T, et al. Why do clinicians not refer patients to online decision support tools? Interviews with front line clinics in the NHS. *BMJ Open.* 2012;2(6).
51. Bates DW, Kuperman GJ, Wang S, et al. Ten commandments for effective clinical decision support: making the practice of evidence-based medicine a reality. *J Am Med Inform Assoc.* 2003;10(6):523-530.
52. Lee CJ, Ramírez AS, Lewis N, et al. Looking beyond the Internet: examining socioeconomic inequalities in cancer information seeking among cancer patients. *Health Commun.* 2012;27(8):806-817.
53. Perrin A, Duggan M. "Americans' Internet Access: 2000-2015." Pew Research Center. 2015.
54. Bruce JG, Tucholka JL, Steffens NM, et al. Feasibility of providing web-based information to breast cancer patients prior to a surgical consult. *J Cancer Educ.* 2017;In press.
55. Kilbourne AM, Neumann MS, Pincus HA, et al. Implementing evidence-based interventions in health care: application of the replicating effective programs framework. *Implement Sci.* 2007;2:42.
56. Belkora JK, Miller MF, Dougherty K, et al. The need for decision and communication aids: a survey of breast cancer survivors. *J Community Support Oncol.* 2015;13(3):104-112.
57. Hack TF, Degner LF, Parker PA. The communication goals and needs of cancer patients: a review. *Psychooncology.* 2005;14(10):831-845; discussion 846-837.

58. Waltz TJ, Powell BJ, Matthieu MM, et al. Use of concept mapping to characterize relationships among implementation strategies and assess their feasibility and importance: results from the Expert Recommendations for Implementing Change (ERIC) study. *Implement Sci.* 2015;10(1):109.
59. Kind AJ, Jencks S, Brock J, et al. Neighborhood socioeconomic disadvantage and 30-day rehospitalization: a retrospective cohort study. *Ann Intern Med.* 2014;161(11):765-774.
60. Singh GK. Area deprivation and widening inequalities in US mortality, 1969-1998. *Am J Public Health.* 2003;93(7):1137-1143.
61. Singh GK, Miller BA, Hankey BF. Changing area socioeconomic patterns in U.S. cancer mortality, 1950-1998: Part II--Lung and colorectal cancers. *J Natl Cancer Inst.* 2002;94(12):916-925.
62. Singh GK, Miller BA, Hankey BF, et al. Persistent area socioeconomic disparities in U.S. incidence of cervical cancer, mortality, stage, and survival, 1975-2000. *Cancer.* 2004;101(5):1051-1057.
63. Singh GK, Miller BA, Hankey BF, et al. Changing area socioeconomic patterns in U.S. cancer mortality, 1950-1998: Part I--All cancers among men. *J Natl Cancer Inst.* 2002;94(12):904-915.
64. Street RL, Jr., Voigt B. Patient participation in deciding breast cancer treatment and subsequent quality of life. Medical decision making : an international journal of the Society for Medical Decision Making. Jul-Sep 1997;17(3):298-306
65. Gordon HS, Street RL. How Physicians, Patients, and Observers Compare on the Use of Qualitative and Quantitative Measures of Physician-Patient Communication. *Eval Health Prof.* 2016.
66. Pecanac KE, Kehler JM, Brasel KJ, et al. It's big surgery: preoperative expressions of risk, responsibility, and commitment to treatment after high-risk operations. *Ann Surg.* 2014;259(3):458-463.
67. Kruser JM, Pecanac KE, Brasel KJ, et al. "And I think that we can fix it": mental models used in high-risk surgical decision making. *Ann Surg.* 2015;261(4):678-684.
68. Nabozny MJ, Kruser JM, Steffens NM, et al. Patient-reported Limitations to Surgical Buy-in: A Qualitative Study of Patients Facing High-risk Surgery. *Ann Surg.* 2016.
69. Unger JM, Hershman DL, Albain KS, et al. Patient income level and cancer clinical trial participation. *J Clin Oncol.* 2013;31(5):536-542.
70. Taylor AJ, Randall C. Process mapping: enhancing the implementation of the Liverpool Care Pathway. *Int J Palliat Nurs.* 2007;13(4):163-167.
71. NHS Modernisation Agency. Improvement Leaders Guide. Process Mapping, Analysis, and Redesign. 2005.
72. Ivers N, Jamtvedt G, Flottorp S, et al. Audit and feedback: effects on professional practice and healthcare outcomes. *Cochrane Database Syst Rev.* 2012;6:CD000259.
73. Audit Feedback tool. Available at: http://www.HIPxChange.org/AuditFeedback 2015.
74. Maly RC, Frank JC, Marshall GN, et al. Perceived efficacy in patient-physician interactions (PEPPI): validation of an instrument in older persons. *J Am Geriatr Soc.* 1998;46(7):889-894.
75. Street RL, Jr., Voigt B, Geyer C, Jr., et al. Increasing patient involvement in choosing treatment for early breast cancer. *Cancer.* 1995;76(11):2275-2285.
76. Street RL, Jr., Gordon HS, Ward MM, et al. Patient participation in medical consultations: why some patients are more involved than others. *Med Care.* 2005;43(10):960-969.
77. Degner LF, Sloan JA, Venkatesh P. The Control Preferences Scale. *Can J Nurs Res.* 1997;29(3):21-43.

78. Bennett C, Graham ID, Kristjansson E, et al. Validation of a preparation for decision making scale. *Patient Educ Couns.* 2010;78(1):130-133.
79. Legare F, Kearing S, Clay K, et al. Are you SURE?: Assessing patient decisional conflict with a 4-item screening test. *Can Fam Physician.* 2010;56(8):e308-314.
80. Krupat E, Rosenkranz SL, Yeager CM, et al. The practice orientations of physicians and patients: the effect of doctor-patient congruence on satisfaction. *Patient Educ Couns.* 2000;39(1):49-59.
81. 2008. Web Content Accessibility Guidelines (WCAG) 2.0. Available at: http://www.w3.org/TR/WCAG20/. Accessed February 28, 2017.
82. Wallston KA, Cawthon C, McNaughton CD, et al. Psychometric properties of the brief health literacy screen in clinical practice. *J Gen Intern Med.* 2014;29(1):119-126.
83. Patton MQ. 2002. *Qualitative Research & Evaluation Methods.* Thousand Oaks, CA: Sage Publications.
84. Novick G. Is there a bias against telephone interviews in qualitative research? *Res Nurs Health.* 2008;31(4):391-398.
85. Shuy RW. In-person versus telephone interviewing. In: Holstein JA, Gubrium JF, eds. *Inside interviewing: new lenses, new concerns.* Thousand Oaks: Sage Publications; 2003:175-193.
86. Hatch M, von Ehrenstein O, Wolff M, et al. Using qualitative methods to elicit recall of a critical time period. *J Womens Health.* 1999;8(2):269-277.
87. Hack TF, Degner LF, Watson P, et al. Do patients benefit from participating in medical decision making? Longitudinal follow-up of women with breast cancer. *Psychooncology.* 2006;15(1):9-19.
88. Andersen MR, Bowen DJ, Morea J, et al. Involvement in decision-making and breast cancer survivor quality of life. *Health Psychol.* 2009;28(1):29-37.
89. Malterud K. Qualitative research: standards, challenges, and guidelines. *Lancet.* 2001;358(9280):483-488.
90. Yamazaki H, Slingsby BT, Takahashi M, et al. Characteristics of qualitative studies in influential journals of general medicine: a critical review. *Biosci Trends.* 2009;3(6):202-209.
91. Creswell JW, Plano-Clark WL. 2011. *Designing and Conducting Mixed Methods Research.* 2nd ed. Thousand Oaks, California: SAGE Publications.
92. Van de Ven AH, Delbecq AL. The nominal group as a research instrument for exploratory health studies. *Am J Public Health.* 1972;62(3):337-342.
93. Delbecq AL, Van De Ven A. 1975. *Group Techniques for Program Planning: A Guide to Nominal Groups and Delphi Process.* Glenview, IL: Scott Foresman Company.
94. O'Connor AM, Cranney A. User Manual - Acceptability. 1996. http://decisionaid.ohri.ca/docs/develop/User_Manuals/UM_Acceptability.pdf. Accessed July 12, 2013.
95. McCarthy DM, Buckley BA, Engel KG, et al. Understanding patient-provider conversations: what are we talking about? *Acad Emerg Med.* 2013;20(5):441-448.
96. Cheng SWM, Alison J, Dennis S, et al. A behaviour change intervention to reduce sedentary time in people with chronic obstructive pulmonary disease: protocol for a randomised controlled trial. *J Physiother.* 2017.
97. *Mplus* [computer program]. Los Angeles, CA.
98. Hussey MA, Hughes JP. Design and analysis of stepped wedge cluster randomized trials. *Contemp Clin Trials.* 2007;28(2):182-191.
99. ten Klooster PM, Oostveen JC, Zandbelt LC, et al. Further validation of the 5-item Perceived Efficacy in Patient-Physician Interactions (PEPPI-5) scale in patients with osteoarthritis. *Patient Educ Couns.* 2012;87(1):125-130.

100. MacKinnon DP, Lockwood CM, Hoffman JM, et al. A comparison of methods to test mediation and other intervening variable effects. *Psychol Methods.* 2002;7(1):83-104.

101. Fritz MS, Mackinnon DP. Required sample size to detect the mediated effect. *Psychol Sci.* 2007;18(3):233-239.

102. Fritz MS, Taylor AB, Mackinnon DP. Explanation of Two Anomalous Results in Statistical Mediation Analysis. *Multivariate Behav Res.* 2012;47(1):61-87.

103. Kenny DA. Mediation: PowMedR. Available at: http://davidakenny.net/webinars/Mediation/PowMedR/PowMedR.html. Accessed February 28, 2017.

104. White IR, Royston P, Wood AM. Multiple imputation using chained equations: Issues and guidance for practice. *Stat Med.* 2011;30(4):377-399.

105. Schafer JL. 1997. *Analysis of incomplete multivariate data.* New York: Chapman & Hall.

106. Miles MB, Huberman AM. 1994. *An Expanded Sourcebook: Qualitative Data Analysis.* 2ne ed. Thousand Oaks, California: SAGE Publications, Inc.

107. Elo S, Kyngas H. The qualitative content analysis process. *J Adv Nurs.* 2008;62(1):107-115.

108. Hsieh HF, Shannon SE. Three approaches to qualitative content analysis. *Qual Health Res.* 2005;15(9):1277-1288.

15.0 MODEL INFORMED CONSENT FOR PATIENTS

Study Title for Participants: Increasing Patients' Engagement in Breast Cancer Surgery Decision-Making

Overview and Key Information

What am I being asked to do?

We are asking you to take part in a research study. This study has public funding from the National Cancer Institute (NCI), part of the National Institutes of Health (NIH) in the United States Department of Health and Human Services. We do research studies to try to answer questions about how to prevent, diagnose, and treat diseases like cancer.

We are asking you to take part in this research study because you have an appointment with your surgeon to discuss treatment for breast cancer.

Taking part in this study is your choice.

You can choose to take part or you can choose not to take part in this study. You also can change your mind at any time. Whatever choice you make, you will not lose access to your medical care or give up any legal rights or benefits.

This document has important information to help you make your choice. Take time to read it. Talk to the study staff, your family, or friends about the risks and benefits of taking part in the study. It's important that you have as much information as you need and that all your questions are answered. See the "Where can I get more information?" section for resources for more clinical trials and general cancer information.

Why is this study being done?

This study is being done to answer the following question:

How can we best help patients and their surgeons make decisions about breast cancer surgery?

We are doing this study because we want to better understand how to support patients in making their decision about breast cancer surgery.

What is the usual approach to my diagnosis?

The usual approach for patients who are not in a study is to get advice about breast cancer surgery from their doctor.

What are my choices if I decide not to take part in this study?

- You may choose to have the usual approach described above.
- You may choose to take part in a different research study, if one is available.

What will happen if I decide to take part in this study?

If you decide to take part in this study, you will be asked to complete surveys before and after your appointment with your surgeon. You will also be asked for permission to audio-record the conversation between you and your surgeon. We expect your participation will last less than 3 weeks.

Some study participants will be contacted at the very end of the study and asked to participate in an optional one-on-one interview or focus group. You can decide at that time whether you want to participate in that interview.

What are the risks and benefits of taking part in this study?

There are both risks and benefits to taking part in this study. It is important for you to think carefully about these as you make your decision.

Risks

You may have the following discomforts:

- Be asked sensitive or private questions about things you normally do not discuss.

More specifically:

- You may feel anxious knowing the conversation with your surgeon is being recorded or find it hard to express all of your concerns because of the presence of an audio recorder. If you feel uncomfortable about being audio recorded after the recording has begun, tell the surgeon and he or she will immediately stop the recording.
- It is also possible that you may have some anxiety from answering questions about your treatment decision during the surveys with study staff. We recognize that this is a sensitive subject and some people would prefer not to talk about these issues. If you feel uncomfortable or nervous while completing the surveys, tell the study staff and he or she will immediately stop the survey. In addition, you may skip any questions on the surveys that you feel uncomfortable answering.

- Lastly, there is a small risk that your study information could become known to someone who is not involved in performing or monitoring this study. However, we make every effort to protect your privacy.

Benefits

You are not expected to have any direct medical benefit from participating in this study.

However, it may help the study doctors learn more about how surgeons and patients make decisions about breast cancer surgery. This study may help the study doctors learn things that may help other people in the future.

If I decide to take part in this study, can I stop later?

Yes, you can decide to stop taking part in the study at any time.

If you decide to stop, let your study doctor know as soon as possible. You also may choose to stop participation or skip any questions in the surveys that you do not feel comfortable answering.

Your study doctor will tell you about new information or changes in the study that may affect your health or your willingness to continue in the study.

Are there other reasons why I might stop being in the study?

Yes. The study doctor may take you off the study if:

- The study is stopped by the National Cancer Institute (NCI), Institutional Review Board (IRB), or study sponsor (the Alliance). The study sponsor is the organization who oversees the study.
- Your health changes and the study is no longer in your best interest.

This study is conducted by the Alliance for Clinical Trials in Oncology, a national clinical research group supported by the National Cancer Institute. The Alliance is made up of cancer doctors, health professionals, and laboratory researchers, whose goal is to develop better treatments for cancer, to prevent cancer, to reduce side effects from cancer, and to improve the quality of life of cancer patients.

It is important that you understand the information in the informed consent before making your decision. Please read, or have someone read to you, the rest of this document. If there is anything you don't understand, be sure to ask your study staff or nurse.

What is the purpose of this study?

This study is being done to learn about how patients and their surgeons make decisions about breast cancer surgery. We are doing this study because we want better understand how to support patients in making the decision about breast cancer surgery.

There will be about 625 patients taking part in this study.

What are the study groups?

All patients in this study will receive the usual approach of care, which is to get advice about breast cancer treatment from their doctor.

What procedures are involved in this study?

If you speak English and decide to take part in this study, you will be asked to complete two surveys. The first survey will take place before your appointment with your surgeon. This survey will be completed in-person and is expected to take 5 to 10 minutes to finish. The second survey will take place after your appointment with the surgeon. The second survey will be sent to you by email within a few days of the surgeon appointment. You also have the option of completing it over the phone or on paper, if you prefer. The second survey will take 10 to 20 minutes to finish.

Since these surveys are being used for research, the responses you provide will not be shared with your doctor. If you have any serious health issues or other concerns, please talk with your doctor or nurse right away.

We will also audio-record the conservation between you and your surgeon. We are asking to audio record the conversation with your surgeon because this will be a private conversation between you and your surgeon. Study staff will listen to the recording and type the entire conversation. The typed conversation will not include your name or any other information that could identify you. Your surgeon will never hear the audio recording. Only study staff will hear the audio recording. The audio file will be destroyed when the study is completed.

Study staff will review your medical chart. We are doing this only to collect:

- Demographic information (such as age)
- Information related to the evaluation and treatment of breast cancer
- Information about other related health conditions

Some study participants will be contacted at the very end of the study after we have finished the procedures described in this consent form and asked to participate in an optional one-on-one interview or focus group. You can decide at that time whether you want to participate in the interview or focus group.

Researchers will use this information to better understand how to support patients in making the decision about breast cancer surgery.

Another way to find out what will happen to you during this study is to read the chart below. Start reading at the left side and read across to the right, following the lines and arrows.

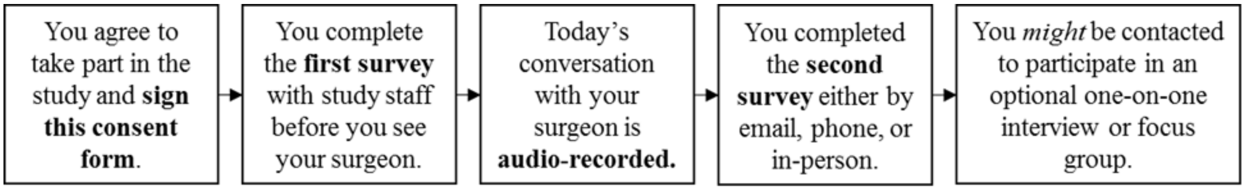

What are my responsibilities in this study?

If you choose to take part in this study, you will need to:

- Allow study staff to audio record the conversation between you and your surgeon.

- Complete two 10-15 minute surveys.
    - Before today's appointment (in-person), and
    - After today's appointment (either by email, over the phone, or in-person)

What are the costs of taking part in this study?

You and/or your insurance plan will need to pay for the costs of medical care you get as part of the study, just as you would if you were getting the usual care for your cancer.

Taking part in this study may mean that you need to make more visits to the clinic or hospital than if you were getting the usual approach to treat your cancer. You may:

- Have more travel costs.
- Need to take more time off work.
- Have other additional personal costs.

There are no expected costs to you for taking part in this study.
You will receive a gift card equivalent to $10 for audio recording the conversation with your surgeon and the completion of the first survey. You will then receive an additional gift card equivalent to $10 for completion of the second survey. The maximum compensation for participation in this study is $20.

Who will see my medical information?

Your privacy is very important to us. The study doctors will make every effort to protect it. However, some of your medical information may be given out if required by law. If this should happen, the study doctors will do their best to make sure that any information that goes out to others will not identify who you are.

Some of your health information, such as information related to the evaluation and treatment of your breast cancer, will be kept by the study sponsor in a central research database. If information from this study is published or presented at scientific meetings, your name and other personal information will not be used.

In order for the study researchers at the University of Wisconsin to be able to contact you about possibly participating in the telephone or in-person interviews and/or focus groups, it will be necessary for you to provide your name, address, and telephone number. Your contact information will be kept only for the duration of the study. In addition, if you choose to complete the follow up questionnaire by email, we will ask you to provide your email address for our database so that we can send you the link to the survey.

There are organizations that may look at or receive copies of some of the information in your study records. Your health information in the research database also may be shared with these organizations. They must keep your information private, unless required by law to give it to another group.

Some of these organizations are:

- The study sponsor (Alliance)
- University of Wisconsin (UW) and University of Texas (UT), where study data will be stored and/or analyzed.
- The NCI Central IRB, which is a group of people who review the research with the goal of protecting the people who take part in the study.
- The NCI and the groups it works with to review research.
- The NCI's National Clinical Trials Network and the groups it works with to conduct research

Your study records also will be stored for future use. However, your name and other personal information will not be used. Some types of future research may include looking at your records and those of other patients. However, right now we don't know what research may be done in the future using your information. This means that:

- You will not be asked if you agree to take part in the specific future research studies using your health information.
- You and your surgeon will not be told when or what type of research will be done.
- You will not get reports or other information about any research that is done using your information.

Where can I get more information?

You may visit the NCI web site at http://cancer.gov/ for more information about studies or general information about cancer. You may also call the NCI Cancer Information Service to get the same information at: 1-800-4-CANCER (1-800-422-6237).

A description of this clinical trial will be available on http://www.ClinicalTrials.gov, as required by U.S. Law. This Web site will not include information that can identify you. At most, the Web site will include a summary of the results. You can search this Web site at any time.

You can talk to the study staff about any questions or concerns you have about this study. Contact the study staff (*insert name of study staff*) at (*insert telephone number, and email address if appropriate*).

For questions about your rights while in this study, call the (*insert name of organization or center*) Institutional Review Board at (*insert telephone number*).

My signature agreeing to take part in the study

I have read this consent form or had it read to me. I have discussed it with the study doctor and my questions have been answered. I will be given a signed and dated copy of this form. I agree to take part in the main study.

Participant's signature

Date of signature

Signature of person(s) conducting the informed consent discussion

Date of signature

16.0 MODEL INFORMED CONSENT FOR SURGEONS

Title of Study: Increasing Patients' Engagement in Breast Cancer Surgery Decision Making Through a Shared Decision Making Intervention

Why am I being asked to participate?

You are invited to participate because you routinely assist in the care of those patients newly diagnosed with breast cancer. This study will enroll a total of 20-30 surgeons, 10 clinic staff and 625 patients across multiple sites in the United States. Approximately 2-3 surgeons and 109 patients will be enrolled at this institution.

This study is conducted by the Alliance for Clinical Trials in Oncology, a national clinical research group supported by the National Cancer Institute. The Alliance is made up of cancer doctors, health professionals, and laboratory researchers, whose goal is to develop better treatments for cancer, to prevent cancer, to reduce side effects from cancer, and to improve the quality of life of cancer patients.

Why is this study being done?

The objective of this study is to test the effectiveness of a decision aid in increasing patient engagement in SDM (Shared Decision Making) and identify barriers to engagement not mitigated by the decision aid that could be targets for adjunct SDM interventions.

What will my participation involve?

If you decide to participate in this research study, you will be asked to participate in an initial assessment meeting to identify possible barriers and facilitators of the implementation of the decision aid and complete one baseline survey before patient enrollment begins. This survey will ask about your attitudes towards shared decision making. Once patient enrollment begins, you will be asked to have your conversation about surgical treatment options with enrolled patients audio recorded. Patients may opt out of this audio recording. Your patients will go on to complete two short surveys.

At a certain time point in the study, you and your clinic will then be asked to move into the intervention arm. Once in the intervention group, we will send all your new patients who will be presenting for an appointment to discuss treatment decisions our intervention, a breast cancer surgeon decision aid. Again, you will be asked to have your conversation about surgical treatment options with enrolled patients audio recorded. Those patients will also complete two short surveys and possibly participate in an additional interview and/or focus group.

The determination of when you and your clinic will move over to the intervention group will be randomized. Please note that once you cross over to the decision aid intervention arm, your role in the study will not change and you will continue with your usual practice for seeing patients. Overall, we will enroll approximately 109 of your patients throughout a 2-year period. Your participation will require about 2 additional hours for participation in the implementation activities about 1 hour), surveys (about 10 minutes) and the audio-recordings.

Some surgeons will be contacted at the very end of the study after we have finished the procedures described in this consent form and asked to participate in an optional focus group. You can decide at that time whether you want to participate in the interview or focus group.

What are the risks of taking part in this study?

There is a risk that your study information could become known to someone who is not involved in performing or monitoring this study. It is also possible that you may feel anxious from knowing the conversations with your patients are being recorded and you may feel you are unable to express all of your concerns because of the presence of a recorder. If you feel uncomfortable about being audio recorded after the recording has begun, you may stop the audio recorder at any time.

If there are any patients you feel should not participate in the study, you will have the opportunity to tell study staff to exclude them from the study and they will not be approached by study staff. Lastly, there is a risk that when you cross into the intervention arm and patients begin using the intervention that your conversations with these patients may take a bit longer than usual due to increased patient participation.

A risk for your patients is developing some anxiety or clinical questions from participating in the surveys and interview and/or focus group with study staff. There is a chance they will want to speak with you or a member of your care team after a study survey or interview. If this requires an office visit or phone call, you will not be able to charge a fee if it is covered under the global payment for surgery.

What are the benefits of taking part in this study?

You are not expected to benefit directly from participating in this study. Your participation in this research study may benefit other people in the future by helping us learn more about breast cancer surgery decision making between surgeons and patients.

Will I be paid for my participation?

You will not receive any compensation for your participation in the study.

How will my confidentiality be protected?

Conversations that are recorded throughout your participation will be transcribed and coded with a study ID number. Only study staff and trained transcriptionists will have access to the audio files. All identifying information will be redacted in the written transcript. The audio files will be destroyed once the study is complete. Retained data (transcripts and surveys) will be de-identified once data collection is complete.

While there will likely be publications as a result of this study, your name will not be used. Only group characteristics will be published.
If you participate in this study, we would like to be able to quote the words you have used without using your name. If you agree to allow us to quote you in publications, please initial the statement at the bottom of this form.

A description of this clinical trial will be available on http://www.ClinicalTrials.gov, as required by U.S. Law. This Web site will not include information that can identify you. At most, the Web site will include a summary of the results. You can search this Web site at any time.

What if I have questions or concerns?

If you have any questions about this study at any time, contact the site Principal Investigator [PI NAME] at [PI PHONE NUMBER].

If you are not satisfied with the response of the research team, have more questions, or want to talk with someone about your rights as a research participant, contact the [SITE PATIENT RELATIONS REPRESENTATIVE] at [PHONE NUMBER].

Your participation is completely voluntary. If you decide not to participate or to withdraw from the study it will have no effect on any services or treatment you are currently receiving or giving or your professional standing.

Your signature indicates that you have read this consent form, had an opportunity to ask any questions about your participation in this research and voluntarily consent to participate. You will receive a copy of this form for your records.

Participant Name (please print): ____

____

Signature of Participant Date

I give my permission to be quoted directly in publications without using my name.
Initials

YOU WILL RECEIVE A COPY OF THIS FORM AFTER SIGNING IT.

Signature of person obtaining consent:

____

Signature Date

FUTURE RESEARCH

In the future, other researchers may find it valuable to use the transcripts of the audio recorded conversation obtained from this study. While we are only interested in the conversation to analyze how patients make treatment decisions that are the best for them, other investigators may be interested in different parts of the visit between patients and surgeons, for example, how do surgeons ask patients about their problems or how do surgeons comfort their patients. Because

the transcripts of the audiotapes have possible value to other researchers, we would like your permission to let other researchers use these transcripts for future research studies. These researchers would not have any information about who you are.

I give my permission for the use of the de-identified transcripts for future research:

______YES

______NO

APPENDIX I: USUAL CARE – PATIENT BASELINE SURVEY

You have been given a booklet to complete for this study. The booklet contains some questions we would like to ask you before you meet with your surgeon.

1. Directions on how to complete each set of questions are written on the top of each set.

2. You will be given the site coordinator's name and telephone number. You can call any time with any concerns or questions.

3. It is very important that you return the booklet to us.

4. Please hand the completed survey to the site coordinator.

Thank you for taking the time to help us.

PATIENT BASELINE (T0) SURVEY— USUAL CARE

Research Coordinator: Thank you for agreeing to participate in the study. I just have a few short questions I would like to ask you before you meet with your surgeon. This should take about 5 to 10 minutes to complete. And before we get started with those questions, I just have few things I would like to mention. First, your doctors and nurses know that you are helping us with this research but they will not be able to hear or see your responses to the questions I am going to ask you. Second, this is not a quiz; there is no right or wrong answer, I am just interested hearing your thoughts and feelings. Lastly, if you feel uncomfortable at any time, please let me know. Your participation in this study is voluntary, and you do not have to answer any questions that you do not want to.

Any questions before we start?

Great. Every person is different when it comes to how involved they are in making medical decisions. For example, some people say, “I’d rather have doctors make the decisions about what’s best than for the doctors to give me choices” and some people say “I’d rather be given choices about what’s best for my health than to have the doctor make the decisions for me.”

1. What is your preference for making decisions about your health care?

- ☐ I prefer to make the decision about which treatment I will receive.
- ☐ I prefer to make the final decision about my treatment after seriously considering my doctor’s opinion.
- ☐ I prefer that my doctor and I make the decision together about which treatment is best for me.
- ☐ I prefer that the doctor makes the final decision about treatment that will be used, while seriously considering my opinion.
- ☐ I prefer leaving all decisions about my treatment to my doctors.

2. On a scale from 0 to 10, where 10 means extremely well-informed and 0 means not informed at all, how informed do you feel about surgical options for your breast cancer?

**Not at all informed**										**Extremely well-informed**
0	1	2	3	4	5	6	7	8	9	10

This set of questions includes some reasons other women have given for choosing their breast cancer surgery. We are interested in what is important to you. On a scale from 0 to 10, how important are each of the following to you as you consider different surgery options for your breast cancer?

How important is it to you to…

		**Not at all important**										**Extremely Important**
**3.**	keep your breast?	0	1	2	3	4	5	6	7	8	9	10
**4.**	remove your entire breast in attempt to gain peace of mind?	0	1	2	3	4	5	6	7	8	9	10
**5.**	avoid having radiation	0	1	2	3	4	5	6	7	8	9	10

6. (*If you currently have a preference to treat your early stage breast cancer*), which treatment do you prefer?

- ☐ I am not sure
- ☐ Lumpectomy and radiation
- ☐ Lumpectomy only
- ☐ Mastectomy
- ☐ Other surgery

Other Surgery: ______________________________

7. Have you met with your oncologist to discuss treatment options prior to this visit with the surgeon?

- ☐ Yes
- ☐ No

(*If yes*)**,** who did you meet with?

- ☐ Another surgeon
- ☐ Medical oncologist
- ☐ Radiation oncologist
- ☐ Other

(If other), specify______________________________

8. Did you review any information before coming to see your surgeon today?

- ☐ Yes
- ☐ No

(*If yes*)**,** what types of information did you review before meeting your surgeon?

____

____

(*If yes*), how much time did you spend reviewing the information?

- ☐ Less than 15 minutes
- ☐ 15 - 60 minutes
- ☐ More than 1 hour

(*If no*), can you tell me why you did not review any information?

____

____

If patient is uncertain, offer categories to see if that prompts a response:

- ☐ I saw my surgeon very quickly after my diagnosis
- ☐ I didn’t look for any
- ☐ I was feeling too stressed
- ☐ Other ____

9. Did you have any trouble finding information?

- ☐ Yes
- ☐ No

(*If yes*)**,** can you tell me what was hard about it?

____

____

10. How confident are you filling out medical forms by yourself?

- ☐ Not at all
- ☐ A little bit
- ☐ Somewhat
- ☐ Quite a bit
- ☐ Extremely

11. How often do you have someone help you read hospital materials?

- ☐ Always
- ☐ Often
- ☐ Sometimes
- ☐ Occasionally
- ☐ Never

12. How often do you have problems learning about your medical condition because of difficulty understanding written information?

- ☐ Always
- ☐ Often
- ☐ Sometimes
- ☐ Occasionally
- ☐ Never

13. What is the preferred email address for us to send the follow-up survey?
NOTE: This information will be entered into the Rave electronic data entry system on the Patient Status: Baseline form by coordinator, and used only by a web service to send you the link for the follow-up survey.

____

14. What is the preferred mailing address for us to send the incentive after you complete the follow-up survey?
NOTE: This information below will NOT be entered into the Rave electronic data entry system.

____

APPENDIX II: INTERVENTION – PATIENT BASELINE SURVEY

You have been given a booklet to complete for this study. The booklet contains some questions we would like to ask you before you meet with your surgeon.

1. Directions on how to complete each set of questions are written on the top of each set.
2. You will be given the site coordinator's name and telephone number. You can call any time with any concerns or questions.
3. It is very important that you return the booklet to us.
4. Please hand the completed survey to the site coordinator.

Thank you for taking the time to help us.

PATIENT BASELINE (T0) SURVEY— INTERVENTION

Research Coordinator: Thank you for agreeing to participate in the study. I just have a few short questions I would like to ask you before you meet with your surgeon. This should take about 5 to 10 minutes to complete. And before we get started with those questions, I just have few things I would like to mention. First, your doctors and nurses know that you are helping us with this research but they will not be able to hear or see your responses to the questions I am going to ask you. Second, this is not a quiz; there is no right or wrong answer, I am just interested hearing your thoughts and feelings. Lastly, if you feel uncomfortable at any time, please let me know. Your participation in this study is voluntary, and you do not have to answer any questions that you do not want to.

Any questions before we start?

Great. Every person is different when it comes to how involved they are in making medical decisions. For example, some people say, “I’d rather have doctors make the decisions about what’s best than for the doctors to give me choices” and some people say “I’d rather be given choices about what’s best for my health than to have the doctor make the decisions for me.”

1. What is your preference for making decisions about your health care?

- ☐ I prefer to make the decision about which treatment I will receive.
- ☐ I prefer to make the final decision about my treatment after seriously considering my doctor’s opinion.
- ☐ I prefer that my doctor and I make the decision together about which treatment is best for me.
- ☐ I prefer that the doctor makes the final decision about treatment that will be used, while seriously considering my opinion.
- ☐ I prefer leaving all decisions about my treatment to my doctors.

2. On a scale from 0 to 10, where 10 means extremely well-informed and 0 means not informed at all, how informed do you feel about surgical options for your breast cancer?

**Not at all informed**										**Extremely well-informed**
0	1	2	3	4	5	6	7	8	9	10

This set of questions includes some reasons other women have given for choosing their breast cancer surgery. We are interested in what is important to you. On a scale from 0 to 10, how important are each of the following to you as you consider different surgery options for your breast cancer?

How important is it to you to . . .

	**Not at all important**										**Extremely Important**
**3.** keep your breast?	0	1	2	3	4	5	6	7	8	9	10
**4.** remove your entire breast in attempt to gain peace of mind?	0	1	2	3	4	5	6	7	8	9	10
**5.** avoid having radiation	0	1	2	3	4	5	6	7	8	9	10

6. (*If you currently have a preference to treat your early stage breast cancer*), which treatment do you prefer?

- ☐ I am not sure
- ☐ Lumpectomy and radiation
- ☐ Lumpectomy only
- ☐ Mastectomy
- ☐ Other surgery

Other surgery: ____

7. Have you met with your oncologist to discuss treatment options prior to this visit with the surgeon?

- ☐ Yes
- ☐ No

(*If yes*)**,** who did you meet with?

- ☐ Another surgeon
- ☐ Medical oncologist
- ☐ Radiation oncologist
- ☐ Other

(If other), specify_______________________________

8. Did the clinic offer to send you a website to review before coming to see your surgeon today?

- ☐ Yes

☐ No

If you answered "Yes" to question #8, please continue below.

If you answered "No" to question #8, please proceed directly to question #15.

9. Did you agree to have them send the website to you to review?

☐ Yes

☐ No

If you answered "Yes" to question #9, please continue below.

If you answered "No" to question #9, please proceed directly to question #15.

10. Were you able to review the website sent to you before coming to see your surgeon today?

☐ Yes

☐ No

(*If yes***),** how much time did you spend reviewing the website that was sent to you?

☐ Less than 15 minutes

☐ 15 - 60 minutes

☐ More than 1 hour

(*If no***)**, why did you not review the website?

____

____

If patient is uncertain, offer categories to see if that prompts a response:

☐ I saw my surgeon very quickly after receiving website

☐ I didn't have internet access between when I received the website and when I saw my surgeon

☐ I didn't receive the email

☐ I couldn't make the link to the website work

☐ I was feeling too stressed

☐ I didn't think I needed any information

☐ Other ____

11. Did you have any trouble opening or using the website?

☐ Yes

☐ No

*(If yes)***,** can you tell me what was hard about it?

____

____

12. Did you like getting the website sent to you by email?

☐ Yes

☐ No

(If no), what did you not like about it?

____

____

13. On a scale of 0 to 10, where 10 means very helpful and 0 means not at all helpful, how helpful did you find the breast cancer websites that were emailed to you?

**Not at all helpful**										**Very helpful**
0	1	2	3	4	5	6	7	8	9	10

14. Would you recommend receiving breast cancer information via email to other patients who are facing the decision for breast cancer surgery?

☐ I would recommend it

☐ I don’t have an opinion one way or the other

☐ I would not recommend it

15. How confident are you filling out medical forms by yourself?

☐ Not at all

☐ A little bit

☐ Somewhat

☐ Quite a bit

☐ Extremely

16. How often do you have someone help you read hospital materials?

- ☐ Always
- ☐ Often
- ☐ Sometimes
- ☐ Occasionally
- ☐ Never

17. How often do you have problems learning about your medical condition because of difficulty understanding written information?

- ☐ Always
- ☐ Often
- ☐ Sometimes
- ☐ Occasionally
- ☐ Never

18. What is the preferred email address for us to send the follow-up survey?
NOTE: This information will be entered into the Rave electronic data entry system on the Patient Status: Baseline form by coordinator, and used only by a web service to send you the link for the follow-up survey.

____

19. What is the preferred mailing address for us to send the incentive after you complete the follow-up survey?
NOTE: This information below will NOT be entered into the Rave electronic data entry system.

____

APPENDIX III: USUAL CARE – PATIENT FOLLOW-UP SURVEY

You have been given a booklet to complete for this study. The booklet contains some questions related to visits with your surgeon, about what was important to you when you thought about your decision for breast cancer surgery, and any materials that helped you make a decision. Your answers will help us to better understand how to support patients in making their decision about breast cancer surgery.

1. Directions on how to complete each set of questions are written on the top of each set.

2. You will be given the site coordinator's name and telephone number. You can call any time with any concerns or questions.

3. It is very important that you return the booklet to us.

4. Please hand the completed survey to the site coordinator or use the stamped and addressed envelope provided to mail the completed survey to the site coordinator. If we do not hear from you in the next week, we will contact you again.

Thank you for taking the time to help us.

PATIENT FOLLOW-UP (T2) SURVEY— USUAL CARE

Thank you for taking the time to complete this survey. This survey is about 50 questions and should take about 10 to 20 minutes to complete.

The first set of questions contains items that are related to visits with a surgeon. Please answer each statement to the best of your ability.

	Not at all confident (1)	(2)	(3)	(4)	Very confident (5)
**1. How confident are you in your ability to know what questions to ask a surgeon?**	❍	❍	❍	❍	❍
**2. How confident are you in your ability to get a surgeon to answer all of your questions?**	❍	❍	❍	❍	❍
**3. How confident are you in your ability to make the most of your visit with a surgeon?**	❍	❍	❍	❍	❍
**4. How confident are you in your ability to get a surgeon to take your chief health concerns seriously?**	❍	❍	❍	❍	❍
**5. How confident are you in your ability to get a surgeon to do something about your chief health concern?**	❍	❍	❍	❍	❍

The second set of questions contains items that are related to your recent visit with your surgeon. Surgeons have different styles in how they interact with patients and we would like to know more about how you felt about your visit with your surgeon. Please indicate how much you agree or disagree with each statement. Please answer to the best of your ability.

	Strongly disagree (1)	(2)	(3)	Neutral (4)	(5)	(6)	Strongly agree (7)
**6. I feel that my surgeon has provided me choices and options.**	❍	❍	❍	❍	❍	❍	❍
**7. I feel understood by my surgeon.**	❍	❍	❍	❍	❍	❍	❍
**8. I am able to be open with my surgeon at our meetings.**	❍	❍	❍	❍	❍	❍	❍
**9. My surgeon conveys confidence in my ability to make treatment choices.**	❍	❍	❍	❍	❍	❍	❍
**10. I feel that my surgeon accepts me.**	❍	❍	❍	❍	❍	❍	❍
**11. My surgeon has made sure I really understand about my condition and what I need to do.**	❍	❍	❍	❍	❍	❍	❍
**12. My surgeon encourages me to ask questions.**	❍	❍	❍	❍	❍	❍	❍
**13. I feel a lot of trust in my surgeon.**	❍	❍	❍	❍	❍	❍	❍
**14. My surgeon answers my questions fully and carefully.**	❍	❍	❍	❍	❍	❍	❍
**15. My surgeon listens to how I would like to do things.**	❍	❍	❍	❍	❍	❍	❍
**16. My surgeon handles people's emotions very well.**	❍	❍	❍	❍	❍	❍	❍
**17. I feel that my surgeon cares about me as a person.**	❍	❍	❍	❍	❍	❍	❍
**18. I don't feel very good about the way my surgeon talks to me.**	❍	❍	❍	❍	❍	❍	❍
**19. My surgeon tries to understand how I see things before suggesting how to do things.**	❍	❍	❍	❍	❍	❍	❍
**20. I feel able to share my feelings with my surgeon.**	❍	❍	❍	❍	❍	❍	❍

The next set of questions asks about some facts that surgeons think are important for patients to know about breast cancer. The correct answer to each question is based on medical research. Please answer to the best of your ability.

21. For most women with early breast cancer, how much would waiting a few weeks to make a treatment decision affect their chances of survival?

- ☐ A little or not at all
- ☐ A lot
- ☐ Some

22. With treatment, about how many women diagnosed with early breast cancer will eventually die of breast cancer?

- ☐ About half will die of breast cancer
- ☐ Most will die of breast cancer
- ☐ Most will die of something else

23. After which treatment is it more likely that women will need to have another operation to remove more tumor cells?

- ☐ Lumpectomy
- ☐ Mastectomy
- ☐ Equally likely for both

24. On average, which women with early breast cancer live longer?

- ☐ Women who have a mastectomy
- ☐ Women who have a lumpectomy and radiation
- ☐ There is no difference

25. On average, which women have a higher chance of having cancer come back in the breast that has been treated?

- ☐ Women who have a mastectomy
- ☐ Women who have a lumpectomy and radiation
- ☐ There is no difference

We are interested in what was important to you when you made the decision about breast cancer surgery. We asked you these same questions before you met with your surgeon, and are asking again to see if anything has changed. Please mark on a scale from 0 to 10, how important were each of the following to you as you make your decision about surgery.

How important was it to you to . . .

		Not at all important										Extremely Important
**26.**	keep your breast?	0	1	2	3	4	5	6	7	8	9	10
**27.**	remove your entire breast to gain peace of mind?	0	1	2	3	4	5	6	7	8	9	10
**28.**	avoid having radiation	0	1	2	3	4	5	6	7	8	9	10

29. (*Now that your clinic visit is over, if you currently have a preference to treat your early stage breast cancer*), which treatment do you prefer?

- ☐ I am not sure
- ☐ Lumpectomy and radiation
- ☐ Lumpectomy only
- ☐ Mastectomy
- ☐ Other surgery

Other surgery: ____

30. Do you feel sure about the best choice for you?

- ☐ Yes
- ☐ No

31. Do you know the benefits and risks of each option?

- ☐ Yes
- ☐ No

32. Are you clear about which benefits and risks matter most to you?

- ☐ Yes
- ☐ No

33. Do you have enough support and advice to make a choice?

☐ Yes

☐ No

The next questions ask about any materials that helped you prepare to meet with your surgeon and make a decision for breast cancer surgery.

34. Did you review any information <u>after</u> seeing your surgeon?

☐ Yes

☐ No

If you answered "No" to question #34, please proceed directly to question #35.

(*If yes),* what types of information did you review <u>after</u> meeting your surgeon?

____

____

(*If yes***)**, how much time did you spend reviewing the information?

☐ Less than 15 minutes

☐ 15 - 60 minutes

☐ More than 1 hour

If you answered "Yes" to question #34, please proceed directly to question #36.

If you answered "No" to question #34, please answer question #35.

35. Did you receive any information at <u>any time</u>?

☐ Yes, I reviewed information (either before or after seeing the surgeon).

☐ No, I did not review any information.

If you answered "Yes" to question #35, please answer question #36.

If you answered "No" to question #35, please proceed directly to question #48.

	Not at all	A little	Somewhat	Quite a bit	A great deal
**36. Did the information help you recognize that a decision needs to be made?**	1	2	3	4	5
**37. Did the information prepare you to make a better decision?**	1	2	3	4	5
**38. Did the information help you think about the pros and cons of each option?**	1	2	3	4	5
**39. Did the information help you think about which pros and cons are most important?**	1	2	3	4	5
**40. Did the information help you know that the decision depends on what matter most to you?**	1	2	3	4	5
**41. Did the information help you organize your own thoughts about the decision?**	1	2	3	4	5
**42. Did the information help you think about how involved you want to be in this decision?**	1	2	3	4	5
**43. Did the information help you identify questions you want to ask your surgeon?**	1	2	3	4	5
**44. Did the information prepare you to talk to your surgeon about what matters most to you?**	1	2	3	4	5
**45. Did the information prepare you for a follow-up visit with your surgeon?**	1	2	3	4	5

46. Where did you review the information? Check all that apply.

- ☐ On my own computer at home
- ☐ On a computer at my family or friend's home
- ☐ On my own tablet or mobile phone
- ☐ On my family or friend's tablet or mobile phone
- ☐ On a public computer (ex. library)
- ☐ On a iPad in clinic
- ☐ Other

(If other), specify________________________________

47. Did anyone else review the information? (Either with you or you sending them the information)

- ☐ Yes
- ☐ No

*(If yes)***,** who? Check all that apply.

- ☐ Partner/spouse
- ☐ Family member

(If family member), specify_______________________________

- ☐ Friend
- ☐ Other

(If other), specify_______________________________

The following questions are for all patients, regardless of whether they did or did not review any materials to help make a decision about the type of breast cancer surgery to have.

48. Would you like to receive information from the clinic?

- ☐ Yes
- ☐ No

49. What do you think would be the best format for you to receive and review information from the clinic?

- ☐ Paper version
- ☐ Computer-based version
- ☐ DVD-based version
- ☐ Other

(If other), specify_______________________________

50. If the clinic sent web-based material prior to the first appointment with the surgeon, what would be the best way to receive this information?

- ☐ By email
- ☐ By electronic medical record, such as using a patient portal (MyChart)
- ☐ By text
- ☐ By mail
- ☐ Other

(If other), specify_______________________________

Below are some questions so we can learn a little more about you. This information will help us understand how decision making between the surgeon and patient may be differ between patients, and how to better support patients in making their decision about breast cancer surgery.

1. What is the **highest** level of school or degree you have completed?
   - ☐ Some high school or less
   - ☐ High school diploma or GED
   - ☐ Vocational degree or some college
   - ☐ College degree
   - ☐ Graduate school degree or higher

2. Are you of Hispanic, Latino, or Spanish origin?
   - ☐ Yes
   - ☐ No

3. How would you describe your racial background?
   - ☐ White or Caucasian
   - ☐ Black or African American
   - ☐ Asian
   - ☐ American Indian of Alaska Native
   - ☐ Native Hawaiian or other Pacific Islander
   - ☐ Other

   (If other), specify_______________________________

4. What do you consider to be your primary language?
   - ☐ English
   - ☐ Spanish
   - ☐ Other

   (If other), specify_______________________________

5. What is your current marital status?
   - ☐ Single, never married
   - ☐ Married or domestic partnership
   - ☐ Widowed
   - ☐ Divorced
   - ☐ Separated

6. What is your household annual income?
   - ☐ <$25,000 per year
   - ☐ $25,000 - $49,999 per year
   - ☐ $50,000 - $99,999 per year
   - ☐ >$100,000 per year

APPENDIX IV: INTERVENTION – ACCEPTED DA – PATIENT FOLLOW-UP SURVEY

You have been given a booklet to complete for this study. The booklet contains some questions related to visits with your surgeon, about what was important to you when you thought about your decision for breast cancer surgery, and any materials that helped you make a decision. Your answers will help us to better understand how to support patients in making their decision about breast cancer surgery.

1. Directions on how to complete each set of questions are written on the top of each set.

2. You will be given the site coordinator’s name and telephone number. You can call any time with any concerns or questions.

3. It is very important that you return the booklet to us.

4. Please hand the completed survey to the site coordinator or use the stamped and addressed envelope provided to mail the completed survey to the site coordinator. If we do not hear from you in the next week, we will contact you again.

Thank you for taking the time to help us.

INTERVENTION ARM – ACCEPTED DECISION AID

PATIENT FOLLOW-UP (T2) SURVEY

Thank you for taking the time to complete this survey. This survey is about 50 questions and should take about 10 to 20 minutes to complete.

The first set of questions contains items that are related to visits with a surgeon. Please answer each statement to the best of your ability.

	Not at all confident (1)	(2)	(3)	(4)	Very confident (5)
1. How confident are you in your ability to know what questions to ask a surgeon?	❍	❍	❍	❍	❍
2. How confident are you in your ability to get a surgeon to answer all of your questions?	❍	❍	❍	❍	❍
3. How confident are you in your ability to make the most of your visit with a surgeon?	❍	❍	❍	❍	❍
4. How confident are you in your ability to get a surgeon to take your chief health concerns seriously?	❍	❍	❍	❍	❍
5. How confident are you in your ability to get a surgeon to do something about your chief health concern?	❍	❍	❍	❍	❍

The second set of questions contains items that are related to your recent visit with your surgeon. Surgeons have different styles in how they interact with patients and we would like to know more about how you felt about your visit with your surgeon. Please indicate how much you agree or disagree with each statement. Please answer to the best of your ability.

	Strongly disagree (1)	(2)	(3)	Neutral (4)	(5)	(6)	Strongly agree (7)
**6. I feel that my surgeon has provided me choices and options.**	❍	❍	❍	❍	❍	❍	❍
**7. I feel understood by my surgeon.**	❍	❍	❍	❍	❍	❍	❍
**8. I am able to be open with my surgeon at our meetings.**	❍	❍	❍	❍	❍	❍	❍
**9. My surgeon conveys confidence in my ability to make treatment choices.**	❍	❍	❍	❍	❍	❍	❍
**10. I feel that my surgeon accepts me.**	❍	❍	❍	❍	❍	❍	❍
**11. My surgeon has made sure I really understand about my condition and what I need to do.**	❍	❍	❍	❍	❍	❍	❍
**12. My surgeon encourages me to ask questions.**	❍	❍	❍	❍	❍	❍	❍
**13. I feel a lot of trust in my surgeon.**	❍	❍	❍	❍	❍	❍	❍
**14. My surgeon answers my questions fully and carefully.**	❍	❍	❍	❍	❍	❍	❍
**15. My surgeon listens to how I would like to do things.**	❍	❍	❍	❍	❍	❍	❍
**16. My surgeon handles people's emotions very well.**	❍	❍	❍	❍	❍	❍	❍
**17. I feel that my surgeon cares about me as a person.**	❍	❍	❍	❍	❍	❍	❍
**18. I don't feel very good about the way my surgeon talks to me.**	❍	❍	❍	❍	❍	❍	❍
**19. My surgeon tries to understand how I see things before suggesting how to do things.**	❍	❍	❍	❍	❍	❍	❍
**20. I feel able to share my feelings with my surgeon.**	❍	❍	❍	❍	❍	❍	❍

The next set of questions asks about some facts that surgeons think are important for patients to know about breast cancer. The correct answer to each question is based on medical research. Please answer to the best of your ability.

21. For most women with early breast cancer, how much would waiting a few weeks to make a treatment decision affect their chances of survival?

- ☐ A little or not at all
- ☐ A lot
- ☐ Some

22. With treatment, about how many women diagnosed with early breast cancer will eventually die of breast cancer?

- ☐ About half will die of breast cancer
- ☐ Most will die of breast cancer
- ☐ Most will die of something else

23. After which treatment is it more likely that women will need to have another operation to remove more tumor cells?

- ☐ Lumpectomy
- ☐ Mastectomy
- ☐ Equally likely for both

24. On average, which women with early breast cancer live longer?

- ☐ Women who have a mastectomy
- ☐ Women who have a lumpectomy and radiation
- ☐ There is no difference

25. On average, which women have a higher chance of having cancer come back in the breast that has been treated?

- ☐ Women who have a mastectomy
- ☐ Women who have a lumpectomy and radiation
- ☐ There is no difference

We are interested in what was important to you when you made the decision about breast cancer surgery. We asked you these same questions before you met with your surgeon, and are asking again to see if anything has changed. Please mark on a scale from 0 to 10, how important were each of the following to you as you make your decision about surgery.

How important was it to you to . . .

		Not at all important										Extremely Important
**26.**	keep your breast?	0	1	2	3	4	5	6	7	8	9	10
**27.**	remove your entire breast to gain peace of mind?	0	1	2	3	4	5	6	7	8	9	10
**28.**	avoid having radiation	0	1	2	3	4	5	6	7	8	9	10

29. (*Now that your clinic visit is over, if you currently have a preference to treat your early stage breast cancer*), which treatment do you prefer?

- ☐ I am not sure
- ☐ Lumpectomy and radiation
- ☐ Lumpectomy only
- ☐ Mastectomy
- ☐ Other surgery

Other surgery: ________________________________

30. Do you feel sure about the best choice for you?

- ☐ Yes
- ☐ No

31. Do you know the benefits and risks of each option?

- ☐ Yes
- ☐ No

32. Are you clear about which benefits and risks matter most to you?

- ☐ Yes
- ☐ No

33. Do you have enough support and advice to make a choice?

☐ Yes

☐ No

You received a link to a website from your clinic prior to seeing your surgeon. The next questions ask about how this material helped you prepare to meet with your surgeon and make a decision for breast cancer surgery.

34. Did you review the website sent to you <u>after</u> seeing your surgeon?

☐ Yes

☐ No

(If yes), how much time did you spend reviewing the website?

☐ Less than 15 minutes

☐ 15 - 60 minutes

☐ More than 1 hour

35. After your initial phone call when clinic staff offered you the website information, how important did you think it was for you to review the website sent to you?

☐ Very important

☐ Neither important or unimportant

☐ Not at all important

36. Why did you think it was important or not important for you to review the website?

____

____

____

____

____

37. Did you review the website sent to you at <u>any time</u>?

☐ Yes, I reviewed the website (either before or after seeing the surgeon).

☐ No, I did not review the decision aid.

If you answered "Yes" to questions #34 or #37, please continue directly to question 38.

If you answered "No" to questions #34 and #37, please go directly to question #51.

	Not at all	A little	Somewhat	Quite a bit	A great deal
**38. Did the website** help you recognize that a decision needs to be made?	1	2	3	4	5
**39**. **Did the website** prepare you to make a better decision?	1	2	3	4	5
**40. Did the website** help you think about the pros and cons of each option?	1	2	3	4	5
**41**. **Did the website** help you think about which pros and cons are most important?	1	2	3	4	5
**42**. **Did the website** help you know that the decision depends on what matters most to you?	1	2	3	4	5
**43**. **Did the website** help you organize your own thoughts about the decision?	1	2	3	4	5
**44. Did the website** help you think about how involved you want to be in this decision?	1	2	3	4	5
**45**. **Did the website** help you identify questions you want to ask your surgeon?	1	2	3	4	5
**46**. **Did the website** prepare you to talk to your surgeon about what matters most to you?	1	2	3	4	5
**47**. **Did the website** prepare you for a follow-up visit with your surgeon?	1	2	3	4	5

48. Where did you review the website? Check all that apply.

☐ On my own computer at home

☐ On a computer at my family or friend’s home

☐ On my own tablet or mobile phone

☐ On my family or friend’s tablet or mobile phone

☐ On a public computer (ex. library)

☐ On a iPad in clinic

☐ Other

(If other), specify________________________________

49. Did anyone else review the website? (Either with you or you sending them the link)

☐ Yes

☐ No

(If yes), who? Check all that apply.

☐ Partner/spouse

☐ Family member

(If family member), specify________________________________

☐ Friend

☐ Other

(If other), specify________________________________

50. Did you have any trouble opening or using the website?

☐ Yes

☐ No

*(If yes)***,** what was hard about it?

____

____

The following questions are for all patients, regardless of whether they did and did not review the website sent to them from the clinic.

51. Did you like getting the website sent to you by email?

☐ Yes

☐ No

*(If no)***,** what did you not like about it?

____

____

52. What do you think would be the best format for you to receive and review information from the clinic?

- ☐ Paper version
- ☐ Computer-based version
- ☐ DVD-based version
- ☐ Other

(If other), specify________________________________

53. If the clinic continued to send web-based material prior to the first appointment with the surgeon, what would be the best way to receive this material?

- ☐ By email
- ☐ By electronic medical record, such as using a patient portal (MyChart)
- ☐ By text
- ☐ By mail
- ☐ Other

(If other), specify________________________________

Below are some questions so we can learn a little more about you. This information will help us understand how decision making between the surgeon and patient may be differ between patients, and how to better support patients in making their decision about breast cancer surgery.

1. What is the **highest** level of school or degree you have completed?
   - □ Some high school or less
   - □ High school diploma or GED
   - □ Vocational degree or some college
   - □ College degree
   - □ Graduate school degree or higher

2. Are you of Hispanic, Latino, or Spanish origin?
   - □ Yes
   - □ No

3. How would you describe your racial background?
   - □ White or Caucasian
   - □ Black or African American
   - □ Asian
   - □ American Indian of Alaska Native
   - □ Native Hawaiian or other Pacific Islander
   - □ Other

     (If other), specify________________________________

4. What do you consider to be your primary language?
   - □ English
   - □ Spanish
   - □ Other

     (If other), specify________________________________

5. What is your current marital status?
   - □ Single, never married
   - □ Married or domestic partnership
   - □ Widowed
   - □ Divorced
   - □ Separated

6. What is your household annual income?
   - ☐ <$25,000 per year
   - ☐ $25,000 - $49,999 per year
   - ☐ $50,000 - $99,999 per year
   - ☐ >$100,000 per year

APPENDIX V: INTERVENTION – DECLINED DA – PATIENT FOLLOW-UP SURVEY

You have been given a booklet to complete for this study. The booklet contains some questions related to visits with your surgeon, about what was important to you when you thought about your decision for breast cancer surgery, and any materials that helped you make a decision. Your answers will help us to better understand how to support patients in making their decision about breast cancer surgery.

1. Directions on how to complete each set of questions are written on the top of each set.

2. You will be given the site coordinator's name and telephone number. You can call any time with any concerns or questions.

3. It is very important that you return the booklet to us.

4. Please hand the completed survey to the site coordinator or use the stamped and addressed envelope provided to mail the completed survey to the site coordinator. If we do not hear from you in the next week, we will contact you again.

Thank you for taking the time to help us.

INTERVENTION ARM – DECLINED DECISION AID

PATIENT FOLLOW-UP (T2) SURVEY

Thank you for taking the time to complete this survey. This survey is about 50 questions and should take about 10 to 20 minutes to complete.

The first set of questions contains items that are related to visits with a surgeon. Please answer each statement to the best of your ability.

	Not at all confident (1)	(2)	(3)	(4)	Very confident (5)
1. How confident are you in your ability to know what questions to ask a surgeon?	❍	❍	❍	❍	❍
2. How confident are you in your ability to get a surgeon to answer all of your questions?	❍	❍	❍	❍	❍
3. How confident are you in your ability to make the most of your visit with a surgeon?	❍	❍	❍	❍	❍
4. How confident are you in your ability to get a surgeon to take your chief health concerns seriously?	❍	❍	❍	❍	❍
5. How confident are you in your ability to get a surgeon to do something about your chief health concern?	❍	❍	❍	❍	❍

The second set of questions contains items that are related to your recent visit with your surgeon. Surgeons have different styles in how they interact with patients and we would like to know more about how you felt about your visit with your surgeon. Please indicate how much you agree or disagree with each statement. Please answer to the best of your ability.

	Strongly disagree (1)	(2)	(3)	Neutral (4)	(5)	(6)	Strongly agree (7)
**6. I feel that my surgeon has provided me choices and options.**	❍	❍	❍	❍	❍	❍	❍
**7. I feel understood by my surgeon.**	❍	❍	❍	❍	❍	❍	❍
**8. I am able to be open with my surgeon at our meetings.**	❍	❍	❍	❍	❍	❍	❍
**9. My surgeon conveys confidence in my ability to make treatment choices.**	❍	❍	❍	❍	❍	❍	❍
**10. I feel that my surgeon accepts me.**	❍	❍	❍	❍	❍	❍	❍
**11. My surgeon has made sure I really understand about my condition and what I need to do.**	❍	❍	❍	❍	❍	❍	❍
**12. My surgeon encourages me to ask questions.**	❍	❍	❍	❍	❍	❍	❍
**13. I feel a lot of trust in my surgeon.**	❍	❍	❍	❍	❍	❍	❍
**14. My surgeon answers my questions fully and carefully.**	❍	❍	❍	❍	❍	❍	❍
**15. My surgeon listens to how I would like to do things.**	❍	❍	❍	❍	❍	❍	❍
**16. My surgeon handles people's emotions very well.**	❍	❍	❍	❍	❍	❍	❍
**17. I feel that my surgeon cares about me as a person.**	❍	❍	❍	❍	❍	❍	❍
**18. I don't feel very good about the way my surgeon talks to me.**	❍	❍	❍	❍	❍	❍	❍
**19. My surgeon tries to understand how I see things before suggesting how to do things.**	❍	❍	❍	❍	❍	❍	❍
**20. I feel able to share my feelings with my surgeon.**	❍	❍	❍	❍	❍	❍	❍

The next set of questions asks about some facts that surgeons think are important for patients to know about breast cancer. The correct answer to each question is based on medical research. Please answer to the best of your ability.

21. For most women with early breast cancer, how much would waiting a few weeks to make a treatment decision affect their chances of survival?

- ☐ A little or not at all
- ☐ A lot
- ☐ Some

22. With treatment, about how many women diagnosed with early breast cancer will eventually die of breast cancer?

- ☐ About half will die of breast cancer
- ☐ Most will die of breast cancer
- ☐ Most will die of something else

23. After which treatment is it more likely that women will need to have another operation to remove more tumor cells?

- ☐ Lumpectomy
- ☐ Mastectomy
- ☐ Equally likely for both

24. On average, which women with early breast cancer live longer?

- ☐ Women who have a mastectomy
- ☐ Women who have a lumpectomy and radiation
- ☐ There is no difference

25. On average, which women have a higher chance of having cancer come back in the breast that has been treated?

- ☐ Women who have a mastectomy
- ☐ Women who have a lumpectomy and radiation
- ☐ There is no difference

We are interested in what was important to you when you made the decision about breast cancer surgery. We asked you these same questions before you met with your surgeon, and are asking again to see if anything has changed. Please mark on a scale from 0 to 10, how important were each of the following to you as you make your decision about surgery.

How important was it to you to . . .

		Not at all important										Extremely Important
		0	1	2	3	4	5	6	7	8	9	10
**26.**	keep your breast?											
**27.**	remove your entire breast to gain peace of mind?	0	1	2	3	4	5	6	7	8	9	10
**28.**	avoid having radiation	0	1	2	3	4	5	6	7	8	9	10

29. (*Now that your clinic visit is over, if you currently have a preference to treat your early stage breast cancer*), which treatment do you prefer?

- ☐ I am not sure
- ☐ Lumpectomy and radiation
- ☐ Lumpectomy only
- ☐ Mastectomy
- ☐ Other surgery

Other Surgery: ________________________________

30. Do you feel sure about the best choice for you?

- ☐ Yes
- ☐ No

31. Do you know the benefits and risks of each option?

- ☐ Yes
- ☐ No

32. Are you clear about which benefits and risks matter most to you?

- ☐ Yes
- ☐ No

33. Do you have enough support and advice to make a choice?

- ☐ Yes
- ☐ No

The next questions ask about any materials that helped you prepare to meet with your surgeon and make a decision for breast cancer surgery.

34. Did you review any information after seeing your surgeon?

- ☐ Yes
- ☐ No

If you answered "No" to question #34, please proceed directly to question #35.

(*If yes),* what types of information did you review after meeting your surgeon?
____
____

(If yes), how much time did you spend reviewing the information?

- ☐ Less than 15 minutes
- ☐ 15 - 60 minutes
- ☐ More than 1 hour

If you answered "Yes" to question #34, please proceed directly to Question #36.

If you answered "No" to question #34, please answer Question #35.

35. Did you review any information at any time?

- ☐ Yes, I reviewed information (either before or after seeing the surgeon).
- ☐ No, I did not review any information.

If you answered "Yes" to question #35, continue to question #36.

If you answered "No" to question #36, go directly to question #48.

	Not at all	A little	Somewhat	Quite a bit	A great deal
**36. Did the information help you recognize that a decision needs to be made?**	1	2	3	4	5
**37. Did the information prepare you to make a better decision?**	1	2	3	4	5
**38. Did the information help you think about the pros and cons of each option?**	1	2	3	4	5
**39. Did the information help you think about which pros and cons are most important?**	1	2	3	4	5
**40. Did the information help you know that the decision depends on what matters most to you?**	1	2	3	4	5
**41. Did the information help you organize your own thoughts about the decision?**	1	2	3	4	5
**42. Did the information help you think about how involved you want to be in this decision?**	1	2	3	4	5
**43. Did the information help you identify questions you want to ask your surgeon?**	1	2	3	4	5
**44. Did the information prepare you to talk to your surgeon about what matters most to you?**	1	2	3	4	5
**45. Did the information prepare you for a follow-up visit with your surgeon?**	1	2	3	4	5

46. Where did you review the information? Check all that apply.

- ☐ On my own computer at home
- ☐ On a computer at my family or friend’s home
- ☐ On my own tablet or mobile phone
- ☐ On my family or friend’s tablet or mobile phone
- ☐ On a public computer (ex. library)
- ☐ On a iPad in clinic
- ☐ Other

(If other), specify________________________________

47. Did anyone else review the information? (Either with you or you sending them the information)

- ☐ Yes
- ☐ No

(*If yes*), who? Check all that apply.

- ☐ Partner/spouse
- ☐ Family member

(If family member), specify________________________________

- ☐ Friend
- ☐ Other

(If other), specify________________________________

The following questions are for all patients, regardless of whether they did and did not review any materials to help make a decision about the type of breast cancer surgery to have.

48. Would you like to receive information from the clinic?

- ☐ Yes
- ☐ No

49. What do you think would be the best format for you to receive and review information from the clinic?

- ☐ Paper version
- ☐ Computer-based version
- ☐ DVD-based version
- ☐ Other

(If other), specify________________________________

50. If the clinic sent web-based material prior to the first appointment with the surgeon, what would be the best way to receive this information?

- ☐ By email
- ☐ By electronic medical record, such as using a patient portal (MyChart)
- ☐ By text
- ☐ By mail
- ☐ Other

(If other), specify________________________________

Below are some questions so we can learn a little more about you. This information will help us understand how decision making between the surgeon and patient may be differ between patients, and how to better support patients in making their decision about breast cancer surgery.

1. What is the **highest** level of school or degree you have completed?
   - □ Some high school or less
   - □ High school diploma or GED
   - □ Vocational degree or some college
   - □ College degree
   - □ Graduate school degree or higher

2. Are you of Hispanic, Latino, or Spanish origin?
   - □ Yes
   - □ No

3. How would you describe your racial background?
   - □ White or Caucasian
   - □ Black or African American
   - □ Asian
   - □ American Indian of Alaska Native
   - □ Native Hawaiian or other Pacific Islander
   - □ Other

   (If other), specify________________________________

4. What do you consider to be your primary language?
   - □ English
   - □ Spanish
   - □ Other

   (If other), specify________________________________

5. What is your current marital status?
   - □ Single, never married
   - □ Married or domestic partnership
   - □ Widowed
   - □ Divorced
   - □ Separated

6. What is your household annual income?
   - ☐ <$25,000 per year
   - ☐ $25,000 - $49,999 per year
   - ☐ $50,000 - $99,999 per year
   - ☐ >$100,000 per year

APPENDIX VI: SURGEON BASELINE SURVEY

You have been given a booklet to complete for this study. The booklet contains some questions related to the role of the patient within the clinical consultation. Your answers will help us to better understand how to support patients in making their decision about breast cancer surgery.

1. Directions on how to complete each set of questions are written on the top of each set.

2. You will be given the site coordinator's name and telephone number. You can call any time with any concerns or questions.

3. It is very important that you return the booklet to us.

4. Please hand the completed survey to the site coordinator.

Thank you for taking the time to help us.

SURGEON BASELINE SURVEY

The following questions focus on the role of the patient within the clinical consultations. You should think about the average breast cancer patient consult in your clinic when answering these questions.

	Strongly Disagree	Moderately Disagree	Slightly Disagree	Slightly Agree	Moderately Agree	Strongly Agree
1. The surgeon is the one who should decide what gets talked about during the consult.	❍	❍	❍	❍	❍	❍
2. Although health care is less personal these days, this is a small price to pay for medical advances.	❍	❍	❍	❍	❍	❍
3. The most important part of the standard consult for breast cancer is the physical exam.	❍	❍	❍	❍	❍	❍
4. It is often best for patients if they do not have a full explanation of their breast cancer.	❍	❍	❍	❍	❍	❍
5. Patients should rely on their surgeons' knowledge and not try to find out about their breast cancer on their own.	❍	❍	❍	❍	❍	❍
6. When surgeons ask a lot of questions about a patient's background, they are prying too much into personal matters.	❍	❍	❍	❍	❍	❍
7. If surgeons are truly good at diagnosis and treatment, the way they relate to patients is not that important.	❍	❍	❍	❍	❍	❍
8. Many patients continue asking questions even though they are not learning anything new.	❍	❍	❍	❍	❍	❍
9. Patients should be treated as if they were partners with the surgeon, equal in power and status.	❍	❍	❍	❍	❍	❍
10. Patients generally want reassurance rather than information about their breast cancer.	❍	❍	❍	❍	❍	❍
11. If a surgeon's primary tools are being open and warm, the surgeon will not have a lot of success.	❍	❍	❍	❍	❍	❍

**12. When patients disagree with their surgeon, this is a sign that the surgeon does not have the patient's respect and trust.**	❍	❍	❍	❍	❍	❍
**13. A surgical plan cannot succeed if it is in conflict with a patient's lifestyle or values.**	❍	❍	❍	❍	❍	❍
**14. Most patients want to get in and out of the surgeon's office as quickly as possible.**	❍	❍	❍	❍	❍	❍
**15. The patient must always be aware that the surgeon is in charge.**	❍	❍	❍	❍	❍	❍
**16. It is not that important to know a patient's culture and background in order to treat their breast cancer.**	❍	❍	❍	❍	❍	❍
**17. Humor is a major ingredient in the surgeon's treatment of the patient.**	❍	❍	❍	❍	❍	❍
**18. When patients look up information about their breast cancer on their own, this usually confuses more than it helps.**	❍	❍	❍	❍	❍	❍

19. How many years have you been in practice? ____________

20. Have you completed a breast or surgical oncology fellowship? Yes or No

21. What percentage of your clinical practice is comprised of breast cancer patients? __________

22. What is your gender? Male or Female

23. Are you of Hispanic, Latino, or Spanish origin? Yes or No

24. How would you describe your racial background?

- ☐ American Indian or Alaskan Native
- ☐ Asian
- ☐ Black or African American
- ☐ Native Hawaiian or Other Pacific Islander
- ☐ White
- ☐ Other: _______________

APPENDIX VII: PATIENT ACCESSIBILITY TELEPHONE INTERVIEW GUIDE

Increasing Patients' Engagement in Breast Cancer Surgery Decision Making
ALLIANCE A231701CD

Accessibility Interview Guide

For patients that <u>accepted</u> the decision aid but reported difficulty accessing or using the decision aid website.

*****This interview guide includes sample questions addressing several constant domains. Within each domain, the interviewer may probe the participant for more in-depth responses or clarification of the response. Throughout the interviews, these domains will remain constant but the EXACT QUESTIONS may be modified based on participant responses*****

Rave ID: ____

Date of interview: ____

<u>Introduction:</u>

Hello. My name is [Name]. Thank you for taking the time to talk with me today. I'm calling because you agreed to participate in a short interview. Are you still interested in talking?

- *If <u>NO</u>*: **Thank you for considering this. Have a great day!**

- *If <u>YES</u>*:

  Great. Your breast cancer surgeon and clinic team know that you are helping us with this research but they will not be able to hear or see the results of this interview.

  The decision for type of surgery is hard for many women diagnosed with breast cancer. The research team is interested in finding ways to help women who need to make this decision. Please let us know if you are uncomfortable or would prefer not to answer a specific question. This isn't a quiz; there are no right or wrong answers. We would just like to hear your thoughts and feelings.

  As we discussed when I first talked to you about the interview, we would like to audio-record the interview so that I can focus on our conversation rather than note-taking. Is that still all right with you?

<u>Interviewer Note</u>: If interviewee indicates that it is okay to begin, please start audio recorder. If they say it is not okay, proceed with the interview with expanded note-taking as you are able.

Is it OK if we get started? Please let me know if you have questions or concerns at any time.

QUESTION SET #1: PRESENTATION OF THE DECISION AID TO PATIENTS

Prior to meeting with your surgeon for the first time, you were offered the chance to review a website that had information about breast cancer surgery. We are interested in learning about how the clinic talked about the website with you. You probably would have talked to [Name] about the website.

1. **After [Name] described the website to you, were you interested in reviewing the website?**

   <u>Interviewer Note:</u>
   If participant says "I don't remember talking about the website," provide more reminder prompts about it and → *proceed to Question 2.*

   If participant still doesn't remember, → *go to QUESTION SET #2: METHOD OF DECISION AID DELIVERY.*

2. **From what you were told by [Name], in what ways did you think the website would be helpful?**

3. **If you were to recommend the website to another woman that had been diagnosed with breast cancer, what would you say to her to encourage her to look at the website?**

→ *Go to QUESTION SET #2: METHOD OF DECISION AID DELIVERY.*

QUESTION SET #2: METHOD OF DECISION AID DELIVER

4. *<u>Interviewer Note</u>:*
   If you <u>do not know</u> how the patient was sent the website, start with 4a.

   If you <u>do know</u>, start with 4b.

   4a. We know that patients who participated in the research study were given and used the website in different ways. Can you tell me about how you were given the website?

   <u>PROBE:</u>
   - **For example, some people had the website link sent to their email. Other people had it sent to a family member's email or accessed the website from a web link on a card. Others just reviewed the website in clinic. How were you given the website?**

   → *Go to next page.*

   4b. You were sent the website via [email to personal email, other way]. Is that correct?

   → *Go to next page.*

We are trying to learn the best way to get the website to women who have been diagnosed with breast cancer. In the next few questions, we are looking for your advice about the best way to do this.

If patient received website by EMAIL to PERSONAL email account:

5. What are the advantages of being sent the website through email?

6. What difficulties did you have getting the website by email?

7. Would you have preferred to have been given the website in a different way?

- *If NO, → proceed to Question #8.*
- *If YES:*

  7a. **How would have preferred to receive the website?**

  7b. **Why do you think that would be a better way to be given the website?**

→ proceed to Question #8.

8. How could we do a better job in sending out the website?

→ Go to next page.

If patient received website ANOTHER WAY (other than email to their personal account):

9. What are the advantages of getting the website by [insert method of receiving website from interview Question 4a or 4b]?

10. What difficulties did you have getting the website in this way?

11. Why did you chose to get the website this way?

PROBE if they say "I did not chose this method":

- **How would you have preferred to receive the website?**
- **Why would this be a better way to be given the website?**

12. How do you think the process of getting the website could be improved?

→ Go to next page.

Interviewer Note:

*If patient **did not** review the website prior to meeting the surgeon (Question 10 of the baseline survey), → go to QUESTION SET #3: REASONS FOR NOT REVIEWING PRIOR TO SURGEON CONSULT.*

*If patient **did** review the website prior to meeting the surgeon (Question 10 of the baseline survey), → go to QUESTION SET #4: PERCEIVED VALUE OF RECEIVING DECISION AID.*

QUESTION SET #3: REASONS FOR NOT REVIEWING PRIOR TO SURGEON

13. On one of the surveys that you completed for the research study, you said you did not review the website before meeting with your surgeon. You mentioned, "[insert patient response to Question 10 on baseline survey]". I'd like to understand that more.

Interviewer Note: Allow patient to make an initial response here.

Interviewer Note: Additional follow-up questions are based on patient's answer to Question 10 on the baseline survey. → Go to appropriate section below based on the patient's survey response and ask these questions if they are not covered in the patient's initial response to Question #13.

- *13a. Survey Response* **»»** *"I didn't have internet access between when I received the website and when I saw my surgeon."*
    - 13a.1. **How do you usually access the internet?**

      PROBE:
        - **Where do you access the internet? For example, some people use a computer at home. Others use a smart phone or go the local library.**
    - 13a.2. **What challenges do you have in accessing the internet?**

→ *Proceed to Question #14.*

- *<u>13b. Survey Response</u>* **»»** *"I was feeling too stressed."*

  We really want to learn more about this because this is something that many women feel. We know that some women who review the website actually feel less stressed after getting this information from their surgeon. We are trying to learn how to do a better job explaining to women how the website could help them with their feelings of stress.

  - 13b.1. **Why did you think that the website was something that would not be useful to you?**

  - 13b.2. **Is there something that we could have told you about the website that would have explained how the website could lessen your stress?**

  → *Proceed to Question #14.*

- *<u>13c. Survey Response</u>* **»»** *"I didn't think I needed any information."*

  - 13c.1. **I'd like to understand more about why you thought you didn't need any information. Can you tell me more about why you gave this answer?**

  → *Proceed to Question #14.*

- *<u>13d. Survey Response</u>* **»»** *"I saw my surgeon very quickly after receiving the website," "I didn't receive the email," or "I couldn't make the link to the website work," there are no further questions.*

  → *Proceed to Question #14.*

14. Did you end up reviewing the website after meeting with your surgeon?

If NO, → go to QUESTION SET #5 FINAL QUESTION AND WRAP UP.

If YES:

- 14a. **Now that you have had time to review the website, do you think it would have been helpful to review earlier after your diagnosis to help prepare you to meet with your surgeon?**

- 14b. **What could we change that would make the information more helpful?**

If nothing could be changed, → go to QUESTION SET #5 FINAL QUESTION AND WRAP UP.

If something could be changed:

14b.1. **Looking back, how could [Name] have explained the website differently to better show how it could help you prepare to meet with your surgeon?**

→ *Go to QUESTION SET #5 FINAL QUESTION AND WRAP UP.*

QUESTION SET #4: PERCEIVED VALUE OF RECEIVING DECISION AID

The next few questions are about ways we could potentially improve how we share information with patients about breast cancer surgery.

15. How was the information helpful to you in preparing to meet with your surgeon?

16. What could we change that would make the information more helpful?

17. What are your thoughts about the surgery clinic providing you this information before your first visit with the surgeon?

Interviewer Note: The next question relates to the patient's response to Question #11 on the baseline survey.

If patient answered "No" to Question #11 on baseline survey, → *go to QUESTION SET #5: FINAL QUESTIONS AND WRAP UP.*

If patient answered "Yes" to Question #11 on baseline survey, ask:

17a. I saw on your survey that you had trouble using the website. Please tell me more about that.

→ *Go to QUESTION SET #5: FINAL QUESTIONS AND WRAP UP.*

QUESTION SET #5: FINAL QUESTIONS AND WRAP UP

We have one final question. I'd like you to think about your own personal experience with being diagnosed with breast cancer and meeting with your surgeon.

18. What other things can you think of that would help you prepare to meet with your surgeon for the first time?

Thank you again for taking the time to talk with me. Is there anything I haven't asked you that you think is important for us to know?

-- END --

Increasing Patients' Engagement in Breast Cancer Surgery Decision Making
ALLIANCE A231701CD

Accessibility Interview Guide

For patients that declined the decision aid.

*****This interview guide includes sample questions addressing several constant domains. Within each domain, the interviewer may probe the participant for more in-depth responses or clarification of the response. Throughout the interviews, these domains will remain constant but the EXACT QUESTIONS may be modified based on participant responses*****

Rave ID: ____

Date of interview: ____________________________________

Introduction:

Hello. My name is [Name]. Thank you for taking the time to talk with me today. I'm calling because you agreed to participate in a short interview. Are you still interested in talking?

- *If NO*: **Thank you for considering this. Have a great day!**

- *If YES*:

  Great. Your breast cancer surgeon and clinic team know that you are helping us with this research but they will not be able to hear or see the results of this interview.

  The decision for type of surgery is hard for many women diagnosed with breast cancer. The research team is interested in finding ways to help women who need to make this decision. Please let us know if you are uncomfortable or would prefer not to answer any specific question. This isn't a quiz; there are no right or wrong answers. We would just like to hear your thoughts and feelings.

  As we discussed when I first talked to you about the interview, we would like to audio-record the interview so that I can focus on our conversation rather than note-taking. Is that still all right with you?

Interviewer Note: If interviewee indicates that it is okay to begin, please start audio recorder. If they say it is not okay, proceed with the interview with expanded note-taking as you are able.

Is it OK if we get started? Please let me know if you have questions or concerns at any time.

QUESTION SET #1: PRESENTATION OF THE DECISION AID TO PATIENTS

Prior to meeting with your surgeon for the first time, you were offered the chance to review a website that had information about breast cancer surgery. We know that you decided you were not interested in reviewing the website at that time.

However, we are interested in learning about how the clinic talked about the website with you. You probably would have talked to [Name] about the website.

1. **How did [Name] describe the website to you?**

   Interviewer Note: If they say "I don't remember talking about the website," provide more reminder prompts about it. If they still don't remember, → go to QUESTION SET #2: REASONS FOR DECLINING THE DECISION AID

2. **From what you were told by [Name], in what ways did you think the website would be helpful?**

→ Go to QUESTION SET #2: REASONS FOR DECLINING THE DECISION AID

QUESTION SET #2: REASONS FOR DECLINING THE DECISION AID

When the clinic discussed the website with you, you said "[insert reason from decline log]" when you were talking with the clinic staff. I'd like to understand that more.

Interviewer note: Allow patients to make an initial response here.

PROBE based on reason for decline if needed, personalized to their response.

3. **We are trying to learn the best way to get the website to women who have been diagnosed with breast cancer. Looking back, how could [Name] have explained the website differently to better show how it could help you prepare to meet with your surgeon?**

→ Go to QUESTION SET #3: OPPORTUNITIES TO CHANGE INFORMATION DELIVERY

QUESTION SET #3: OPPORTUNITIES TO CHANGE INFORMATION DELIVER

We are interested in improving how we share information with breast cancer patients to help them prepare to meet with their surgeon.

4. **Would you have preferred to get the information in a different way other than through a website, such as a paper booklet or a DVD?**

   If NO: → proceed to Question 6.

   If YES:

   5a. **What way would you prefer? Why do you think that way would be better for you?**

5. **In this research study, patients were offered the website before they met with their surgeon. I know that you were not interested in reviewing the website during this time window. When do you think it would have been a better time for you to be given information about breast cancer surgery?**

   → Go to QUESTION SET #4: FINAL QUESTIONS AND WRAP UP

QUESTION SET #4: FINAL QUESTIONS AND WRAP UP

We have one final question. I'd like you to think about your own personal experience with being diagnosed with breast cancer and meeting with your surgeon.

6. **What other things can you think of that would help you prepare to meet with your surgeon for the first time?**

Thank you again for taking the time to talk with me. Is there anything I haven't asked you that you think is important for us to know?

-- END --

APPENDIX VIII: PATIENT PREPARATORY/INTERACTIONAL IN-PERSON INTERVIEW GUIDE

This interview guide includes sample questions addressing several constant domains. Within each domain, the interviewer may probe the participant for more in-depth responses or clarification of the response. Throughout the interviews, these domains will remain constant but the EXACT QUESTIONS AND WORDING OF THE QUESTIONS may be modified based on participant responses

RAVE ID: ____

Location: ____

Date of completion: ____

Brief introduction:

Thank you for taking the time to talk with me today.

There are different surgical options available for women diagnosed with breast cancer. We are interested in further understanding how you learned about the different options for surgery. We also want to hear about the conversation you had with your surgeon about the different options. By understanding your thoughts and experiences, we hope that we can improve the experience around surgical decision making for women who are diagnosed with breast cancer in the future.

We would like to audio-tape the interview so that I don't have to take as many notes while I'm listening to you. Is that alright with you?

Do you have any questions before we get started?

[question set #1- goal: understand their feelings when coming into the visit, preparatory barriers]

I want to start by having you think back to when you first met with Dr. [surgeon name]. Based on our notes, this would have been on [consult date]. During this visit, you and Dr. [surgeon name] talked about the different surgery options for the breast cancer. Please tell me what your thoughts were about breast surgery before that first visit with Dr. [surgeon name].

What were you expecting would happen during the course of the visit with Dr. [surgeon]?

There is a lot to know about breast cancer and the surgery options. On the first survey you completed when you first met with Dr. [surgeon], we asked about how informed you felt coming into the visit. Please tell me more about how informed you were feeling about those surgery options when you came to clinic to meet with your surgeon for the first time.

I know from reviewing your survey that you said you would prefer to [make the decision yourself/ make the decision after considering surgeons recommendation/make decision with surgery/ have surgery make decision after considering your values/have surgeon make decision].

Women have different responses to that survey question, with some women wanting to be very involved in making the decision for breast cancer surgery and others wanting their surgeon to lead the way. Tell me about why you thought it would be best to [make the decision yourself/ make the decision after considering surgeons recommendation/make decision with surgery/ have surgery make decision after considering your values/have surgeon make decision].

[question set #2: goal- understand what was the conversation, how the surgeon involved them, figure out what was important to the patient (facilitative barriers)]

I know from reviewing the audio-recording of your first visit that Dr. [surgeon name] discussed the different options for surgery with you, things like mastectomy or lumpectomy. Please tell me what you remember talking about with Dr. [surgeon name] regarding these options for surgery.

When Dr. [surgeon name] was describing the options for breast cancer surgery during that first visit, what were your gut feelings about what kind of surgery you wanted to have?

Choose one of the following based on whether surgery received differed from that "gut feeling"

- I know from reviewing your records that you did end up having a [mastectomy/lumpectomy]. Please tell me about the conversation between you and Dr. [surgeon] that led to this decision.

  OR

- I know from reviewing your records that your final surgery differed from what you were initially thinking you would want. Please tell me what led to that change.

What, if anything, did Dr. [surgeon] do that made you feel you were part of the planning for your breast surgery?

Probes:

- How well did the surgeon listen to you during the conversation?
- What questions did the surgeon ask you?
- *We will include additional patient-specific probes drawn from their responses on the HCCQ*
- *Climate Questionnaire (follow-up survey)*

Please tell me a little bit about how confident you felt talking with your surgeon during the initial consultation.

Probes:

- What questions did you ask the surgeon during the conversation?
- What did your surgeon do that made you comfortable sharing your thoughts or “gut feelings” about breast cancer surgery?
- Looking back on that conversation, what did you not say then that you now wish you had said to Dr. [surgeon name]? What do you think kept you from saying that during the conversation?
- *Patient-specific additional probes drawn from responses on the HCCQ Climate Questionnaire (follow-up survey)*

When you left that first visit with Dr. [surgeon], did you feel like they understood what was important to you in relation to your breast cancer surgery?

What did the surgeon do that made you feel that way?

Probe:

- How did they show you that they knew what you wanted?
- *Patient-specific additional probes drawn from responses on the HCCQ Climate Questionnaire (follow-up survey)*

Thinking about everything that we talked about, do you feel like you got what you needed from the conversation with Dr. [surgeon]?

If no,

What did you need that you didn’t get?

[question set #3- relationship between individual barriers and decision aid based on survey responses]

Before meeting with Dr. [surgeon] for the first time, the surgery clinic shared a website link with you. The website is designed to help women who have been diagnosed with breast cancer and will need to undergo surgery prepare to meet with their surgeon. On your survey, you said that you reviewed the

website before your visit with Dr. [surgeon]. Our next set of questions asks specifically about this website and ways to improve it.

Chose one of the following based on whether the website was helpful or not:

You said on your survey that the website was <u>helpful</u> to you. Please tell us in what ways the website was helpful to you.

How did you use the website to get ready for your visit with Dr. [surgeon]?

OR

You said on your survey that the website was <u>not very helpful</u> to you. Please tell us why you did not find the website to be helpful to you.

How could we make the website better?

The website that you were sent tries to describe the different options for surgery. Some women feel more informed after reviewing the website, whereas others think there is still just so much to know. Tell me a little bit about your thoughts on how the website described the options for surgery.

Probes:

- How was the information helpful?
- In what ways did it fall short?

Additional question if patient responded to Question #1 on the Patient Baseline survey with "I prefer leaving all decisions about my treatment to my doctors".

Some women want to be more involved in making the decision for breast cancer surgery after reviewing the website. I know that you preferred [leaving all decisions about surgery to your doctors], even after reviewing the website. What changes could we make to the website that might have increased your desire to be more involved in the decision for the type of breast cancer surgery?

If you were talking to a friend who had recently been diagnosed with breast cancer, what would you suggest about how she should use the website?

The website is one resource that we have used to help women who are facing a decision for breast cancer surgery. We are interested in your thoughts about other things that could help these women.

Probes: Ask these based on their response to the earlier questions as a last probe about surgery options

- What things could we have done before you met with your surgeon that would have been helpful for you in getting ready for your visit with Dr. [surgeon]?
- Based on your experiences, what could be done to help women feel more informed?
- What other things could we have done before you met with your surgeon that would have helped you be more involved in making your decision for type of breast cancer surgery?

Final question:

What else should I know about your experience with making the decision for breast cancer surgery?

APPENDIX IX: PATIENT FOCUS GROUP GUIDE

*****This focus group guide includes sample questions addressing several constant domains. Within each domain, the facilitator may probe the participants for more in-depth responses or clarification of the response. Throughout the study, these domains will remain constant but the exact QUESTIONS may be modified based on participant's responses*****

Location: ____

Date of completion: ____

Brief introduction:
Thank you for taking the time to talk with me today. We greatly appreciate your participation in our study over the past few years. We are very excited to share with you some of our findings and get your feedback. We are holding this group to specifically explore opportunities to further improve how we support patients making decisions about breast cancer surgery.

Part 1: Brief Presentation of Overall Research Findings
We will present the main findings from Aim 1 and 2 of the study for context.

Part 2: Summary of Prioritized Barriers to Patient Engagement in Decision Making
We will next review the barriers to engagement that were prioritized through the analysis in Aims 1 and 2. To ensure that this discussion is as salient as possible to stakeholders, we will present each barrier using quotes from patients who experienced the barrier. We will briefly elicit participants' perceptions of how these barriers impede engagement as we anticipate that the group discussion will provide additional insight beyond what was achieved in the quantitative data and one-on-one interviews.

Part 3: Brainstorming of Potential Adjunct Interventions to Support Patient Engagement
We will then present participants with potential adjunct interventions that may target the barriers discussed in part 2; examples are presented in the Table. These possible interventions were identified through the existing literature. We will present them as a way to trigger a discussion. We will elicit feedback from stakeholders regarding the anticipated effectiveness and feasibility of these interventions in their clinical settings, and needed adaptations. We anticipate that adaptation will be necessary, as identified interventions are not likely to have been developed within clinics caring for a high proportion of disadvantaged patients. We also anticipate that participants will propose alternative interventions as part of this discussion.

Table. Examples of possible interventions

Intervention	Goal for Patient	Target Barrier
Delivery using electronic health record	Decrease barriers to access	Accessibility
Pre-consult pairing with decisional coach	Improve preparation	Preparatory
Intervention to increase family member participation	Increase confidence in interacting	Interactional
Intervention to increase surgeon facilitative behaviors	Increase perception that input is valued	Interactional

Part #4: Identify Interventions with Greatest Potential
We will have each participant rank the potential interventions. Rankings will be shared immediately with the group. We will facilitate discussion regarding broad discrepancies in ratings to ensure the perspectives are fully understood. We will then ask stakeholders to evaluate the three interventions with the greatest potential.

APPENDIX X: CLINIC STAKEHOLDER FOCUS GROUP GUIDE

****This focus group guide includes a basic outline of the focus group script. The exact content of the sections will be driven by the research findings. For each focus group, the overall outline and information shared will be the same. However, the flow of the focus group will depend on the interaction with participants. As a result, although the content will remain the same between groups, the exact questions will vary.*****

Location: ____

Date of completion: ____

Brief introduction:

Thank you for taking the time to talk with me today. We greatly appreciate your participation in our study over the past few years. We are very excited to share with you some of our findings and get your feedback. We are holding this group to specifically explore opportunities to further improve how we support patients making decisions about breast cancer surgery.

Part 1: Brief Presentation of Overall Research Findings

We will present the main findings from Aim 1 and 2 of the study for context.

Part 2: Summary of Prioritized Barriers to Patient Engagement in Decision Making

We will next review the barriers to engagement that were prioritized through the analysis in Aims 1 and 2. To ensure that this discussion is as salient as possible to stakeholders, we will present each barrier using quotes from patients who experienced the barrier. We will briefly elicit participants' perceptions of how these barriers impede engagement as we anticipate that the group discussion will provide additional insight beyond what was achieved in the quantitative data and one-on-one interviews.

Part 3: Brainstorming of Potential Adjunct Interventions to Support Patient Engagement

We will then present participants with potential adjunct interventions that may target the barriers discussed in part 2; examples are presented in the Table. These possible interventions were identified through the existing literature. We will present them as a way to trigger a discussion. We will elicit feedback from stakeholders regarding the anticipated effectiveness and feasibility of these interventions in their clinical settings, and needed adaptations. We anticipate that adaptation will be necessary, as identified interventions are not likely to have been developed within clinics caring for a high proportion of disadvantaged patients. We also anticipate that participants will propose alternative interventions as part of this discussion.

Table. Examples of possible interventions

Intervention	Goal for Patient	Target Barrier
Delivery using electronic health record	Decrease barriers to access	Accessibility
Pre-consult pairing with decisional coach	Improve preparation	Preparatory
Intervention to increase family member participation	Increase confidence in interacting	Interactional
Intervention to increase surgeon facilitative behaviors	Increase perception that input is valued	Interactional

Part #4: Identify Interventions with Greatest Potential

We will have each participant rank the potential interventions. Rankings will be shared immediately with the group. We will facilitate discussion regarding broad discrepancies in ratings to ensure the perspectives are fully understood. We will then ask stakeholders to evaluate the three interventions with the greatest potential.

APPENDIX XI: SURVEY & INTERVIEW THANK YOU LETTER

[DATE]

Dear [PARTICIPANT NAME],

Thank you for your participation in the *Increasing Patients' Engagement in Breast Cancer Surgery Decision-Making* study. Our goal for the research is to better understand how to support patients in making their decisions for breast cancer surgery. Your participation helps us to reach that aim.

We have enclosed a gift card to thank you for your time.

If you have any questions or concerns, please feel free to contact us at any time. You can reach us by phone at **[STUDY COORDINATOR PHONE NUMBER]** or via email at **[STUDY COORDINATOR EMAIL]**.

Thank you again for your help with the study!

Thank you,

[STUDY COODINATOR NAME]
[STUDY COORDINATOR CONTACT INFORMATION]

APPENDIX XII: EMAIL BODY FOR PATIENT QUALTRICS SURVEY

Subject line: Alliance A231701CD Follow-up Survey

Dear Participant,

Thank you for your participation in our research study entitled, “Increasing Patients’ Engagement in Breast Cancer Surgery Decision-Making”.

The link to complete the survey for this research study is below. If we do not hear from you in the next few days, we will contact you again. If you have any questions or concerns, please contact your site research coordinator who will be able to assist you.

<Qualtrics link here>

Sincerely,

Alliance A231701CD Study Team
